# Colloidal Synthesis Enables Indium Solubility Modulation in Liquid Gallium Nanoparticles for Temperature-Dependent Generation of CO_2_ Electrocatalytic Active Sites

**DOI:** 10.1021/jacs.6c11963

**Published:** 2026-08-07

**Authors:** Coline Boulanger, Pau Torruella, Krishna Kumar, Pascal Schouwink, Petru P. Albertini, Anna Loiudice, Raffaella Buonsanti

**Affiliations:** † Laboratory of Nanochemistry for Energy (LNCE), Institute of Chemical Sciences and Engineering (ISIC), 27218 École Polytechnique Fédérale de Lausanne, Sion CH-1950, Switzerland; ‡ Electron Spectrometry and Microscopy Laboratory (LSME), Institute of Physics (IPHYS), École Polytechnique Fédérale de Lausanne, Lausanne CH-1015, Switzerland; § Institute of Chemical Sciences and Engineering (ISIC), École Polytechnique Fédérale de Lausanne, Sion CH-1950, Switzerland

## Abstract

Bulk liquid metals (LMs) are unique stimulus-responsive dynamic materials that are appealing across different fields of science and technology, including catalysis. However, the low surface-to-volume ratio, limited atomic-scale design, and required ad-hoc reactor engineering of bulk LM limit their exploitation. Colloidal LM nanoparticles (NPs) can address all of these shortcomings; however, their synthetic chemistry is challenging and less advanced compared to other materials. Here, we advance the synthetic knowledge on LM NPs and propose a colloidal approach for the synthesis of monodispersed In–Ga NPs with tunable indium content via a balanced precursor reactivity strategy. The synthesis generates temperature- and voltage-responsive supercooled metastable NPs, which we leverage for CO_2_ electroreduction. The metastable NPs show a unique temperature-dependent modulation of active sites, which results in increasing formate production with temperature. This behavior opposes the temperature deactivation reported so far for solid CO_2_RR catalysts toward the same product.

## Graphical abstract

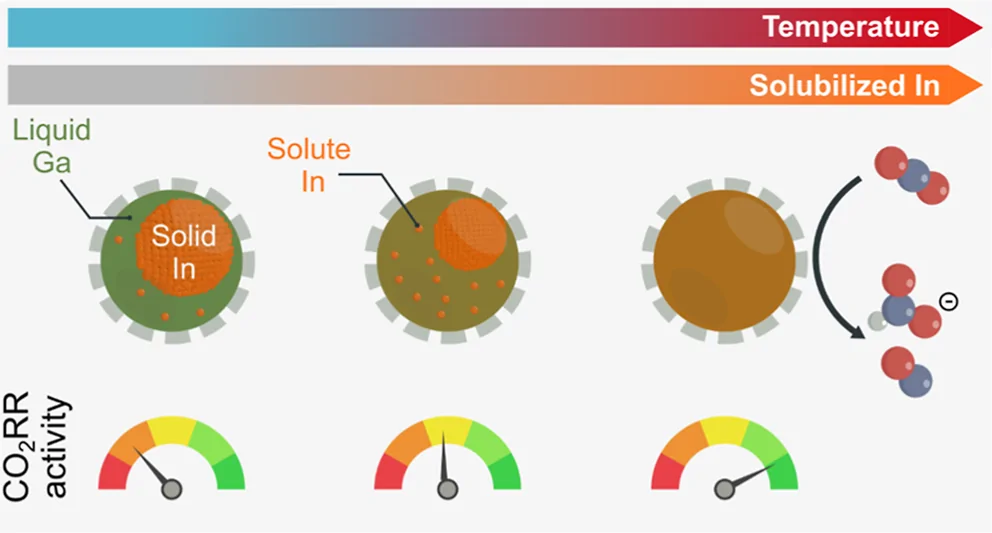

## Introduction

Stimulus-responsive dynamic materials are appealing across different fields of science and technology.^1–3^ Catalysis is no exception.^3^ The concept of self-regenerating catalysts relies on continuous dynamic changes of the surface wherein the catalytic active sites preserve their reactivity over time, which might benefit long-term operations.^4^ Regenerating strategies of solid catalysts exist in the literature.^4,5^ However, these strategies often rely on the addition of molecular additives or external stimuli rather than on intrinsic capability of the catalyst itself.^4^

Liquid metals (LMs) are metals or metal alloys, which possess a melting point below 300 °C.^6^ Among them, gallium-based LMs have raised interest in the past decade as they are liquid at or near room temperature while also possessing lower toxicity compared to mercury.^7^ By functioning as the catalytic material itself or as the solvent to catalytic entities, gallium-based LMs offer unique opportunities as self-regenerating and poisoning-resistant catalysts compared to conventional solid materials.^4,8–15^ In particular, LMs have recently emerged as one intriguing class of catalysts for the CO_2_ electroreduction reaction (CO_2_RR).^4,8,13–15^ Here, their liquid nature offers a practical solution against the reconstruction of solid metal catalysts, which currently hamper the long-term operational stability of this reaction.^4^ However, LM catalysts are generally used as bulk materials, which limits surface-to-volume ratio, complicates fundamental understanding via structure–property relationships, and requires ad-hoc engineering of the catalytic reactors.^4,13,16,17^

Colloidal nanoparticles (NPs) serve several purposes in catalysis science.^18–22^ First, NPs offer a high surface-to-volume ratio.^18–22^ Second, their tunable well-defined and monodisperse size and shape along with homogeneous composition advances fundamental understanding, which points at design strategies for several catalytic reactions.^18–22^ Finally, colloidal NPs come as an ink, which facilitates their integration into both thermal and electrocatalytic reactors.^18–22^

Colloidal LM NPs have been synthesized; however, the library of colloidal LM NPs remains limited compared to other classes of materials.^15,23^ The synthesis of these NPs is uniquely challenged by the nature of LMs, which includes a native oxide skin surrounding a liquid metal core with an ill-defined interface between them.^15,23,24^ This unique nature requires the development of ad-hoc synthesis strategies, combining design principles for oxides, liquids, and metals, to obtain LM NPs with well-defined and monodispersed size and shape along with a homogeneous composition.^15,23^ A few examples exist in the literature, which highlight the use of colloidal LM NPs in catalysis; for example, colloidal LM NPs function as CO_2_RR electrocatalysts with promising structural stability and easy integration into current electrochemical reactors.^4,14,15^ These examples motivate further exploitation of LM NPs, which requires expanding the current library available.

Herein, we demonstrate the synthesis of colloidal In–Ga NPs with tunable indium content thanks to a balanced metal precursor reactivity strategy. In situ temperature-dependent experiments demonstrate that colloidal synthesis generates a metastable liquid phase with high indium content. CO_2_RR experiments reveal that these metastable indium-enriched NPs turn into solid–liquid–solid structures during catalysis, with the indium solubilized in the gallium liquid phase being more active than solid indium NPs. Furthermore, the increase of the CO_2_RR production rate with temperature is uniquely different compared to what has been reported so far for solid CO_2_RR catalysts.

## Results and Discussion

### Colloidal Synthesis and Characterization of In–Ga NPs

First, we developed the synthesis of colloidal In–Ga NPs (Figures 1, S1–S6 and Note S1). The synthesis is based on a hot-injection method, wherein a gallium and indium precursors of balanced reactivity are co-injected into octadecene at 290 °C, followed by reaction at 260 °C for up to 1 h (Figure 1a). Increasing reaction time results into higher gallium contents (Figures S1 and S2). Experimental evidence indicates a seeded growth mechanism, in which gallium incorporation occurs on in situ forming indium seeds, along with suggesting the importance of the chosen metal precursors matching in reactivity either via redox chemistry or via a ligand-exchange process (Figure 1b, Note S1, and Figures S3–S6).

**Figure 1:**
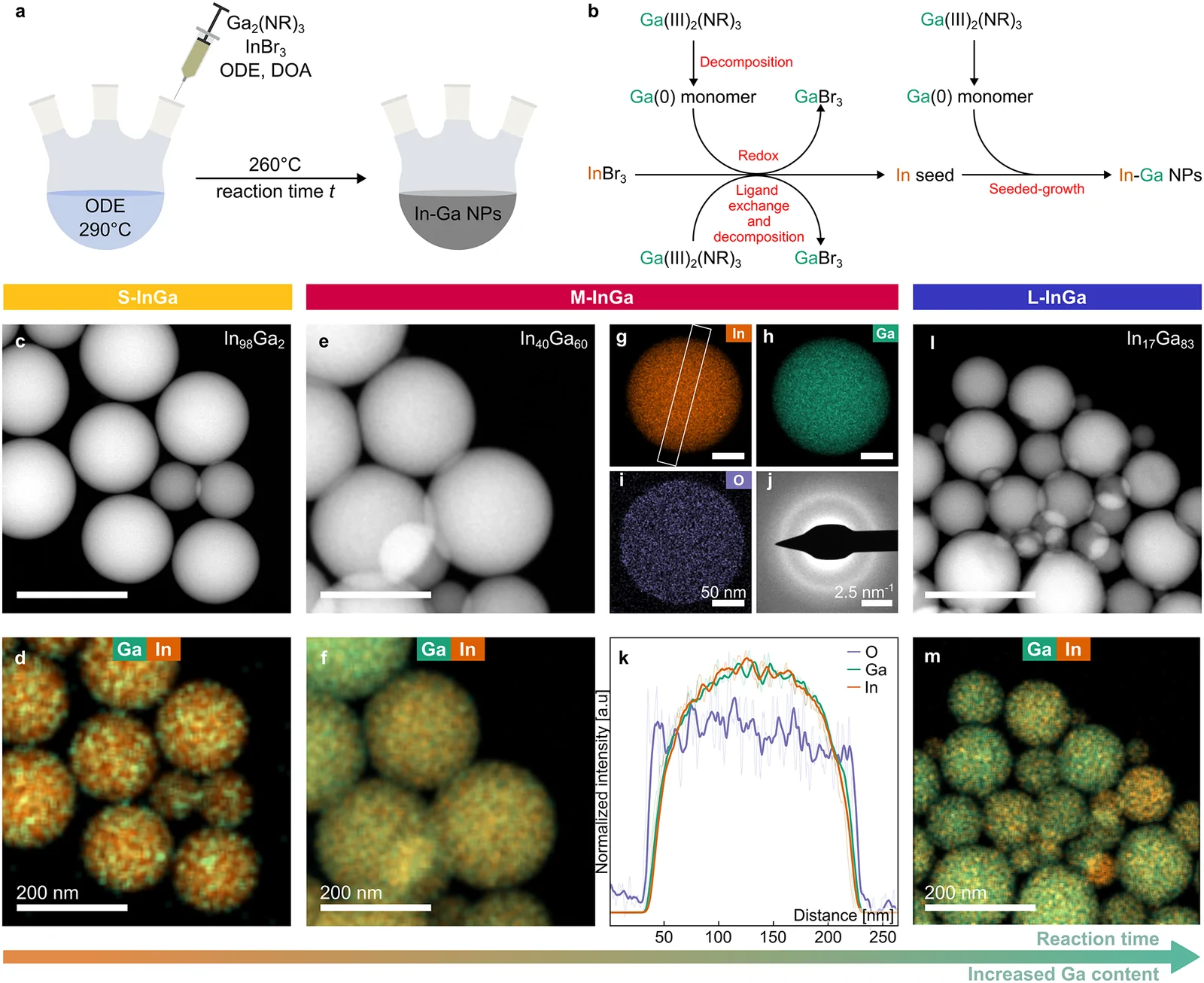
Colloidally synthesized In–Ga NPs. (a) Reaction scheme. (b) Proposed formation pathway. (c,d) HAADF-STEM image and corresponding EDXS elemental map for the In_98_Ga_2_ composition (S-InGa). (e,f) HAADF-STEM image and corresponding EDXS elemental map for the In40Ga60 composition (M-InGa). (g–i) Separated In, Ga, and O elemental maps of one M-InGa NP (orange: indium, green: gallium, purple: oxygen). (j) SAED showing no crystalline feature. (k) Line profile over one M-InGa NP (white rectangle in panel e). (l,m) HAADF-STEM image and corresponding EDXS elemental map for the In_17_Ga_83_ composition (L-InGa).

Herein, we focus on the synthesized In_98_Ga_2_, In_40_Ga_60_, and In_17_Ga_83_ compositions. The high-angle annular dark-field scanning transmission electron microscopy (HAADF-STEM) images evidence the spherical shape of the NPs, while the energy-dispersive X-ray spectroscopy (EDXS) elemental maps evidence the presence of both gallium and indium within the NPs (Figures 1c–m and S7–S9). Gallium and indium are homogeneously distributed within each NP (Figure 1d–m), and the native oxide shell surrounds each NP (Figures 1g and S7–S9). Selected area electron diffraction (SAED) (Figure S10) and X-ray diffraction (XRD) (Figure S11) indicate that In_98_Ga_2_ is solid, while In_40_Ga_60_ and In_17_Ga_83_ lack any crystalline features.

The bulk phase diagram indicates that In_98_Ga_2_ is a solid alloy, that In_40_Ga_60_ exists as a mixture of solid indium and a gallium-rich liquid phase, and that In_17_Ga_83_ is a liquid alloy, being close to the eutectic composition (Figure S12).^25^ STEM and XRD characterization of In_98_Ga_2_ and In_17_Ga_83_ NPs agree with the bulk phase diagram (Figures 1c,d,l,m, andS12). Conversely, the In_40_Ga_60_ NPs deviate from the bulk phase diagram as they appear as an amorphous alloy, most likely of liquid nature (Figures 1j and S10). On the basis of these considerations, we refer to the In_98_Ga_2_, In_40_Ga_60_, and In_17_Ga_83_ NPs as **S-InGa**, **M-InGa**, and **L-InGa**, respectively (S: solid, M: metastable, L: liquid).

The deviation from the bulk phase diagram of the **M-InGa** NPs prompted us to study their behavior under external stimuli, including temperature only and the combination of voltage and temperature in the context of the CO_2_RR catalysis.

To further probe the stability of the **M-InGa** NPs, we performed temperature-dependent in situ STEM experiment wherein we cooled down the NPs to −180 °C and imaged them at different increasing temperatures until returning to room temperature (Figures 2, S13–S17, and Note S2). HAADF-STEM coupled with EDXS shows that the **M-InGa** NPs (Figures 2a–d, S18, and 19) transform into a phase-segregated (solid indium + solid gallium) structure when cooled down to −180 °C (Figure 2e–h). These fully solid NPs convert into (solid indium + solid gallium) Janus NPs at −60 °C (Figure 2i–l), which then transition into (solid indium + gallium-rich liquid) core–shell NPs between −40 °C and −20 °C (Figure 2m–t), with the core occasionally observed moving in the liquid phase (Figures S15 and 16). The transition from solid gallium to gallium-rich liquid occurs at 15.3 °C in the bulk phase diagram (Figure S12).^25,26^ The deviation from the bulk behavior observed in the In–Ga NPs can be rationalized by the additional surface and interfacial energy contributions to the Gibbs free energy, which become significant at the nanoscale and depend on the composition and nature of the interfaces formed during the structural transitions.^27,28^ Then, the solid–liquid core–shell structure of the **M-InGa** remains upon return to room temperature, indicating that the as-synthesized colloidal **M-InGa** NPs are in a supercooled metastable fully liquid state. The **L-InGa** NPs undergo structural changes which are similar to those **M-InGa** (Figures S20 and 21). Interestingly, the electron diffraction hints at a transition from the γ-gallium solid phase into the β-gallium solid phase at −60 °C (Figures S20 and 21).^29^ We suggest that this transition might be contributing or triggering the transition into the Janus structure which occurs at this temperature. Unlike the **M-InGa** NPs, the **L-InGa** NPs return to their fully liquid state at −20 °C and remain liquid also at room temperature.

**Figure 2:**
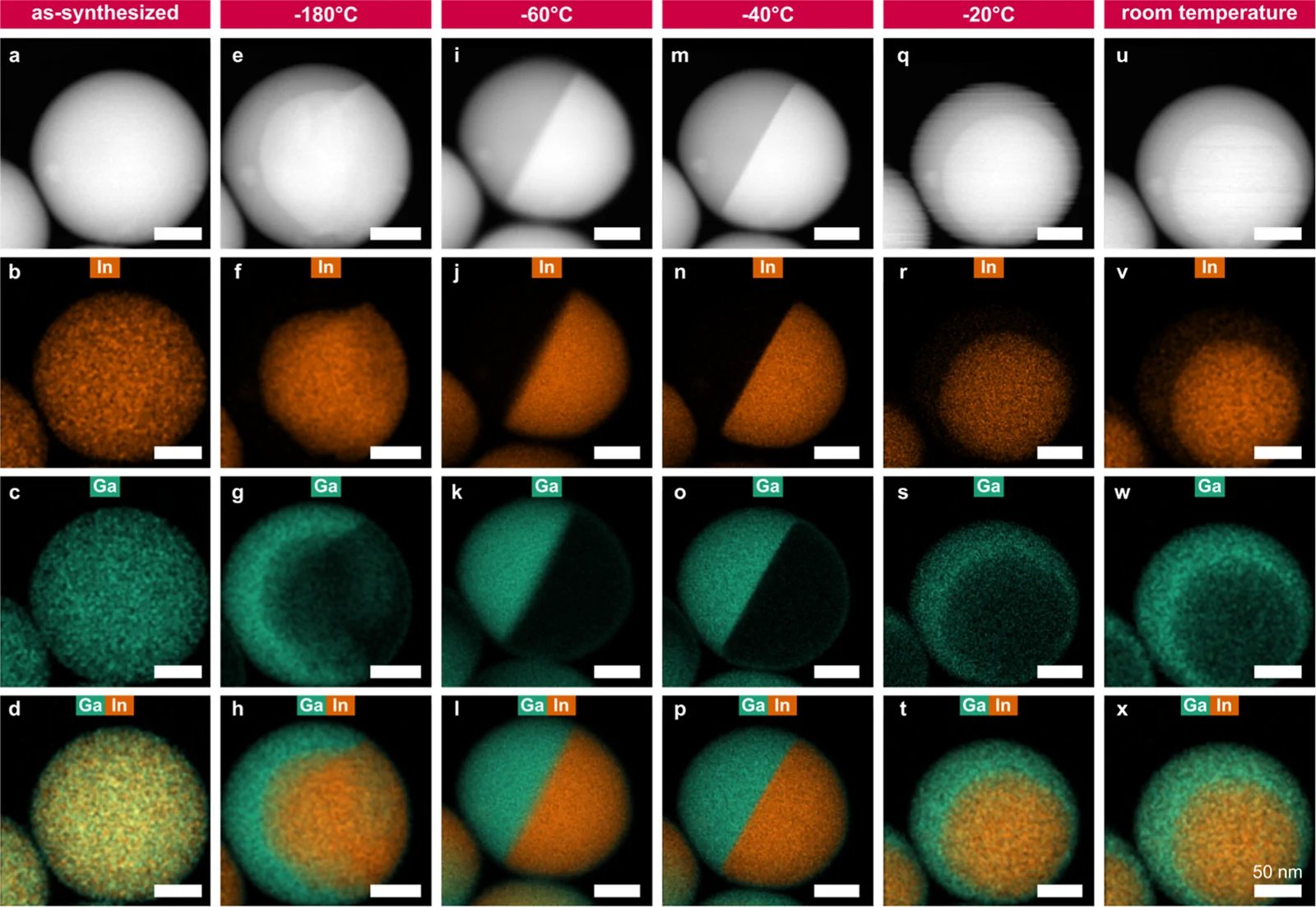
Investigation of the temperature-dependent phase transitions of **M-InGa** NPs HAADF-STEM images and corresponding EDXS elemental maps (orange: indium, green, gallium) of **M-InGa** during in situ microscopy experiment, wherein the NPs are cooled down at the indicated temperature and imaged. (a–d) As-synthesized NP. (e–x) Same NP after being cooled to −180 °C (panels e–h) and heated back to −60 °C (panels i–l), −40 °C (panels m–p), −20 °C (panels q–t), and room-temperature (panels u–x). These data indicate the metastable nature of the as-synthesized In_40_Ga_60_.

These data agree well with one previous study investigating temperature-dependent structural transitions of In–Ga NPs, wherein the preservation of the solid–liquid core–shell structure at room temperature of In_21_Ga_79_ NPs compared to the return to fully liquid of In_83_Ga_17_ was explained by the decrease of the total Gibbs free energy of the solid–liquid core–shell compared to the homogeneous liquid structure with increasing indium content.^29^

### Temperature-Dependent CO_2_ Electroreduction

Having assessed the temperature-dependent behavior of **M-InGa**, we proceeded in testing the as-synthesized In–Ga NPs for CO_2_RR, thus under applied voltage, and at different temperatures (Figures 3 and S22–S25). We prepared the cathode electrode by drop-casting the as-synthesized In–Ga NP ink on glassy carbon and adjusted catalyst loading to maintain a constant total metal (gallium + indium) mass. The similar NP size and same deposited mass enable meaningful relative comparison among the catalyst performance. We conducted the electrocatalytic studies in a custom-made H-cell equipped with a Peltier element heating the working electrode (Figure S22). Indium is a formate-producing CO_2_RR catalyst, whereas gallium generates a modest CO amount while being more active for the hydrogen evolution reaction (HER).^30,31^ The only two previous studies on colloidal LM NPs for CO_2_RR have demonstrated the use of colloidal gallium and silver–gallium NPs to generate CO.^14,15^ While limited in CO_2_RR product scope, these studies have evidenced that the oxide skin surrounding these LM NPs partially remains during CO_2_RR, which enables Ga NP stability along with accessibility to their inner metal (i.e., silver in the silver–gallium NPs).^14,15,32^

**Figure 3:**
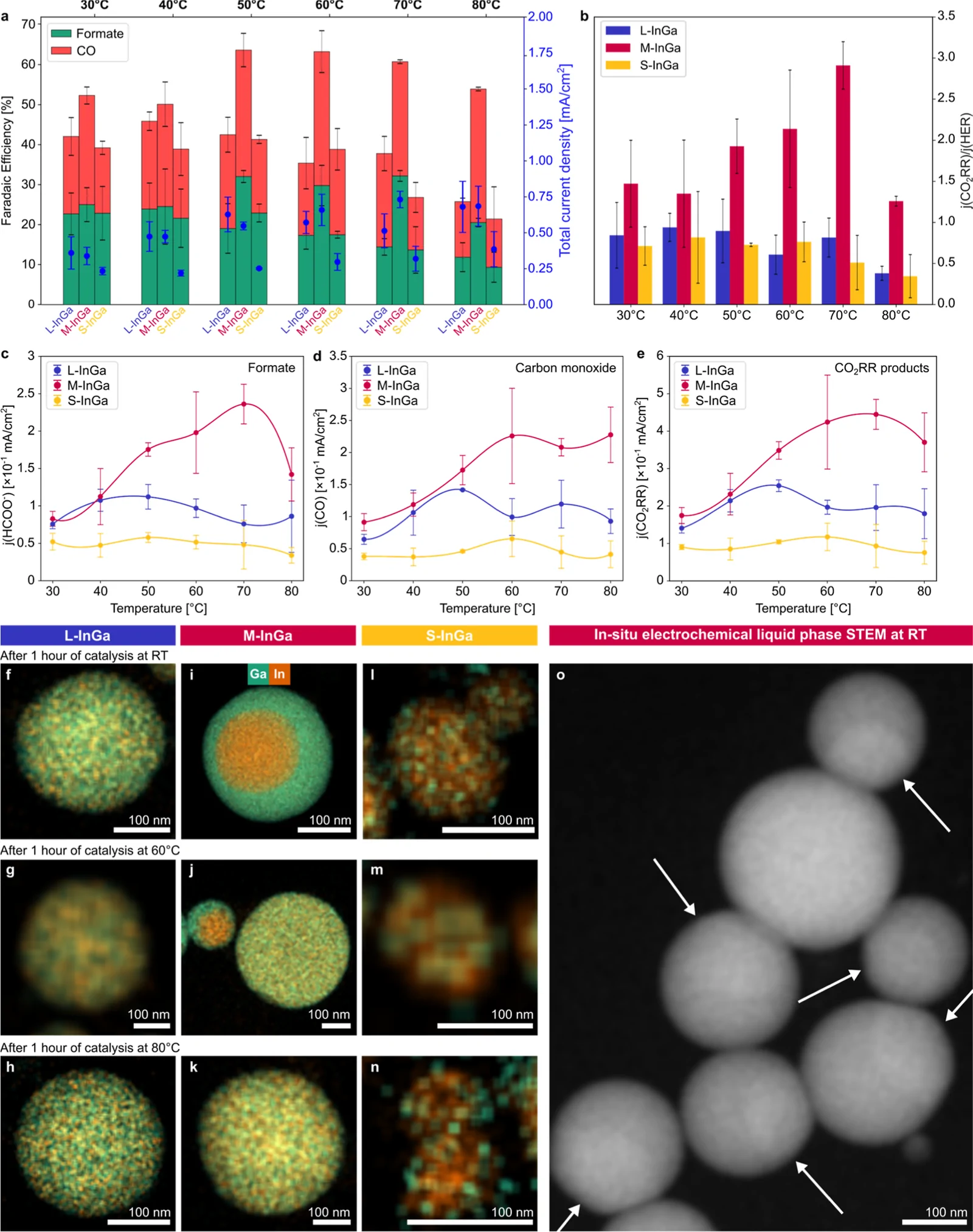
Temperature-dependent CO_2_RR performance along with NP characterization post-catalysis and under CO_2_RR conditions. (a) CO_2_RR Faradaic efficiencies (FEs) and total current density at increasing temperatures for **L-**, **M-**, and **S-InGa**. (b) Corresponding ratios of CO_2_RR to HER currents. (c–e) Corresponding partial current density for formate (panel c), carbon monoxide (panel d), and the CO_2_RR products (panel e). (f–h) Post-catalysis EDXS elemental maps (orange: indium, green: gallium) of **L-InGa** after catalysis at room temperature (panel f), 60 °C (panel g), and 80 °C (panel h). (i,j) Post-catalysis EDXS maps (orange: indium, green: gallium) of **M-InGa** after catalysis at room temperature (panel i), 60 °C (panel j), and 80 °C (panel k). (l–n) Post-catalysis EDXS maps (orange: indium, green: gallium) of **L-InGa** after catalysis at room temperature (panel l), 60 °C (panel m), and 80 °C (panel n). (o) HAADFT-STEM image of **M-InGa** under CO_2_RR conditions in the ecLPTEM cell. The experiments in panels a–n were performed in a H-cell with a KHCO_3_ electrolyte at −0.7 V_RHE_ for 1 h. The data in panels a,b are the average of three independent experiments and the error bars are the calculated standard deviations. For panels c–e, the lines are a guide to the eye.

We chose a voltage of −0.7 V_RHE_ for the CO_2_RR tests, based on these previous studies on LM NPs.^14,15^ The CO_2_RR product distribution switches from only CO being produced with Ga NPs to a mix of CO and formate with all the In–Ga NPs (Figures 3a, S23, and 24), which is consistent with what expected based on the behavior of indium and gallium bulk. However, an interesting trend is observed in the In–Ga NP samples across the entire temperature range. Indeed, **M-InGa** (In_40_Ga_60_) possesses the highest CO_2_RR selectivity and activity, including both CO and formate, among all samples (Figure 3a–e). **L-InGa** (In_17_Ga_83_) follows, and **S-InGa** (In_98_Ga_2_) has the lowest CO_2_RR selectivity and activity (Figure 3a–e).

In addition, **M-InGa** exhibits the highest sensitivity to temperature in both CO_2_RR selectivity and activity, reaching a maximum at 70 °C (Figure 3a–e). Minor changes in CO_2_RR selectivity and activity with temperature occur also for **L-InGa**, while the changes are negligible for **S-InGa** compared to the other samples (Figure 3a–e).

Recent studies indicate that temperature affects CO_2_RR performance by altering CO coverage, local pH, reaction kinetics and pathways, surface atomic structure, and CO_2_ solubility.^33–37^ However, these factors alone are not sufficient to explain the differences among the In–Ga samples which would be similarly impacted by them.

Intrigued by these results, we performed electron microscopy characterization of the In–Ga NPs post-catalysis and under CO_2_RR conditions (Figure 3f–p).

The post-catalysis HAADF-STEM indicates no substantial change in **S-** and **L-InGa** after 1 h at all temperatures (Figure 3f–h,l–n). X-ray photoelectron spectroscopy (XPS) evidence a comparatively similar post-catalysis indium surface enrichment in all catalysts (Figures S26–S28), which is consistent with surface energy considerations.^16^ The **M-InGa** exhibits unique structural and compositional changes. The **M-InGa** are in their (solid indium + gallium-rich liquid) core–shell structure after 1 h of CO_2_RR at room temperature (Figure 3i–k and S29–S34). Additional potential-dependent electrochemical characterization (linear sweep voltammetry) coupled with electron microscopy evidence the simultaneous occurrence of the native oxide reduction and of the formation of the core–shell structure for **M-InGa** in a potential range between −0.6 V_RHE_ and −0.7 V_RHE_ (Figures S35–S37).The simultaneous occurance of this two events might imply that the reduction of the oxide triggers the structural transition.^38^ The potential range for reduction of native oxide is consistent with the previous literature on Ga NPs.^14,32^

In situ electrochemical liquid-phase transmission electron microscopy (ecLPTEM) provides confidence on the ex situ observations. Indeed, ecLPTEM confirms that the catalytically active structure of the **M-InGa** NPs at room temperature is the (solid indium + gallium-rich liquid) core–shell structure (Figure 3o, S38, and S39). The distinct indium-rich core is visible as a brighter domain in the image due to its higher mass compared to the gallium in the liquid phase (Figure 3o). One interesting observation is the asymmetry of the solid indium core, which might result from restructuring to minimize the total Gibbs free energy.^26^

These observations prompted the hypothesis that the solid indium core dissolves as the temperature increases and the NPs progressively become fully liquid with homogeneously dispersed indium into the gallium matrix.

### In Situ Temperature-Dependent Characterization of the Catalytically Active M-InGa

To gain mechanistic information, we performed temperature-dependent in situ characterization of the catalytically active core–shell structure of **M-InGa** (Figure 4, Note S3, and S40–S52).

**Figure 4:**
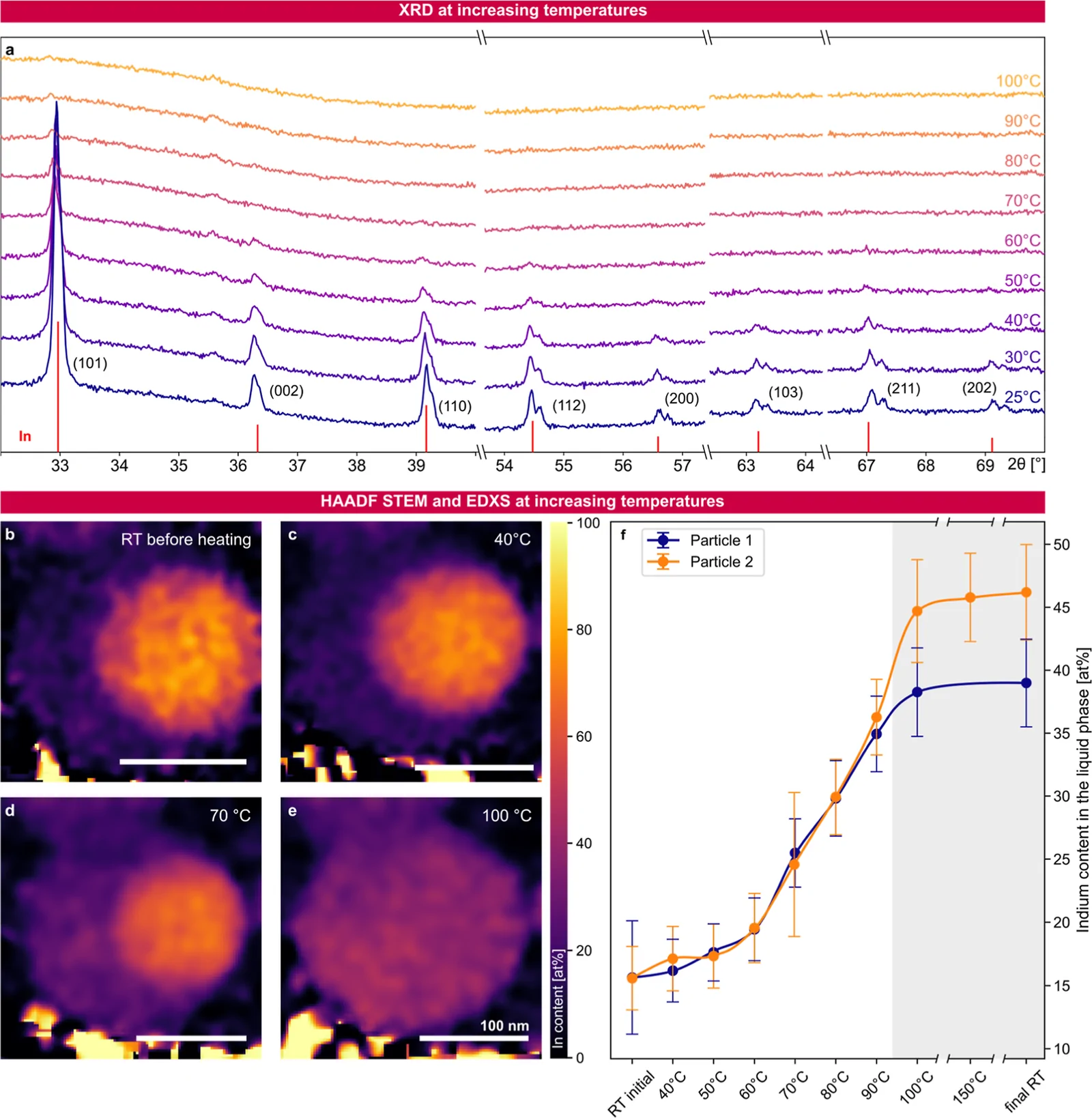
Temperature-dependent structural behavior of the catalytically active core-shell structure of M-InGa. (a) XRD patterns collected while heating **M-InGa** from room temperature to 260 °C. The NPs were immersed in liquid nitrogen prior to the measurement and left to return to room temperature in order to start with the phase-segregated structure. Peak splitting is due to the Cu Kα1/Kα2 doublet of the anode’s emission profile (confirmed by Rietveld refinement in Figure S40). (b–e) STEM–EDXS indium signal maps collected at different temperatures: room temperature (panel b), heated to 40 °C (panel c), further heated to 70 °C (panel d), and heated to 100 °C (panel e). (f) Evolution of the solubilized indium (at %) within the liquid gallium-rich phase at different temperatures for two particles of slightly different average compositions (particle 1:39 at % In, particle 2:46 at % In). The lines are a guide to the eye. Particle 1 is the one represented in panels (b–e).

The XRD pattern displays reflections characteristic of crystalline indium at room temperature, consistent with the fact that the crystalline core is pure indium or indium doped with a small amount of gallium, in agreement with the In–Ga phase diagram (Figures 4a and S40). The indium peak intensity decreases and the peak width increases with temperature consistently with the dissolution of the solid indium-rich core into the gallium matrix (Figures 4a and S41).

The in situ STEM with EDXS mapping confirms the dissolution of indium core and, more specifically, indicates that the indium content in the liquid Ga matrix continuously increases with temperature (Figure 4b,c and S42–S44). This increase is qualitatively visible in the nanoscale chemical maps (Figure 4b–e) where a decrease in the core size is accompanied by an increased intensity of the indium signal in the gallium liquid domain at increasing temperatures (Figure 4b–d). In addition, analysis of the EDXS data through a multiscale Bayesian approach provides quantitative information (Figures 4f, S42–S52, and Note S3).^39^ The results obtained for two distinct particles indicate that (1) around 17 at % of indium is dissolved in the liquid gallium matrix in the catalytically active core–shell structure of **M-InGa** at room temperature, (2) the indium content dissolved in the liquid gallium matrix progressively increases and reaches 40–45 at. % at 100 °C (i.e., when the indium core is fully dissolved), and (3) slightly different overall compositions (39 at % indium in particle 1 and 46 at % in particle 2) do not affect the concentration of indium in liquid gallium at a given temperature. Notably, the amount of dissolved indium in the catalytically active core–shell structure of **M-InGa** at room temperature is similar to that in **L-InGa**.

Although these electron microscopy experiments were performed in vacuum to gain resolution for the analysis, the observed trend is consistent with that obtained from the in situ XRD experiment (Figure S41). We note that the two techniques yield slightly different onset temperatures for dissolution, which could be attributed to different environmental conditions along with the *r* ^3^ dependency of the crystalline volume in XRD vs the *r* ^2^ dependency of the projected area in the STEM images (Figures S53 and 54, Note S4).

Interestingly, both in situ XRD and in situ STEM show that once the core is fully dissolved at 100 °C, the homogeneous liquid structure persists in a supercooled metastable state when returning to room temperature (Figure S41), explaining the synthesis results.

## Discussion

At this point, an overall picture shapes up (Figure 5). The colloidal synthesis generates In–Ga NPs with tunable composition and physical state (i.e., **S-InGa** with In_98_Ga_2_, **M-InGa** with In_40_Ga_60_, **L-InGa** with In_17_Ga_83_). The as-synthesized **M-InGa** exists in a supercooled metastable, fully liquid state. A native oxide skin surrounded these NPs. This oxide shell is typical of gallium-based LM NPs.^14,15,24,32,40^

**Figure 5:**
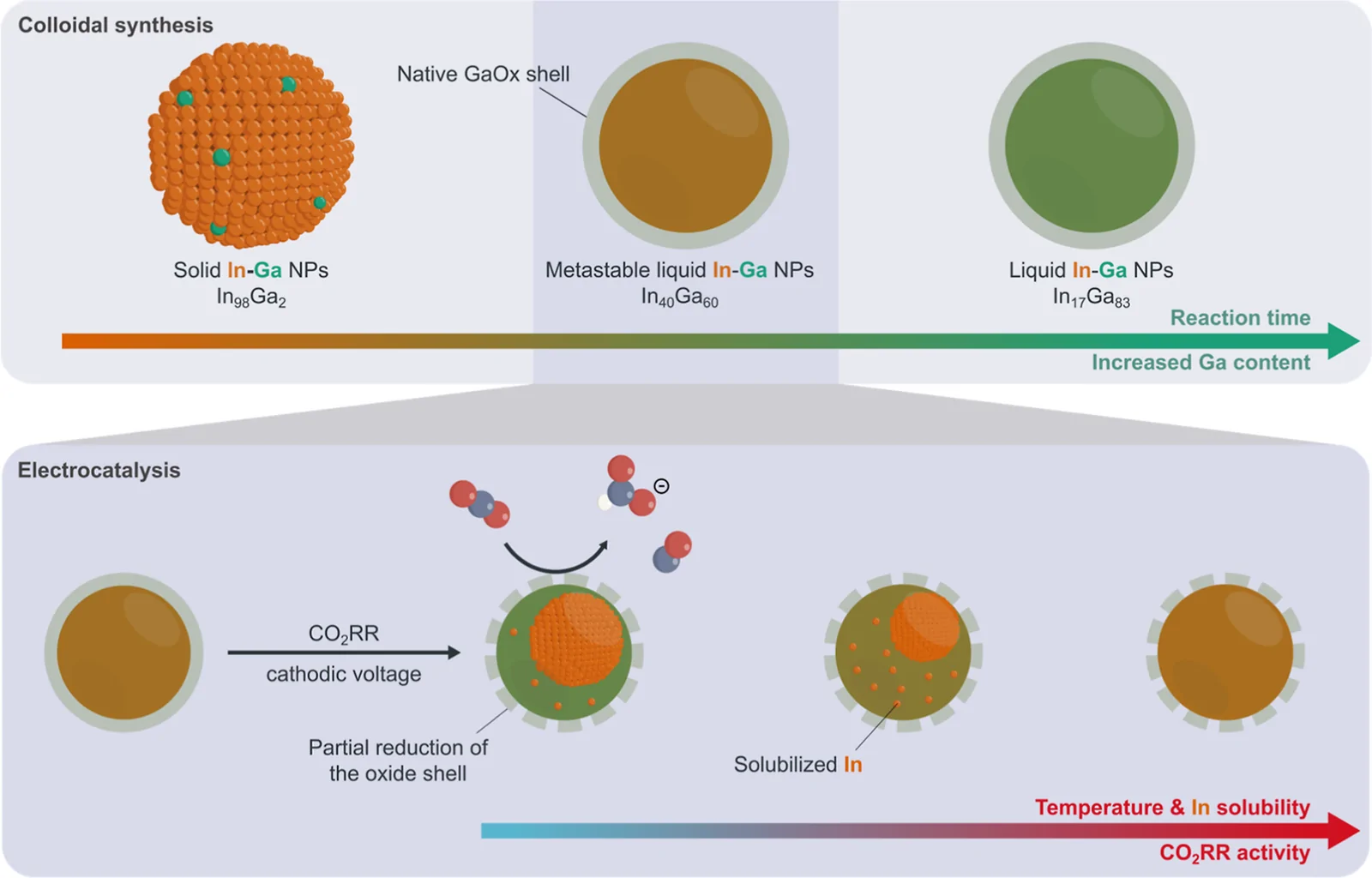
Colloidal In–Ga NPs and their behavior as temperature-tunable CO_2_RR electrocatalysts. A synthetic scheme based on balanced indium and gallium precursor reactivity yields well-defined and tunable In–Ga NPs. Specifically, the synthesis generates a metastable composition, which exhibits a unique behavior as a CO_2_RR electrocatalyst. The application of the cathodic potential partially removes the native oxide skin along with inducing the formation of a phase-segregated core–shell structure containing a solid indium core and indium sites dispersed in the gallium matrix. Upon temperature increase, the indium core dissolves and releases more indium active sites promoting CO_2_RR activity and selectivity. The comparison with the solid and liquid In–Ga NPs highlights the unique reactivity of these indium sites dispersed in the liquid gallium matrix.

The metastable **M-InGa** NPs undergo phase segregation upon cathodic voltage perturbation to form In–Ga core–shell NPs, consisting of a solid indium core encapsulated in a liquid gallium-rich droplet containing around 20 at. % of dissolved indium species. The phase segregation occurs concomitantly with the reduction of the native oxide skin. Previous studies indicate that the oxide skin of gallium-based LM NPs is reduced during CO_2_RR but only partially, which hinders coalescence of the LM NPs into a thin film while still making the active sites in the liquid phase accessible for catalysis.^14,15,32^

The presence of dissolved indium species in the gallium matrix explains the similar CO_2_RR performances of **M-InGa** and **L-InGa** at room temperature. The fact that their CO_2_RR selectivity and activity is superior to that of **S-InGa** with higher indium content hints at a higher intrinsic reactivity of the indium sites when dispersed in liquid gallium, under the assumption that the number of surface active sites reflects the sample bulk composition (Note S5 and Figure S55). The solid indium domain in the catalytically active **M-InGa** core–shell structure acts as a reservoir releasing the catalytic indium active sites as the temperature increases.

CO_2_RR formate selectivity in melted Ga–In and Ga–Sn bulk drops has been attributed to atomically dispersed In and Sn species via in situ XAS.^8^ Metadynamics simulations have indicated a strong preference for CO_2_RR to occur at indium dopant sites dispersed in the gallium matrix along with suggesting a role of the liquid gallium surface in enabling proton surface mobility possibly contributing to the reaction outcome.^41^ These previous studies suggest that indium active sites in **M-InGa** and **L-InGa** are indeed isolated indium atomic sites. However, future work using surface-sensitive techniques (e.g., in situ Raman or in situ FTIR) should be considered to specifically identify surface-active sites where interactions between indium and gallium oxide or other indium species (e.g., clusters, oxidic indium) might exist and play a role.

Overall, the discovery of this work aligns with and strengthens the existing literature on the unique behavior and opportunities offered by supported catalytically active liquid metal solutions.^10,42–45^

In the context of CO_2_RR, catalysts preserving and/or improving their activity with temperature are emerging as important in the field due to their enhanced compatibility with industrial reaction conditions as well as fundamental interest.^30–34^ Two previous studies on solid catalysts reported CO_2_RR deactivation with increasing temperature due to structural changes, characterized in particular by decreased formate production and selectivity.^34,37^ In contrast, the formate production rate in **M-InGa** increases with temperature, highlighting their promise as catalysts which do not deactivate with temperature. Future studies are needed to explain the optimal CO_2_RR performance temperature, which must result from a balance between generating more indium active sites and changes in the CO_2_RR environment (CO_2_ solubility, CO coverage, pH changes, etc.) with increasing temperature.

## Conclusion

In conclusion, we propose the synthesis of colloidal In–Ga NPs with uniform and tunable morphology and composition, which enables access to a metastable In–Ga phase. The NPs in this metastable phase offer a temperature-based approach to modulate indium solubility in Ga NPs. Using this concept, we demonstrated temperature-dependent tunability of the indium active site concentration in the gallium matrix during CO_2_RR, which results in increasing activity and selectivity toward CO_2_RR, particularly CO and formate. Controlling temperature to tune the concentration of active sites emerges as an additional design parameter for CO_2_RR catalysts, especially important considering high-temperature applications, and paves the way for exploiting the unique physicochemical properties of liquid metal nanoparticles in catalysis.

## Methods

### Synthesis of In–Ga Nanoparticles (S-, M-, and L-InGa)

In a typical synthesis, 7 mL of ODE was loaded into a 25 mL three-necked flask, equipped with a reflux condenser, and dried under vacuum at 110 °C for 1 h. Then, the reaction flask was filled with N_2_ and heated to 290 °C, followed by the swift injection of a solution containing InBr_3_, TDMAG, dried DOA, and dried ODE using a syringe. The reaction temperature was then set to 260 °C and the solution was left to stir for various durations, depending on the target composition. The reaction medium was then transferred to the glovebox and In–Ga NPs were separated from byproducts and unreacted precursors by adding 10 mL of EtOH, followed by centrifugation at 4400 rpm (3000 rcf) for 5 min. In–Ga NPs were redispersed in toluene and the purification/precipitation steps were repeated 2–3 times. Finally, the In–Ga NPs were stored as a suspension in toluene.

### Electrocatalytic Measurements

The catalyst ink was prepared by transferring the volume of NPs stored in toluene corresponding to the desired mass loading into a 1.5 mL Eppendorf tube and centrifuging it. Upon removing of the supernatant, 40 μL of a carbon black/Nafion suspension was added and the resulting ink drop-casted on a 1.33 cm^2^ glassy carbon substrate. More information on the ink preparation is provided in Supporting Information.

Electrochemical measurements were performed in a custom-made H-cell using 0.1 mol L^–1^ KHCO_3_ as the electrolyte, a CO_2_ flow rate of 5 mL min^–1^, Ag/AgCl as the reference electrode, and platinum foil as the counter electrode. The catholyte and anolyte compartments were separated by a Selemion AMV membrane. The working electrode temperature was controlled with a custom Peltier-based heating and cooling module. Additional information on the electrochemical measurements can be found in the Supporting Information.

## Supplementary Material



## Data Availability

Data Availability Experimental data are openly available in Zenodo at: 10.5281/zenodo.21716735.

## Notes

Additional experimental details, materials, and methods; catalyst characterization; additional HAADF-STEM images and EDXS maps; and cryo-TEM images, XPS data, CO_2_RR Faradaic efficiency plots, XRD, schematics of the ecLPTEM set up and electrochemical cell with integrated Peltier element, and additional information on indium quantification with EDXS (PDF)
