# Supplementary material for: Colloidal Synthesis Enables Indium Solubility Modulation in Liquid Gallium Nanoparticles for Temperature-Dependent Generation of CO2 Electrocatalytic Active Sites
Source: J Am Chem Soc. 2026 Aug 7;148(32):35031–40. doi: 10.1021/jacs.6c11963 (PMC13495752; doi:10.1021/jacs.6c11963)
Supplement: Supplementary file 1 [file ja6c11963_si_001.pdf]

Supplementary Information

Colloidal Synthesis Enables Indium Solubility Modulation in Liquid Gallium Nanoparticles for Temperature-dependent Generation of CO_2 Electrocatalytic Active Sites

Coline Boulanger[1], Pau Torruella[2], Krishna Kumar[1], Pascal Schouwink[3], Petru P. Albertini[1], Anna Loiudice[1], Raffaella Buonsanti[1]*

[1]Laboratory of Nanochemistry for Energy (LNCE), Institute of Chemical Sciences and Engineering (ISIC), École Polytechnique Fédérale de Lausanne, CH-1950 Sion, Switzerland.

[2]Electron spectrometry and microscopy laboratory (LSME), Institute of Physics (IPHYS), École Polytechnique Fédérale de Lausanne, CH-1015 Lausanne, Switzerland.

[3]Institute of Chemical Sciences and Engineering (ISIC), École Polytechnique Fédérale de Lausanne, CH-1950 Sion, Switzerland.

*Corresponding author

Table of Figures

Figure S1– Synthesis of In-Ga NPs with tunable composition. 9
Figure S2 – Size distribution of the as-synthesized NPs. 10
Figure S3 – Comparison of the reaction medium color evolution with and without the gallium precursor.. 11
Figure S4 – In-Ga NPs obtained by sequential injection of indium and gallium precursors................................ 12
Figure S5 – Indium particles formation through *in situ* generation of indium long-amide species. 13
Figure S6 – Proposed reaction mechanisms for the formation of the In-Ga NPs.. 14
Figure S7 – HAADF STEM and EDXS of as-synthesized S-InGa.. 15
Figure S8 – HAADF STEM and EDXS of as-synthesized M-InGa. 16
Figure S9 – HAADF STEM and EDXS of as-synthesized L-InGa.. 17
Figure S10 – TEM images and corresponding SAED pattern for S-InGa, M-InGa and L-InGa 18
Figure S11 – XRD pattern of as-synthesized S-InGa.. 19
Figure S12 – In-Ga binary phase diagram. Adapted from ref.[7] 20
Figure S13 – Phase segregation of the metastable In-Ga NPs when immersed in liquid N_2................................ 22
Figure S14 – HAADF STEM and EDXS of M-InGa after triggering its phase segregation................................ 23
Figure S15 – Moving cores within M-InGa NPs after immersion in liquid nitrogen.. 24
Figure S16 – HAADF-STEM images M-InGa at room temperature after immersion in liquid nitrogen. 25
Figure S17– XRD patterns collected while heating S-InGa from room temperature to 160°C. 26
Figure S18 – Additional cryo-STEM imaging on a M-InGa NP. 27
Figure S19 – Additional cryo-STEM imaging on two M-InGa NPs.. 28
Figure S20 – Cryo-TEM imaging and diffraction patterns of L-InGa.. 29
Figure S21 – Phase identification in L-InGa at -60°C. 30
Figure S22 – Electrochemical cell with integrated Peltier-element. 31
Figure S23 – Effect on temperature on CO_2RR Faradaic efficiency and total current density at -0.7 V_{RHE}. 32
Figure S24 – CO_2RR Faradaic efficiency and total current density at -0.7 V_{RHE}. 33
Figure S25 – CO_2RR Faradaic efficiency and total current density at -1.1 V_{RHE}. 34
Figure S26 – Surface indium enrichment evidenced by XPS.. 35
Figure S27 – XPS spectra of the Ga2p region of In-Ga NPs before and after CO2RR at room-temperature. 36
Figure S28 – XPS spectra of the In3d region of In-Ga NPs before and after CO2RR at room-temperature. 37
Figure S29 – HAADF STEM and EDXS of S-InGa after 1h at -0.7 V_{RHE}.. 38
Figure S30 – HAADF STEM and EDXS of L-InGa after 1h at -0.7 V_{RHE}.. 39
Figure S31 – HAADF STEM and EDXS of M-InGa after 1h at -0.7 V_{RHE}. 40
Figure S32 – HAADF-STEM images of S-InGa after 1h of catalysis at different temperature........................... 41
Figure S33 – HAADF-STEM images of M-InGa after 1h of catalysis at different temperature.......................... 41
Figure S34 – HAADF-STEM images of L-InGa after 1h of catalysis at different temperature........................... 41
Figure S35 – HAADF-STEM images and EDXS of S-InGa post-LSV. 42
Figure S36 – HAADF-STEM images and EDXS of M-InGa post-LSV.. 42
Figure S37 – HAADF-STEM images and EDXS of S-InGa post-LSV.. 43
Figure S38 – Schematic of the components of the ecLPTEM set up. 43
Figure S39 – HAADFT-STEM image of M-InGa during catalysis in the ecLPTEM cell. 44
Figure S40 – XRD pattern of M-InGa at room temperature after immersion in liquid nitrogen.......................... 45
Figure S41 – XRD patterns collected while heating M-InGa from room temperature to 260°C. 45
Figure S42 – Gallium and indium elemental EDXS maps at increasing temperatures for Particle 1.................... 47
Figure S43 – Gallium and indium elemental EDXS maps at increasing temperatures for Particle 2.................... 48
Figure S44 – Gallium and indium elemental EDXS maps at increasing temperatures for Particles 3. 49
Figure S45 – HAADF-STEM images and elemental maps (green: Ga, red: In) showing the selected area for quantifying indium in the liquid phase of Particle 1 at temperatures where the core remains rather stationary. .. 50
Figure S46 – HAADF-STEM images and elemental maps (green: Ga, red: In) showing the selected area for quantifying indium in the liquid phase of Particle 2 at temperatures where the core remains rather stationary. .. 51
Figure S47 – Pixels selection for the indium quantification in the liquid phase at 80°C for Particle 1, where the core was continuously moving... 52
Figure S48 – Pixels selection for the indium quantification in the liquid phase at 90°C for Particle 1, where the core was continuously moving... 53
Figure S49 – Pixels selection for the indium quantification in the liquid phase at 80°C for Particle 2, where the core was continuously moving... 54

Figure S50 – Pixels selection for the indium quantification in the liquid phase at 90°C for Particle 2, where the core was continuously moving.. 55
Figure S51 – Reversibility of the dissolution process.. 56
Figure S52 – Core dissolution. HAADF-STEM images (a) at room temperature before the beginning of the heating experiment (b) at 90°C.. 57
Figure S53 – Testing of the two-spheres model for predicting the core size of In-Ga NPs. 61
Figure S54 – Effect of a core size reduction on the indium content of the liquid phase for M-InGa. 62
Figure S55 – Normalized formate partial current densities. 65

Table of Notes

Note S1– Insights into the formation mechanism. 9
Note S2 – Supercooling and size effect. 21
Note S3 – Indium quantification in the liquid phase at increasing temperature through electron microscopy 46
Note S4 – Simplified model to predict M-InGa structure and composition at different temperatures 58
Note S5 – Discussion on the intrinsic activity and active site density of M-InGa. 63

Synthetic Methods

Materials

Tris(dimethylamido)gallium(III) ($Ga_2(NMe_2)_6$, TDMAG, 98%) was bought from American Element. Indium(III) bromide ($InBr_3$, 99.999%) was purchased from Carl Roth GmbH + Co. KG. Di-*n*-octylamine (DOA, 98%), octadecene (ODE, technical grade 90%), carbon black (Sigma Aldrich < 500 nm), Nafion (5 wt% in lower aliphatic alcohols and water, contains 15-20% water), n-Butyllithium solution (2.7 M in heptane), Super-Hydride® solution (1.0 M lithium triethylborohydride in THF) and toluene (anhydrous, 99.8%) were bought from Sigma-Aldrich. Ethanol (dried, max 0.01% H_2O) was purchased from Merck.

Methods

General considerations

All nanoparticle syntheses and manipulations were conducted under an inert, dry N_2 atmosphere in a glovebox or with Schlenk-line methods. Anhydrous or dried solvents were used throughout synthesis, storage, and purification, and glassware was maintained at 110 °C to minimize moisture exposure.

Synthesis of In-Ga nanoparticles (S-, M- or L-InGa NPs)

In a typical synthesis, 7 mL of ODE were loaded into a 25 mL three-necked flask, equipped with a reflux condenser, and dried under vacuum at 110 °C for 1 h. Then the reaction flask was filled with N_2 and heated to 290 °C, followed by the swift injection of a solution containing 17 mg (0.048 mmol) of $InBr_3$, 50 mg of $Ga_2(NMe_2)_6$ (0.124 mmol), 2.26 mL of dried DOA and 2 mL of dried ODE using a syringe. This light-yellow solution was previously prepared by stirring the In and Ga precursors in ODE and DOA inside the glove-box for at least 30 min. The hot injection let to a temperature drop to *ca.* 260°C. Following the injection, the reaction temperature was then set to 260 °C and the solution was left to stir for various duration, depending on the target composition. The reaction medium was then transferred to the glove-box and In-Ga NPs were separated from by-products and unreacted precursors by adding 10 mL of ethanol followed by centrifugation at 4400 rpm (3000 rcf) for 5 min. In-Ga NPs were redispersed in toluene and the purification/precipitation steps were repeated 2-3 times. Finally, the In-Ga NPs were stored as a suspension in toluene.

Synthesis of Ga nanoparticles (Ga NPs)

The synthesis of Ga NPs was adapted from a previously reported procedure.[1] In a typical synthesis, 7 mL of ODE were loaded into a 25 mL three-necked flask, equipped with a reflux condenser, and dried under vacuum at 110 °C for 1 h. Then the reaction flask was filled with N_2 and heated to 290 °C, followed by the swift injection of a solution containing 50 mg of $Ga_2(NMe_2)_6$ (0.124 mmol), 2.26 mL of dried DOA and 2 mL of dried ODE using a syringe. This yellow solution was previously prepared by stirring the Ga precursor in ODE at 60°C and adding the DOA *ca.* 10 min before the addition. The reaction was then quickly cooled down to RT using an ice bath. The reaction medium was then transferred to the glove-box and Ga NPs were separated from by-products and unreacted precursor following the same post-synthetic procedure as for the In-Ga NPs.

Instrumentation

X-ray diffraction (XRD) patterns were recorded on a Bruker D8 discover instrument equipped with a LynxEye XE detector (Cu radiation). The instrument was operated at 40 mA and 40 kV. Samples were prepared by dropcasting the NPs suspension in toluene on a VWR® microscope slide. To probe the thermodynamically stable phase of the In-Ga nanoparticles, the slide was briefly immersed in liquid nitrogen and then allowed to return to room temperature prior to the XRD measurement. For the temperature-dependent XRD measurements, a homemade high temperature cell was used. The cell consists of a heating element which hold the slide with the deposited nanoparticles. A thermometer measures the surface of the substrate. The heating element and the substrate are pressed against two side bars at a given height using spring in order to keep the substrate height constant. These springs are important to accommodate the substrate expansion with temperature. Yet, these springs might become loose with time, which results into shifts in the XRD peaks, such as those observed **Figure S17**, due to substrate expansion and minor height changes. The slide was heated from room temperature to 260 °C, with isothermal plateaus at selected temperatures during which spectra were recorded. PDF-5+ 2024 was used to find all the cif files for XRD peak attribution.

High-angle annular dark-field scanning transmission electron microscopy (HAADF-STEM) images and EDXS elemental maps were acquired on a probe-corrected Thermo Fisher Scientific Spectra 200 (S)TEM operated at 200 kV. This microscope is equipped with a high brightness X-CFEG, Super-X EDXS acquisition system and Velox acquisition software. The NP suspensions were drop-cast on Cu grid.

Cryo-electron microscopy measurements were performed in a Talos F200s FEI TEM operated at 200 kV. The sample holder used was a Gatan 626 single tilt Cryo-holder. Each specimen was drop-cast onto a holey carbon TEM grid. For each experiment, the sample was first cooled down to liquid nitrogen temperature and then heated at different temperatures (-180 °C up to 80°C) in 20°C steps and left to stabilize for a minimum of 10 minutes. At each temperature, STEM-HAADF, EDX and selected area electron diffraction (SAED) measurements were acquired.

In-situ heating electron microscopy measurements were performed in a probe-corrected Thermo Fisher Scientific Spectra 200, operated at 200 kV. A Protochips Fusion heating sample holder was used to heat the sample from room temperature up to 150°C while acquiring STEM-

HAADF images and EDX maps at different temperatures. The temperature was increased in steps of 10°C.

Prior to the experiment the sample had been cooled in liquid nitrogen and then left to stabilize at room temperature, a process that induced the phase segregation of the NPs into In-rich cores and Ga-rich liquid phase.

The acquired EDXS maps were quantified using a robust multiscale bayesian (RMB) approach.[2] This approach had several advantages: First it allowed obtaining denoised, quantified elemental maps, from the relatively low signal-to-noise ratio EDXS measurements (acquisition times of only 1-2 minutes were used to avoid beam damage and keep the experiment duration reasonable). Secondly, it allowed obtaining a good estimation of the experimental uncertainty of the quantification through bayesian statistics. Lastly, it allowed to automatize the quantification for all temperatures and all sample particles removing human bias.

To specifically quantify the composition of the liquid phase, a computer vision approach was used to segment the particles and indium core separately, using the skimage python library.[3] The liquid phase was taken as the difference between the two. At higher temperatures (80°C and 90°C) the indium cores were moving rapidly from one EDX frame to the next, so the image segmentation was performed frame by frame, summing the signal of all pixels assigned to the liquidus phase and then quantifying the obtained spectrum (see **Note S3**).

Electrochemical liquid phase TEM (ecLPTEM) experiments were performed in a Thermo Fisher Scientific Spectra 200, operated at 200 kV using a Hummingbird Scientific Generation V Bulk Liquid Electrochemistry TEM holder, equipped with a platinum counter electrode and an Ag/AgCl (3 M KCl) reference electrode (**Figure S38**).[4] The tip of the holder serves as the liquid cell, which consists of two chips: a top chip (referred to as the "electrochemistry chip") and a bottom chip, both featuring 50 nm thick silicon nitride membrane windows. The top chip has a thin carbon film covering the patterned working electrode, while the bottom chip includes a 250 nm spacer layer. Both chips were sourced from Hummingbird Scientific. The chips were plasma cleaned for 2 min with an O2 plasma to enhance hydrophilicity. Then, 10 µL of InGa NPs suspended in toluene were drop-casted onto the electrochemistry chip and allowed to dry for 15 min before assembling the liquid cell. The assembled cell was placed in a leak-check station. After confirming that the pressure was below 2.5×10^{-6} Torr, the holder was transferred

to the TEM for further analysis. The electrolyte (0.1 M $KHCO_3$) was prepared by dissolving 138 mg of K_2CO_3 in 20 mL of ultrapure water and then saturated with CO_2 by bubbling the solution for 30 min. The holder is equipped with two microfluidic tubings, through which the liquid can be continuously flowed. The electrolyte (CO_2 saturated 0.1 M $KHCO_3$) was continuously flowed at 2 µL/min using an external syringe pump and a 1 mL glass syringe through one tube (inlet) into the cell. The presence of liquid in the cell was confirmed by liquid flowing through the other tube (outlet) and by the stabilization of an open-circuit potential around the relevant value. The electrochemical potentials were measured with respect to an integrated miniature Ag/AgCl reference electrode within the holder. The Ag/AgCl (3 M KCl) reference electrodes were tested using the electrode test block from Hummingbird Scientific. A Biologic SP-200 potentiostat was used to conduct the electrochemical experiments. The applied potentials were converted to the reversible hydrogen electrode using the following equation:

$$V_{RHE} = V_{Ag/AgCl} + 0.206 + 0.0591 \times pH$$

With V_{RHE} [V] being the potential *vs* RHE and $V_{Ag/AgCl}$ [V] the applied potential *vs* Ag/AgCl (3 M KCl) reference electrode. The pH of the electrolyte used was 6.8. All potentials reported in this work are not IR corrected, as the IR drop is assumed to be negligible.

Inductively coupled plasma optical emission spectroscopy (ICP-OES) was performed on an Agilent 5100 instrument equipped with a VistaChip II CCD detector. The samples were prepared by drying the as-prepared NP suspensions, followed by overnight digestion of the remaining NPs in 142.5 µL of 70 wt.% nitric acid ICP grade solution (Sigma-Aldrich). Milli-Q water was then added to obtain a final solution containing 2 wt.% acid content. Prior to each measurement, ICP-grade standard solutions (1000 ppm in 2 wt.% nitric acid) were diluted with 2 wt.% of nitric acid to prepare fresh solutions to generate the calibration curve used to determine the sample concentrations.

X-ray photoelectron spectroscopy (XPS) measurements were conducted on an Axis Supra (Kratos Analytical) instrument, using the monochromated Kα X-ray line of an Al anode. The pass energy was set to 20 eV with a step size of 0.1 eV. The samples were electrically insulated from the sample holder and charges were compensated. Spectra were referenced at 284.8 eV using the C–C bound of the C 1s orbital. The NPs samples were prepared by drop-casting NP films onto clean Si substrates.

Electrocatalytic CO2RR experiments were conducted in a custom-made gas-tight electrochemical cell made of polycarbonate and fitted with Buna-N O-rings (**Figure S22**). More

precisely, the cell was designed so that the working electrode (WE) is positioned parallel to the counter electrode (CE), ensuring a uniform potential distribution across the surface. The geometric area for both electrodes was 1.33 cm^2. The anodic and cathodic compartments were separated by a Selemion AMV anion exchange membrane and each compartment was filled with 2 mL of 0.1 M $KHCO_3$ electrolyte. This electrolyte was previously prepared by bubbling CO_2 in a 0.05 M K_2CO_3 solution for 1 h. During electrolysis, ambient pressure CO_2 gas was continuously bubbled through the electrolyte at a flow rate of 5 sscm to prevent the depletion of CO_2 in the electrolyte. The gaseous products were continuously analyzed using a gas chromatograph. A Bronkhorst mass flow controller was used to control the flow rate of CO_2, and the gas was humidified with water prior to going into the cell to mitigate the electrolyte evaporation. The CE was a platinum foil and a Ag/AgCl electrode (leak free series) from Innovative Instruments, Inc. was used as the reference electrode (RE). The voltages *vs.* Ag/AgCl were converted into voltages *vs.* RHE by using the following equation:

$$E\ [\text{V}\ vs\ \text{RHE}] = E_{ref} + 0.206 + 0.0591 \times pH - (R_{cell} \times I)$$

with E_{ref} [V] being the recorded potential *vs.* the Ag/AgCl RE, +0.206 V the correction to the Ag/AgCl reference, R_{cell} [Ω] the ohmic resistance between the WE and the RE, determined using electrochemical impedance spectroscopy (EIS) prior to the measurement, and I [A] the imposed current.[5]

The temperature of the WE was controlled using a custom Peltier-based heating/cooling module (**Figure S22**) powered by a 12 V, 150 W power supply. The temperature regulation was achieved using a PID temperature controller connected to a type-K thermocouple placed at the surface of the working electrode, outside of the area delimitated by the O-ring that is covered with the catalyst and immersed in the electrolyte. The controller continuously compares the measured temperature with the setpoint and adjusts the power delivered to the Peltier element (Peltier 16V 9A 30x30x3.6 mm from RS Components GmbH) via pulse-width modulation at 1 kHz. The pulse-width modulation signal (0–5 V) is converted through an H-bridge amplifier, allowing the system to deliver 0 V or 12 V to the Peltier element with adjustable duty cycle. The H-bridge enables polarity inversion, allowing the Peltier element to operate in both heating and cooling modes: one polarity drives heating and the reversed one drives cooling. Visual indicators connected to the Peltier output signal the operating mode (red LED: heating; blue LED: cooling) and their brightness reflects the applied power. A built-in safety system protects the setup. The PID controller includes an alarm output that is triggered in case of over-/under-

temperature or thermocouple failure. This signal controls a relay that can physically disconnect the H-bridge from the power supply, allowing emergency shutdown. The operator can also activate the emergency shutdown via a front-panel switch. The Peltier element functions as a thermoelectric heat pump, transferring heat from one side to the other depending on the applied polarity. Because of intrinsic losses, the hot side must be actively cooled using a fan-assisted heat sink to maintain efficient operation and enable stable temperature control.

All the electrochemical experiments were conducted using a Biologic SP-300 potentiostat. Linear sweep voltammetry (LSV) experiments were performed at a sweep rate of 10 mV/s. Chronoamperometry (CA) experiments were conducted at constant potentials of -0.7 V *vs* RHE and a LSV was always run prior to the CA to have a potential ramp to the target potential.

Gas product quantification during CO_2 reduction experiments was made using an SRI Instruments gas chromatograph (GC) equipped with a HayeSep D porous polymer column, a thermal conductivity detector and a flame ionization detector. The GC was calibrated for H_2, CO, CH_4, C_2H_4 and C_2H_6. The calibration plots for gaseous-product concentration determination were obtained using five standard gas mixtures (Carbagas). The carrier gas was ultra-high purity N_2 (99.999%).

The Faradaic efficiency (FE) for each gaseous product is calculated using the following equation:

$$FE = n_e \times F \times C \times f \times P \times R \times T \times I$$

with n_e being the number of electrons transferred to the product formation, F the Faraday constant (F = 96 485 C.mol^{-1}), C [ppm] the concentration of the product measured by the GC, f [mL.s^{-1}] the gas flow rate, P [Pa] the pressure (P = 1.013×10^5 Pa), R the universal gas constant (R = 8.314 J.mol^{-1}.K^{-1}), T [K] the temperature and I [A] the current. The values obtained after 10 min of catalysis are excluded from the calculation of FE as the GC gas flow is not stabilized at this time. The electrolysis is set to last 3900 s which corresponds to seven injections. For all the reported values, the error bar representing the standard deviations were obtained by doing at least duplicates of each measure.

Liquid product quantification from the CO_2 reduction experiments was made using high-performance liquid chromatography (HPLC) on an UltiMate 3000 instrument from Thermo Scientific, which is equipped with a refractive index detector for product quantification and Aminex HPX-87H (BioRad) column for product separation (1 mM H2SO4 was used as the

eluent). The total volume of both the anolyte and catholyte was prelevated in the same syringe and 0.5 mL of the volume was used for analysis. Collecting electrolyte from both compartments is necessary because products such as formic acid (present as formate under neutral conditions) can migrate through the anion-exchange membrane from the catholyte to the anolyte.

The FE for each liquid product is calculated as follows:

$$FE = n_e \times F \times C \times V \times I \times t$$

With n_e being the number of electrons transferred to the product formation, F the Faraday constant (F = 96 485 $C.mol^{-1}$), C [$mol.L^{-1}$] the concentration of the product measured by the HPLC, V [4 mL] is the electrolyte volume, I [A] the current and t [s] the duration of the electrolysis.

Electrode preparation was done through the drop-casting of 40 µL (with Nafion and CB) of the catalyst suspension on 1.33 cm^2 glassy carbon. To prepare the suspension, the concentration of the NPs stored in toluene was determined through ICP-EOS. The volume corresponding to the targeted loading (120 µg of Ga content. For the In + Ga NPs physical mixture, the metal contents were matching those of In-Ga NPs) was transferred into a 1.5 mL Eppendorf and centrifuged at 14 000 rpm for 10 min. Then, the supernatant was removed, and 40 µL of the CB and Nafion suspension were added. The solution was then sonicated for 1 min andwas left to stir for 30 min and sonicated again for 1 min before drop-casting. The Nafion and carbon black (CB) mixture was prepared by sonicating 2.5 mg of CB with 20 µL of Nafion and 980 µL of THF for 1h. For each electrode preparation, 40 µL of this batch suspension was used.

Note S1– Insights into the formation mechanism.

Figure S1 shows the synthesis procedure. In a typical synthesis, trisdimethylamidogallium (TDMAG) and indium bromide ($InBr_3$) are co-injected into a reaction medium at 290 °C. Depending on the duration *t* of the temperature plateau at 260°C following the injection, In-Ga NPs with different compositions were obtained, ranging from nearly pure In NPs for *t* = 0s to a composition approaching the eutectic point at *t* = 1 hour (**Figure S1b**). The synthesis takes inspiration from a previously developed method for the preparation of Ga NPs containing noble metals.[6]

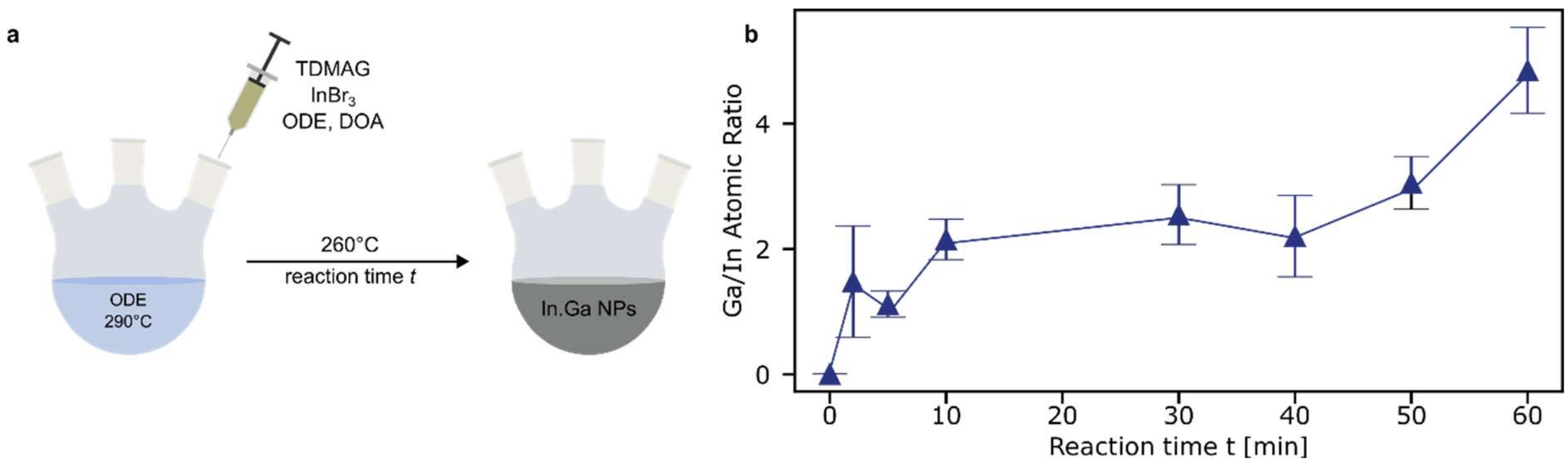

Figure S1– Synthesis of In-Ga NPs with tunable composition. (a) Sketch of the reaction procedure. **(b)** Ga/In average atomic ratio of the synthesized NPs as a function of the reaction time *t*. The atomic ratios were measured by energy dispersive X-ray spectroscopy (EDXS).

Representative HAADF-STEM images and size distribution are shown in **Figure S2**. The progressive gallium enrichment observed with increasing plateau duration *t* suggests that the formation begins with an indium seed, onto which gallium subsequently nucleates. Given that the melting point of bulk indium is 156.6 °C,[7] the seed is expected to be liquid under the reaction conditions, which facilitates the amalgamation of gallium and results in the formation of homogeneous NPs. Similar mechanism has been previously reported for syntheses of solid[8] or biphasic solid-liquid NPs[6] were gallium nucleates at the top of solid seeds. However, in these reported syntheses the metal seeds could be formed in the absence of gallium, while something different is observed here (**Figure S3**).

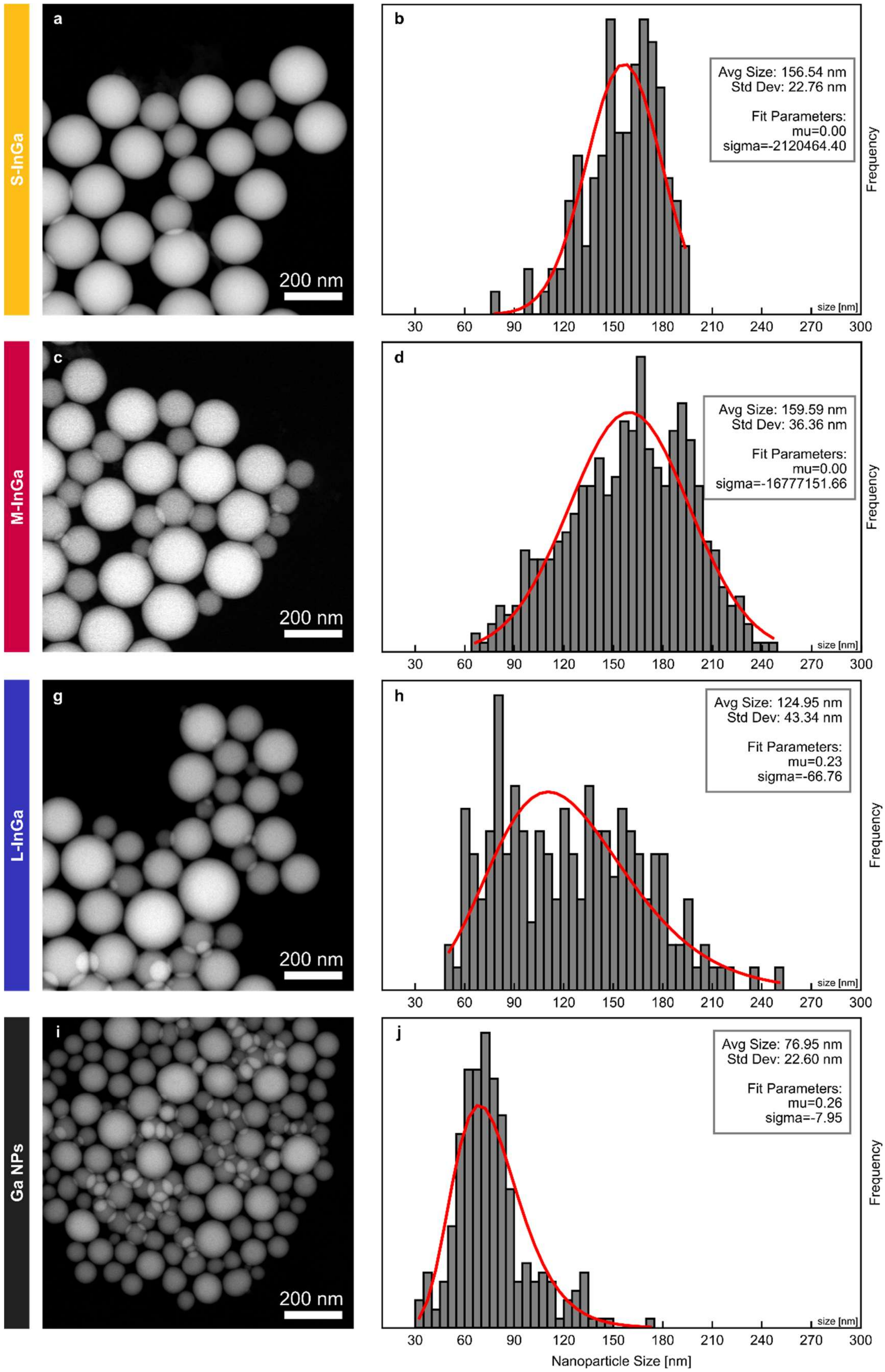

Figure S2 – Size distribution of the as-synthesized NPs. HAADF-STEM image and size-distribution of **(a,b)** S-InGa, **(c,d)** M-InGa, **(e,f)** L-InGa and **(g,h)** Ga NPs. The size distribution analysis is based on at least 50 NPs.

When both gallium and indium precursors are co-injected, the reaction medium turns dark grey within a few seconds (**Figure S3a,b**) and quenching the reaction at that moment leads to solid indium-rich NPs ($In_{98}Ga_2$ or **S-InGa**, **Figure S2a**), indicating that the color change corresponds to the nucleation of indium. When the gallium precursor is injected alone, a color change indicating gallium nucleation is observed but more gradually, and the color change is complete after *ca.* 30 seconds (**Figure S3c**) indicating that gallium precursor alone can undergo thermal decomposition and produce enough Ga^0 monomer to trigger the nucleation of gallium. But while indium nucleates within a few seconds in the presence of gallium precursor, the injection of indium precursor alone does not trigger any color change (**Figure S3d**) nor generate any indium or indium oxide NPs under the same reaction conditions, even after one hour of reaction (**Figure S3e**). Altogether, these experiments point to the need of co-injecting the two precursors for the formation of the obtained NPs. Some chemical reaction among them might be occurring.

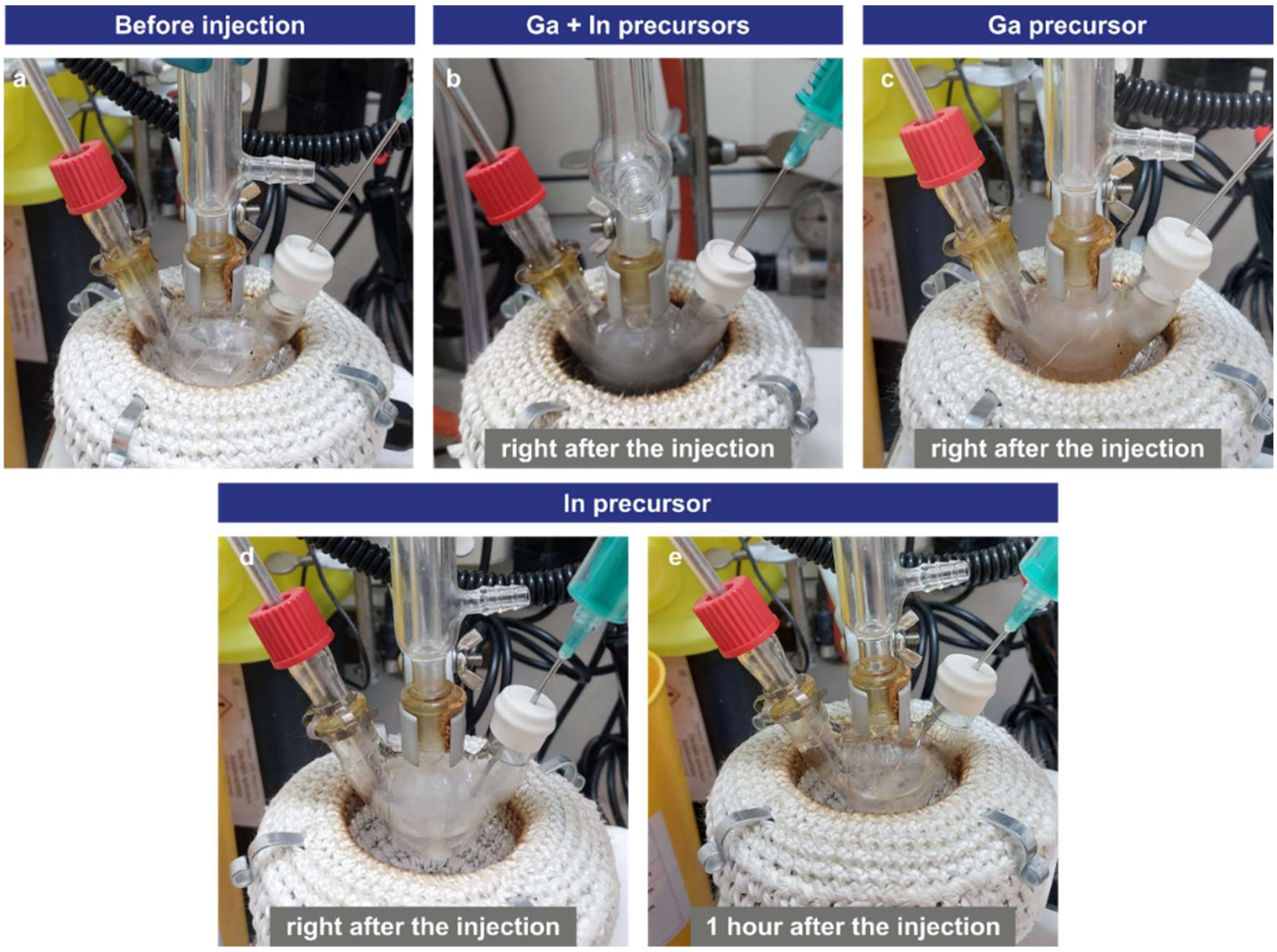

Figure S3 – Comparison of the reaction medium color evolution with and without the gallium precursor. **(a)** Photograph of the reaction flask before precursor injection. **(b,c)** Reaction flask after the precursor injection: **(b)** with gallium and indium precursors **(c)** with only gallium precursor and **(d)** with only indium precursor. **(e)** Reaction flask after 1 hour with only indium precursor, in the absence of gallium precursor. The co-injection of gallium and indium precursors caused an immediate color change to dark gray. In contrast, injecting the gallium precursor alone led to a gradual darkening over about one minute, while the solution remained transparent even after one hour when only the indium precursor was injected.

Sequential injection (within *ca.* 30 seconds) of the indium precursor followed by the gallium precursor let to NPs of similar composition and size than the co-injection (**Figure S4a,b**). On the other hand, the sequential injection of the gallium precursor followed by the indium precursor upon color change led to hollowed Ga NPs containing a crystalline indium core (**Figure S4c,d**). The standard reduction potentials of gallium and indium ($E^0_{Ga^{3+}/Ga} = -0.53$ V *vs*. SHE and $E^0_{In^{3+}/In} = -0.34$ V *vs* SHE) could suggest that a galvanic replacement reaction coupled with the Kirkendall effect might be occurring in this case.[9,10]

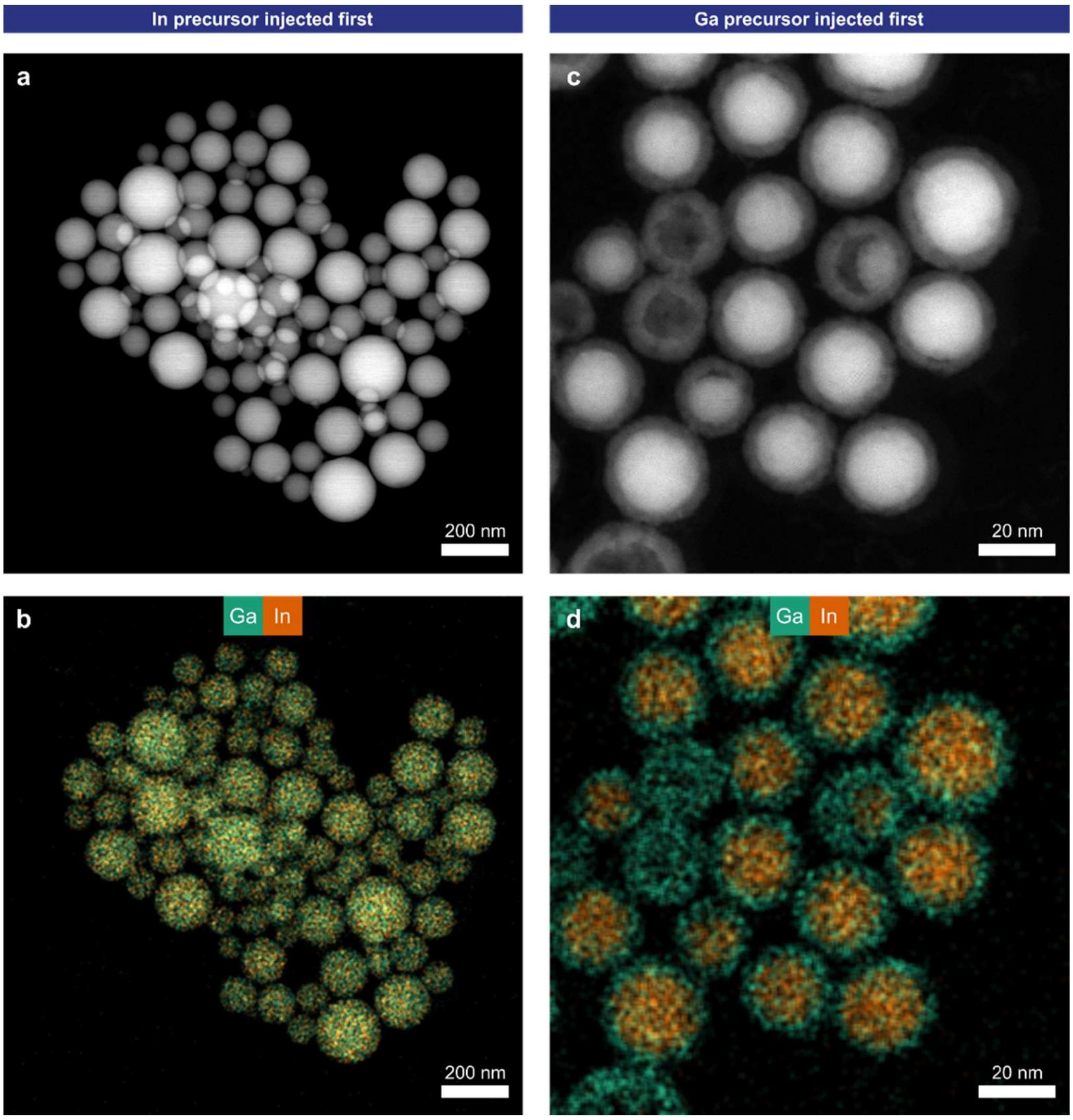

Figure S4 – In-Ga NPs obtained by sequential injection of indium and gallium precursors. (a,b) HAADF-STEM and corresponding elemental maps of the NPs obtained by injection of the indium precursor followed by the injection of gallium precursor. **(c,d)** HAADF-STEM and corresponding elemental maps of the NPs obtained by injection of the gallium precursor followed by the injection of indium precursor upon color change.

Another possible reaction between the precursors could be a ligand exchange between TDMAG and $InBr_3$, leading to the *in situ* formation of a long-chain indium amide that subsequently undergoes thermal decomposition to generate In^0 monomers.

In the literature, the formation of long-chain indium amides from $InBr_3$ under reaction conditions similar to ours has been reported when oleylamine is first deprotonated with *n*-BuLi prior to reaction with $InBr_3$; however, in that case, the resulting indium amide was too stable to decompose in the absence of a strong reducing agent.[11]

In the present system, invoking such a pathway would therefore require (i) efficient ligand exchange between the gallium and indium precursors and (ii) thermal instability of the *in situ* formed indium amide under our reaction conditions.

In the hypothesis that the indium amide is effectively formed during the reaction, we tested the thermal stability of the formed indium amide by conducting the following experiment (**Figure S5**): in a reaction flask, ODE and an excess of DOA were dried at 110°C for 1 hour under vacuum. Then the flask was filled with nitrogen and heated to 290°C before injecting 1 mL of *n*-BuLi in 2.7M heptane. This injection aimed at deprotonating the DOA. After waiting 30 seconds to ensure all the *n*-BuLi is consumed, a solution of 28 mg (0.079 mmol) of $InBr_3$ in 2 mL ODE and 2.26 mL DOA was swiftly injected. The reaction medium changed color indicating that some nucleation happened. HAADF-STEM showed the formation of indium particles. This experiment demonstrates that an eventual ligand exchange occurring between TDMAG and $InBr_3$ could effectively be responsible for the indium nucleation.

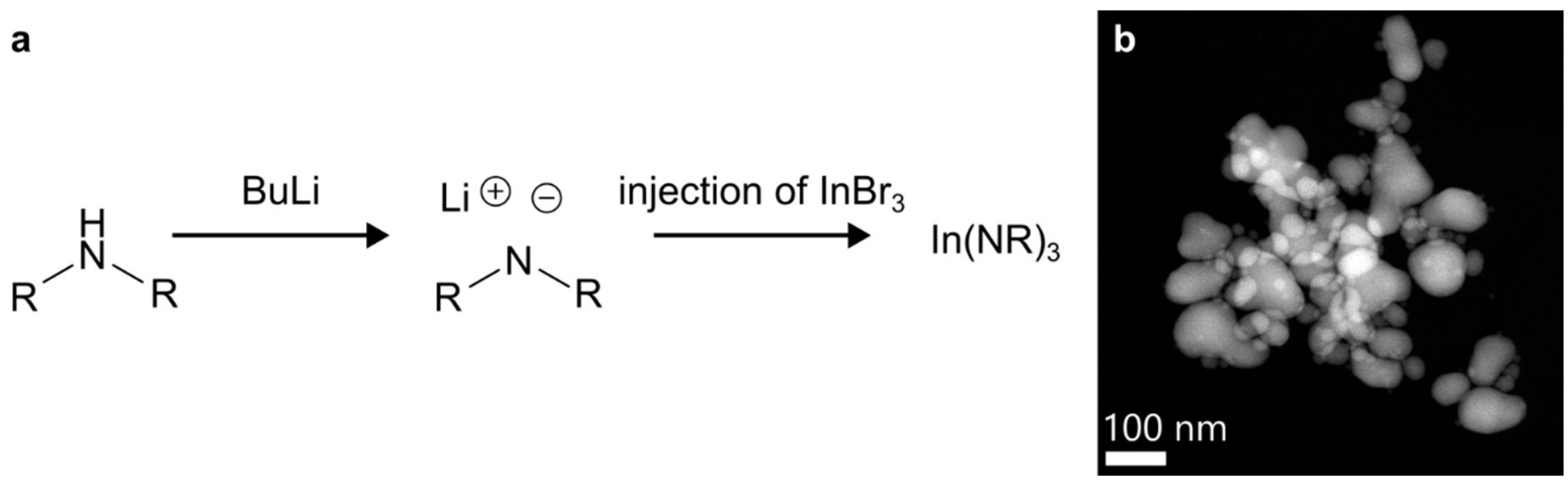

Figure S5 – Indium particles formation through *in situ* generation of indium long-amide species. (a) Synthesis schematics **(b)** HAADF-STEM image of the obtained particles.

Based on all the above observations, we propose two complementary mechanisms (**Figure S6**). The first one is a redox-driven mechanism (**Figure S6a**), where the thermal decomposition of the Ga precursor into Ga^0 monomers occurs first. Then, the oxidation of these Ga species reduces the indium precursor into In^0 which triggers In NP nucleation with following incorporation of Ga as time proceeds. The second one relies on the *in situ* generation of indium amide species able to thermally decompose (**Figure S6b**). Future studies are needed to elaborate about the mechanism in more details.

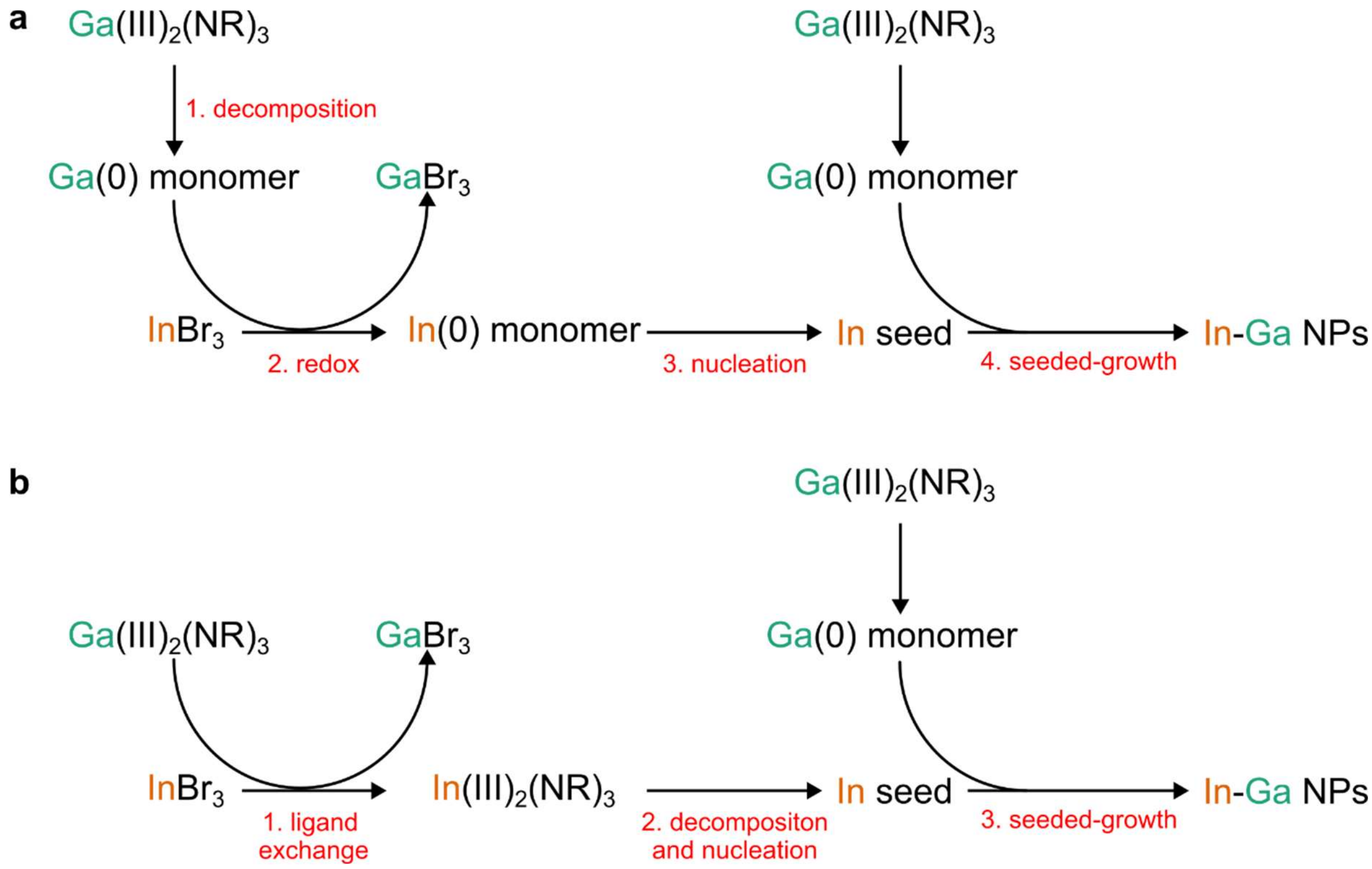

Figure S6 – Proposed reaction mechanisms for the formation of the In-Ga NPs. (a) Redox-driven mechanism. First, the gallium precursor undergoes thermal decomposition to produce Ga^0 monomers. The indium precursor is subsequently reduced, while the gallium monomers are oxidized to $GaBr_3$. **(b)** Decomposition of *in situ* formed indium amide. The gallium and indium precursor swap ligands, leading to the formation of indium amide, able to decompose thermally. In both cases, as the concentration of In^0 monomers increases, indium nucleates, providing seeds for the excess Ga^0 monomers to grow. This process leads to the formation of In–Ga NPs, with the gallium content increasing as the reaction proceeds.

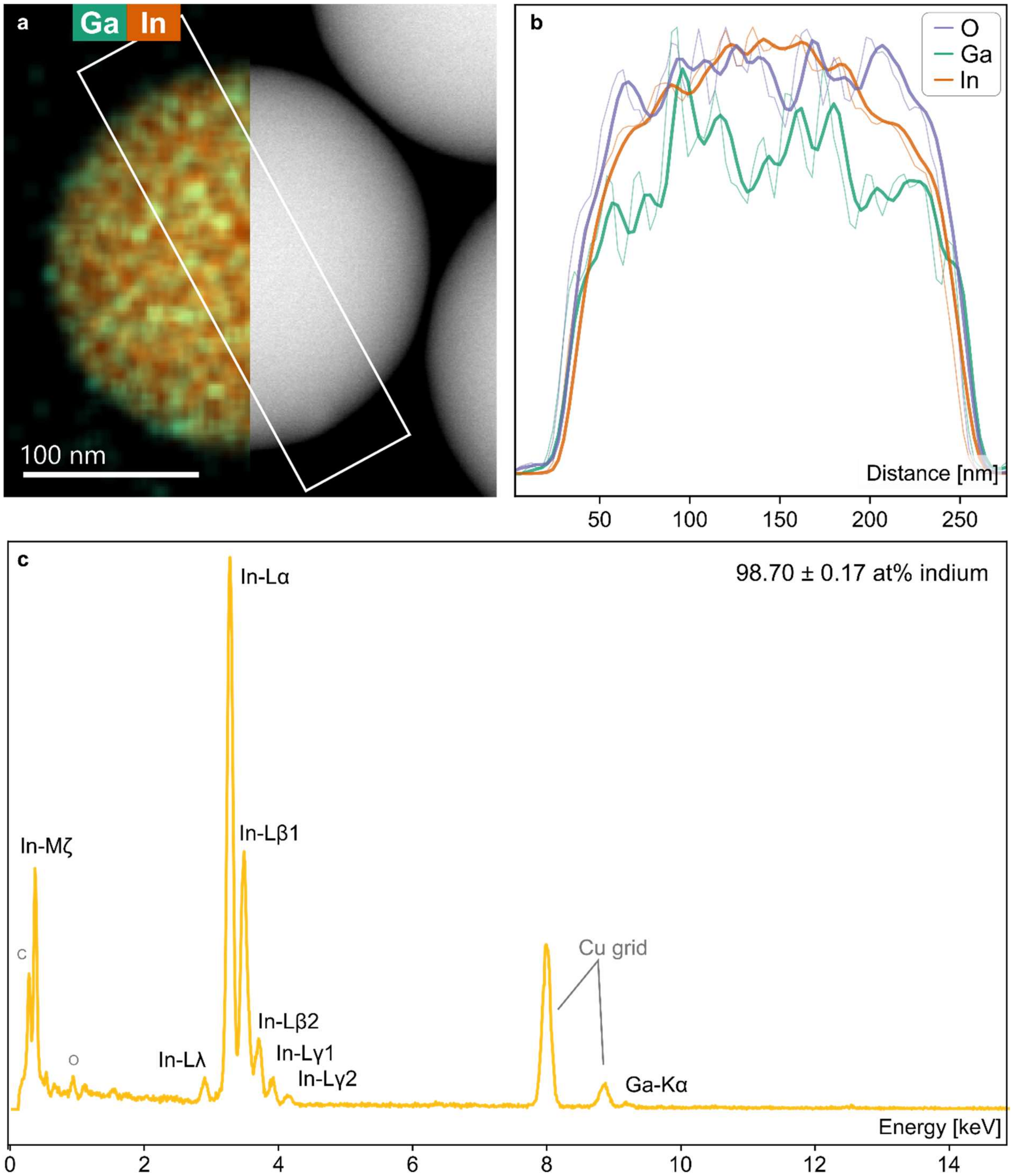

Figure S7 – HAADF STEM and EDXS of as-synthesized S-InGa. (a) EDXS (green: gallium, orange: indium) and HAADF STEM of one NP. **(b)** Normalized line profile corresponding to the white rectangle area in **a**. **(c)** EDXS spectrum of the NP imaged in **a**.

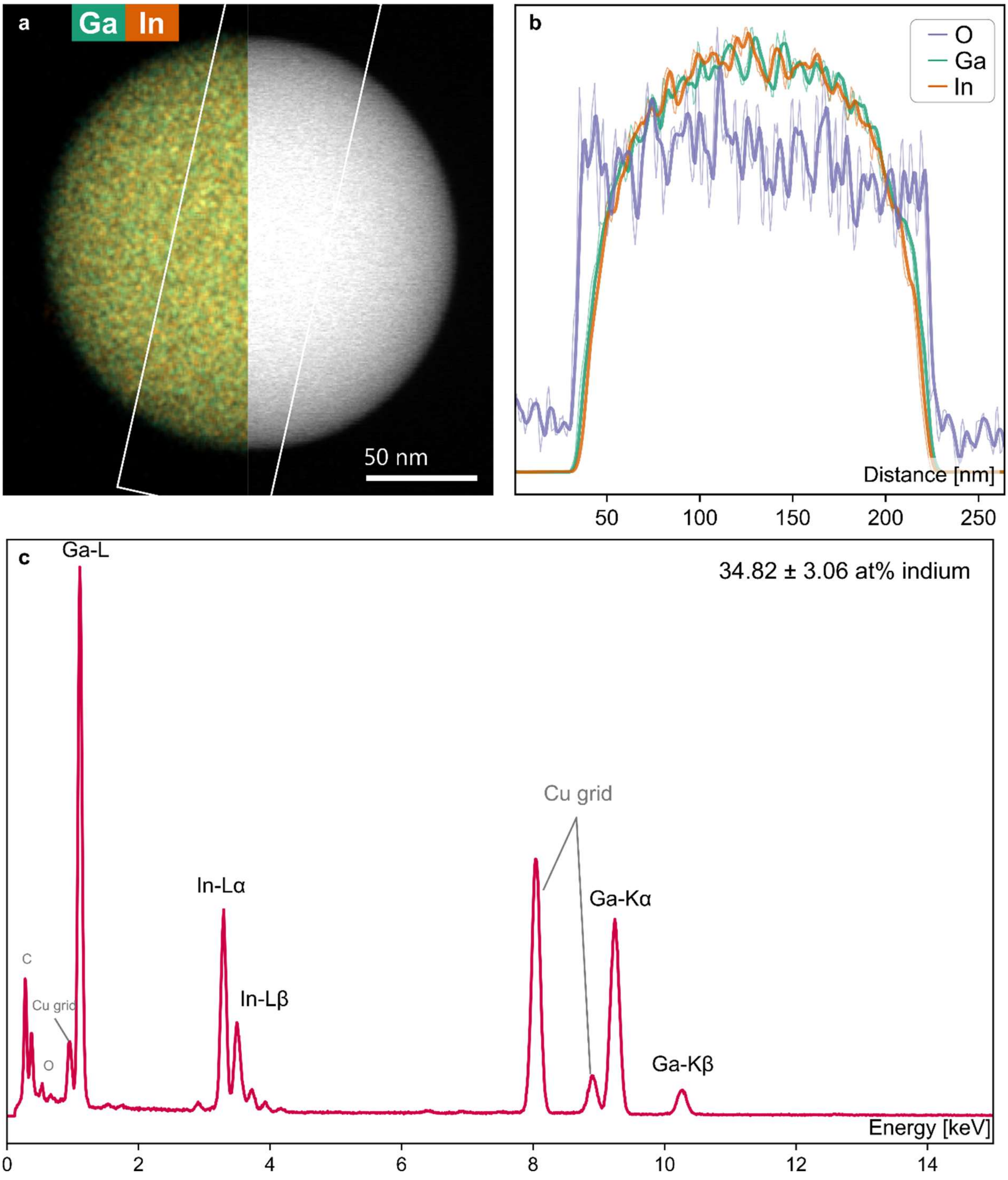

Figure S8 – HAADF STEM and EDXS of as-synthesized M-InGa. (a) EDXS (green: gallium, orange: indium) and HAADF STEM of one NP. **(b)** Normalized line profile corresponding to the white rectangle area in **a**. **(c)** EDXS spectrum of the NP imaged in **a**.

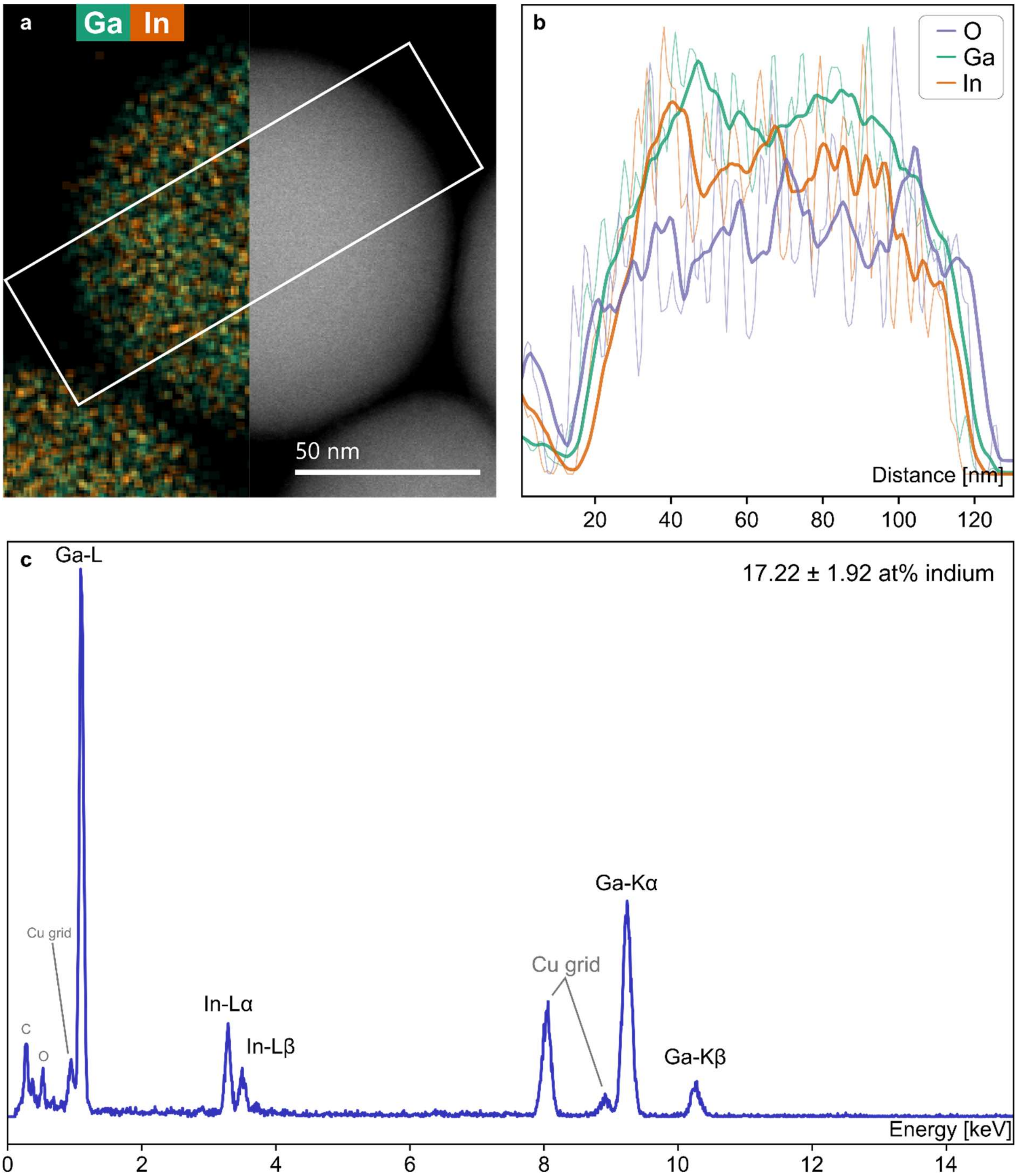

Figure S9 – HAADF STEM and EDXS of as-synthesized L-InGa. (a) EDXS (green: gallium, orange: indium) and HAADF STEM of one NP. **(b)** Normalized line profile corresponding to the white rectangle area in **a**. **(c)** EDXS spectrum of the NP imaged in **a**.

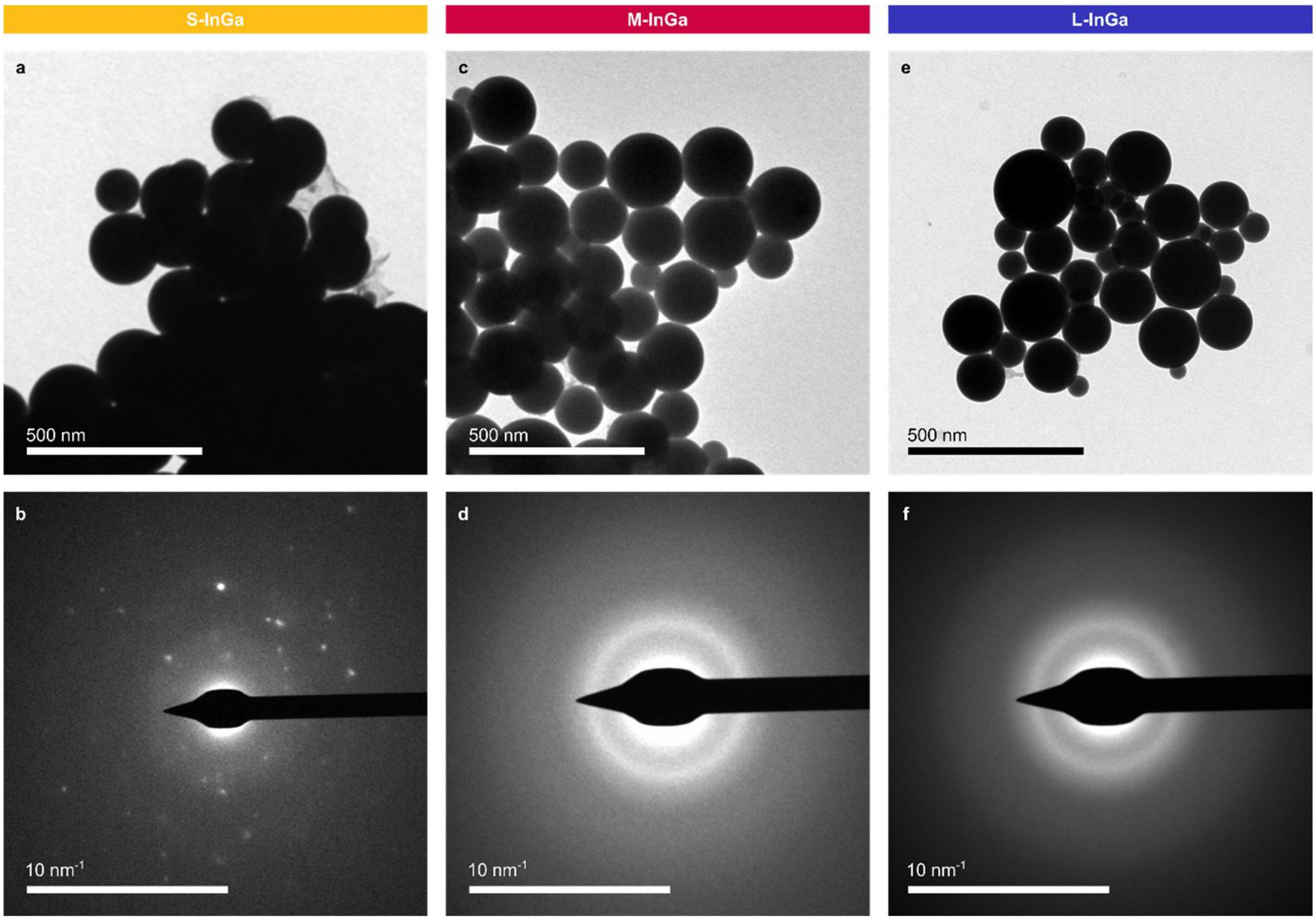

Figure S10 – TEM images and corresponding SAED pattern for **S-InGa (a,b)**, **M-InGa (c,d)** and **L-InGa (e,f),** showing crystalline features only for **S-InGa**.

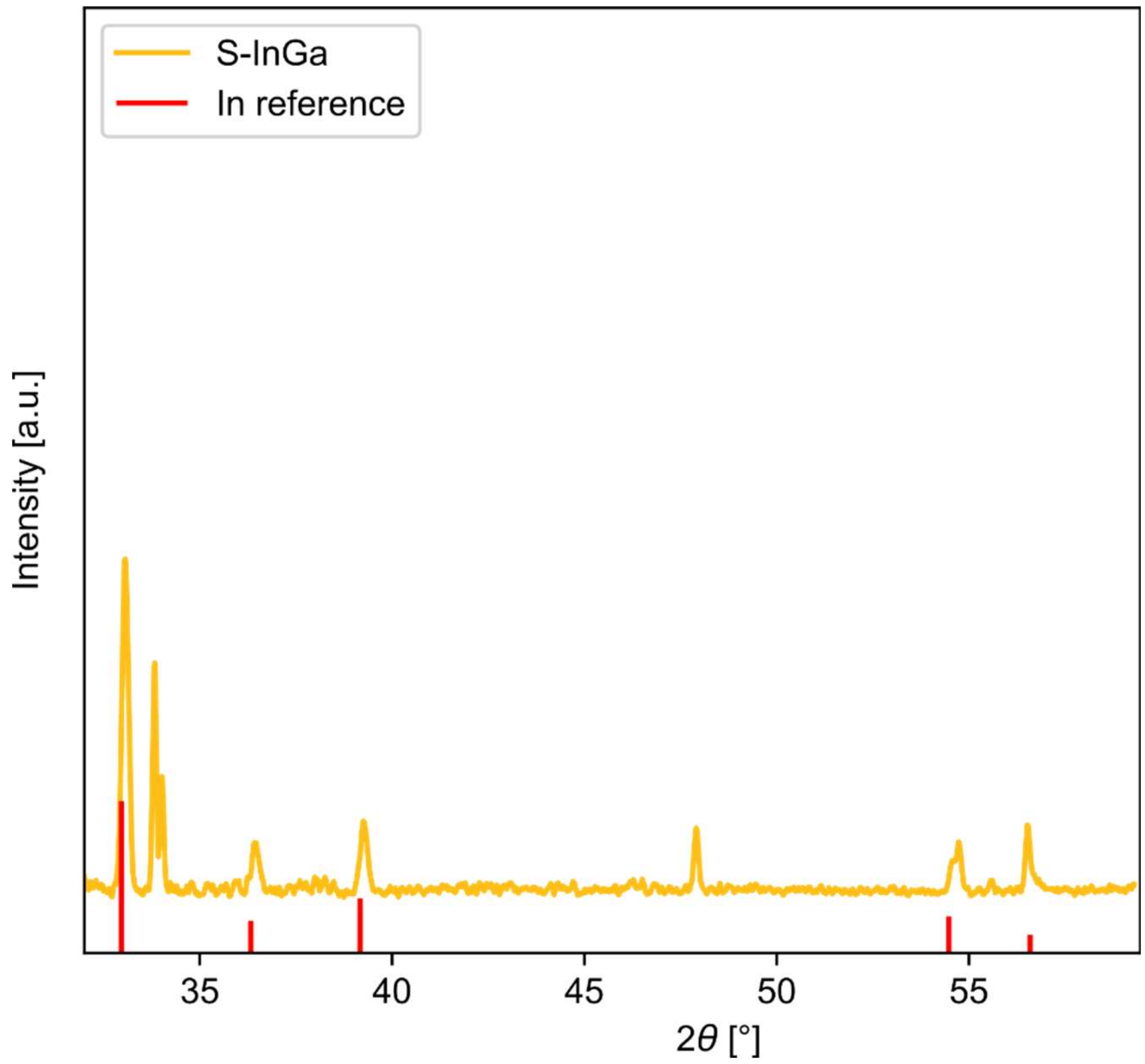

Figure S11 – XRD pattern of as-synthesized S-InGa. The red lines correspond to the indium reference. The peak at 33° is attributed to the silicon wafer.

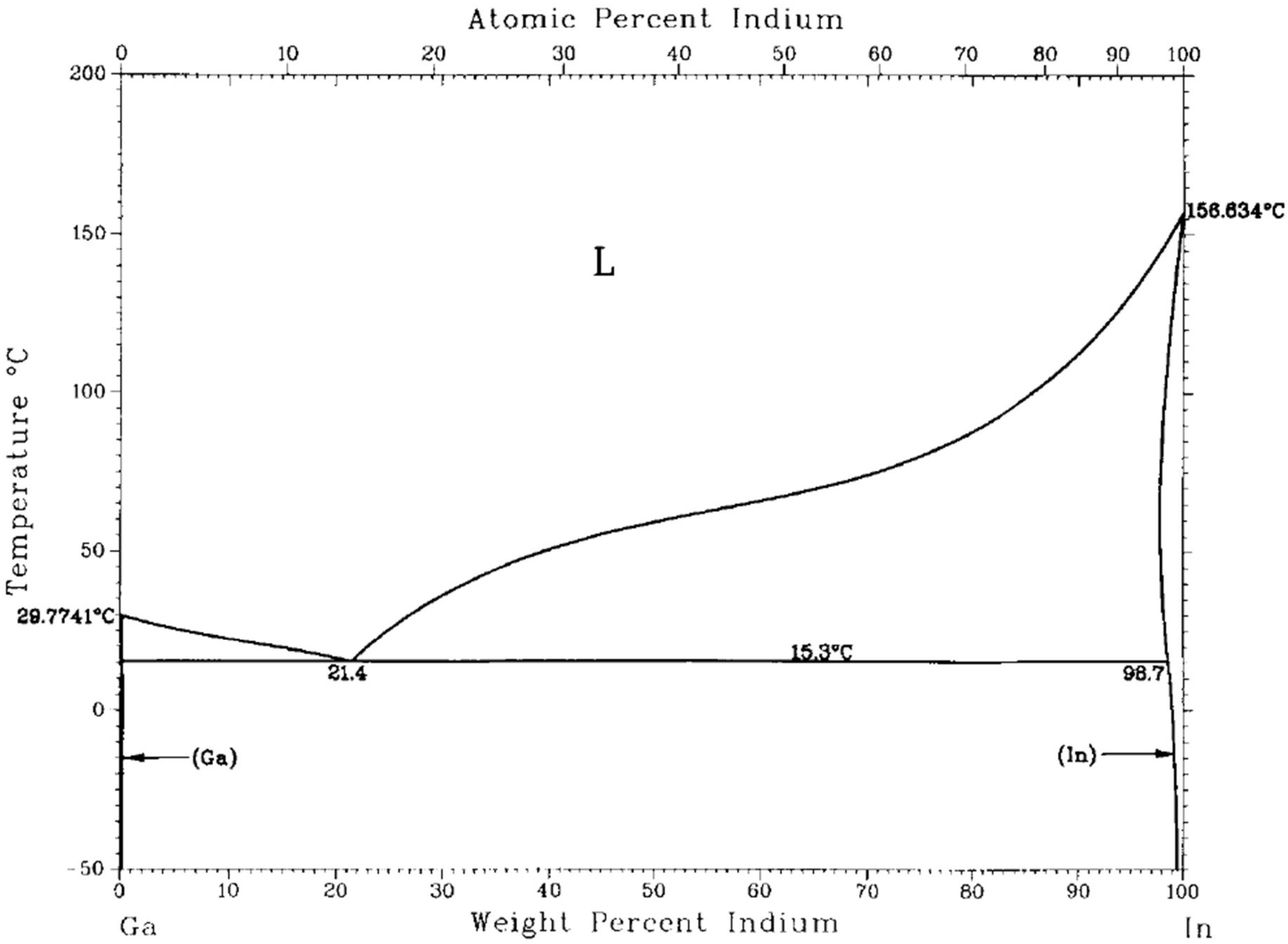

Figure S12 – In-Ga binary phase diagram. Taken from ref.[7]

Note S2 – Supercooling and size effect

The phase behavior of the synthesized In-Ga NPs deviates significantly from that of their bulk counterparts (**Figure S12**). Two main effects could explain this observation: supercooling, a kinetic phenomenon, and size effects, a thermodynamic phenomenon. These two factors jointly determine whether a given LM alloy remains liquid or solid under specific conditions.

In addition to the cryo-STEM EDXS experiment presented in the main manuscript **Figure. 2**, we performed a series of experiments to determine whether the intermediate compositions $In_{0.40}Ga_{0.60}$ (**M-InGa**) were in the liquid state due to supercooling or to nanoscale size effects. To test the supercooling hypothesis, mechanical stress was induced by immersing the NPs in liquid nitrogen, before leaving them to return to room temperature. The NPs were then imaged by HAADF-STEM, and the corresponding images are presented in **Figure S13**. After immersion in liquid nitrogen, the NPs are in a phase segregated state droplet (**Figure S13e-h, Figure S14**), with a solid indium-rich core occasionally observed to move within the surrounding Ga-rich liquid (**Figure S15** and **Figure S16**). This implies that the particles went from a metastable liquid state to a stable solid-liquid biphasic structure. Therefore, the homogeneous structure observed in the as-synthesized NPs does not arise from size effects, but rather from the synthesis process itself, where the NPs form at high temperature before the reaction medium is cooled to room temperature.

However, size effects can still exist and generate a reduction of the temperature of the liquidus and solidus lines of the phase diagram. For example, XRD spectra recorded on the $In_{98}Ga_{2}$ composition (**S-InGa**) while heating the sample (**Figure S17**) evidenced a melting point between 130°C and 140°C, at a lower temperature than the bulk melting temperature of pure indium (156.6°C).[7] Besides, HAADF-STEM coupled with EDXS mapping at different temperature (**Figure S18** and **Figure S19**) also showed transitions shifted to lower temperatures. Therefore, the XRD and electron microscopy experiments both suggest that a size effect modifies the Ga-In phase diagram at nanoscale.

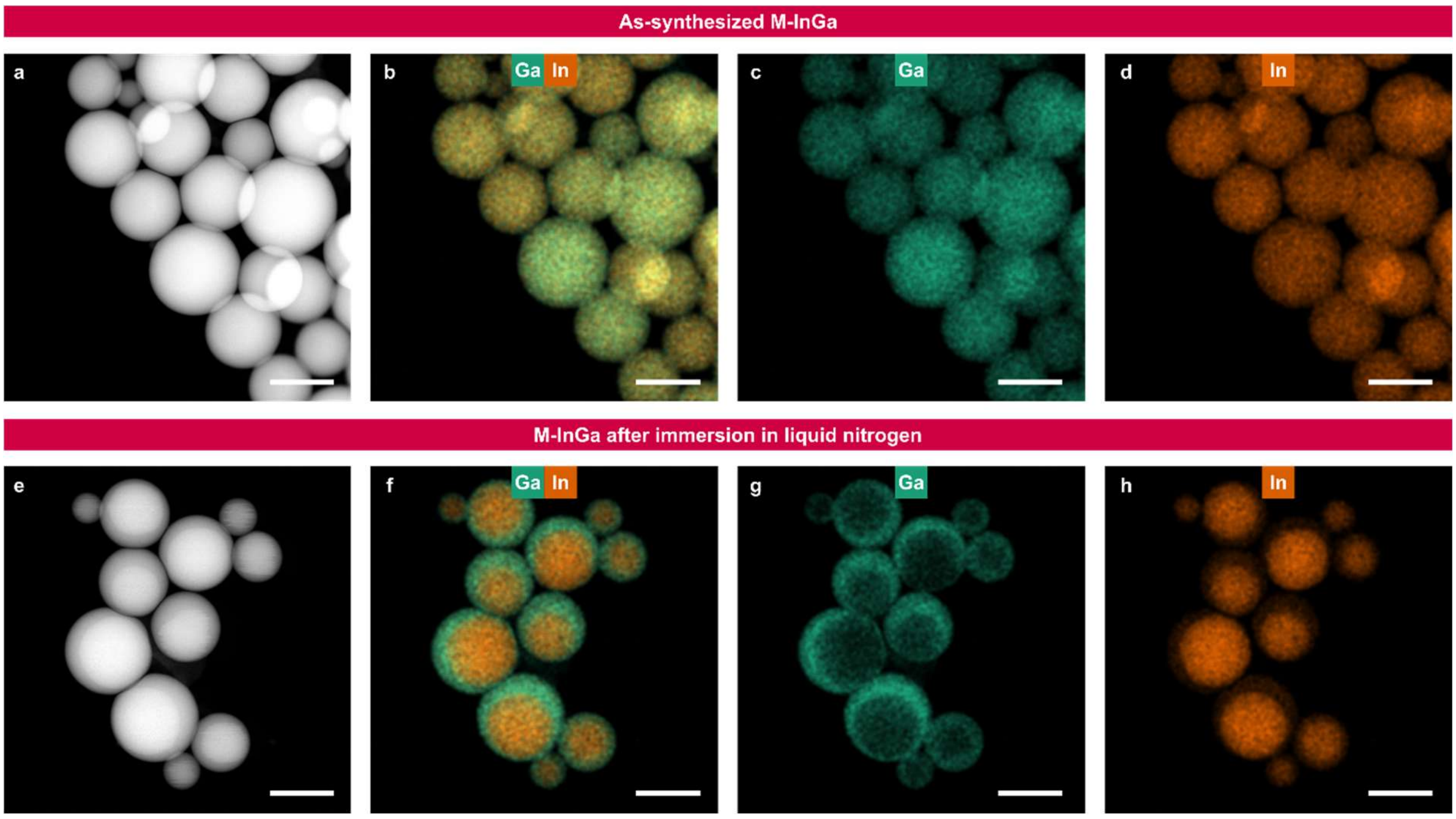

Figure S13 – Phase segregation of the metastable In-Ga NPs when immersed in liquid N_2. HAADF-STEM images **(a)** and corresponding elemental mapping (**(b)** In and Ga, **(c)** Ga and **(d)** In) of the as-synthesized **M-InGa**. HAADF-STEM images **(e)** and corresponding elemental mapping (**(f)** In and Ga, **(g)** Ga and **(h)** In) of **M-InGa** after immersion in liquid nitrogen. The scale bar is 200 nm.

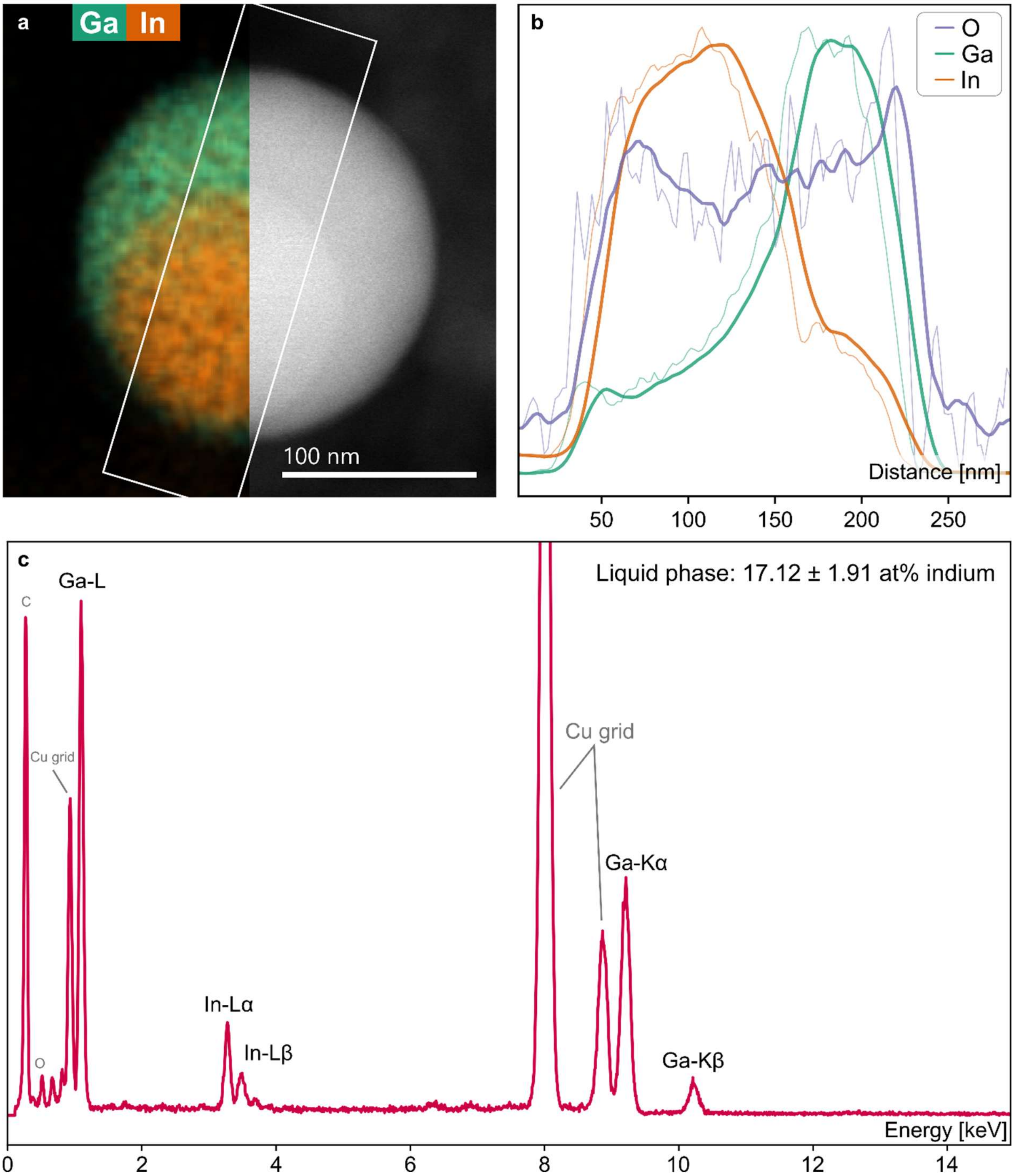

Figure S14 – HAADF STEM and EDXS of M-InGa after triggering its phase segregation by immersion in liquid nitrogen. (a) EDXS (green: gallium, orange: indium) and HAADF STEM of one NP. **(b) Normalized** line profile corresponding to the white rectangle area in **a**. **(c)** EDXS spectrum of the NP imaged in **a**.

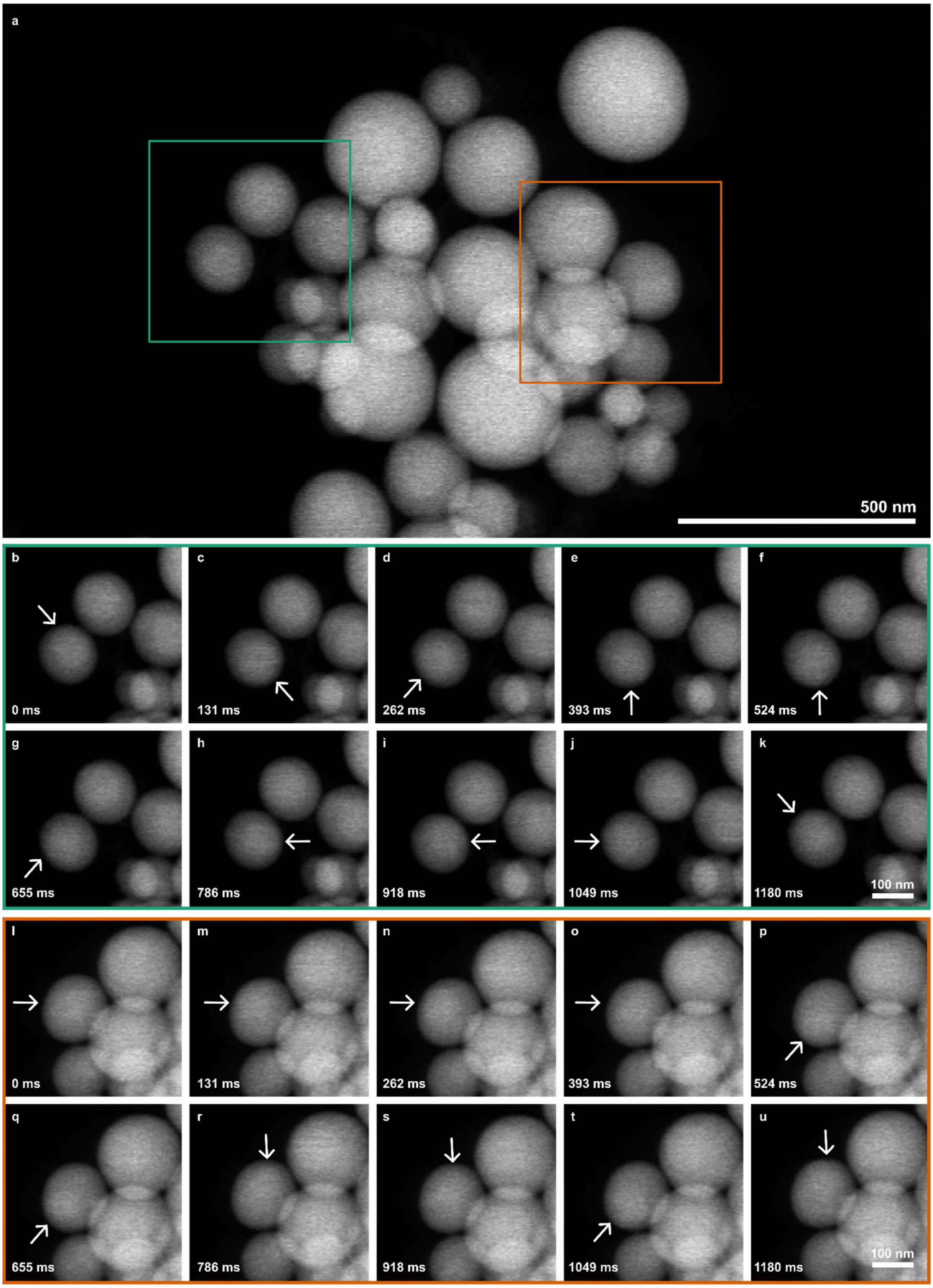

Figure S15 – Moving cores within M-InGa NPs after immersion in liquid nitrogen. (a) HAADF-STEM image **M-InGa** at room temperature after immersion in liquid nitrogen and **(b-u)** snapshots of two different regions over time, showing the bright core moving within the gallium NPs.

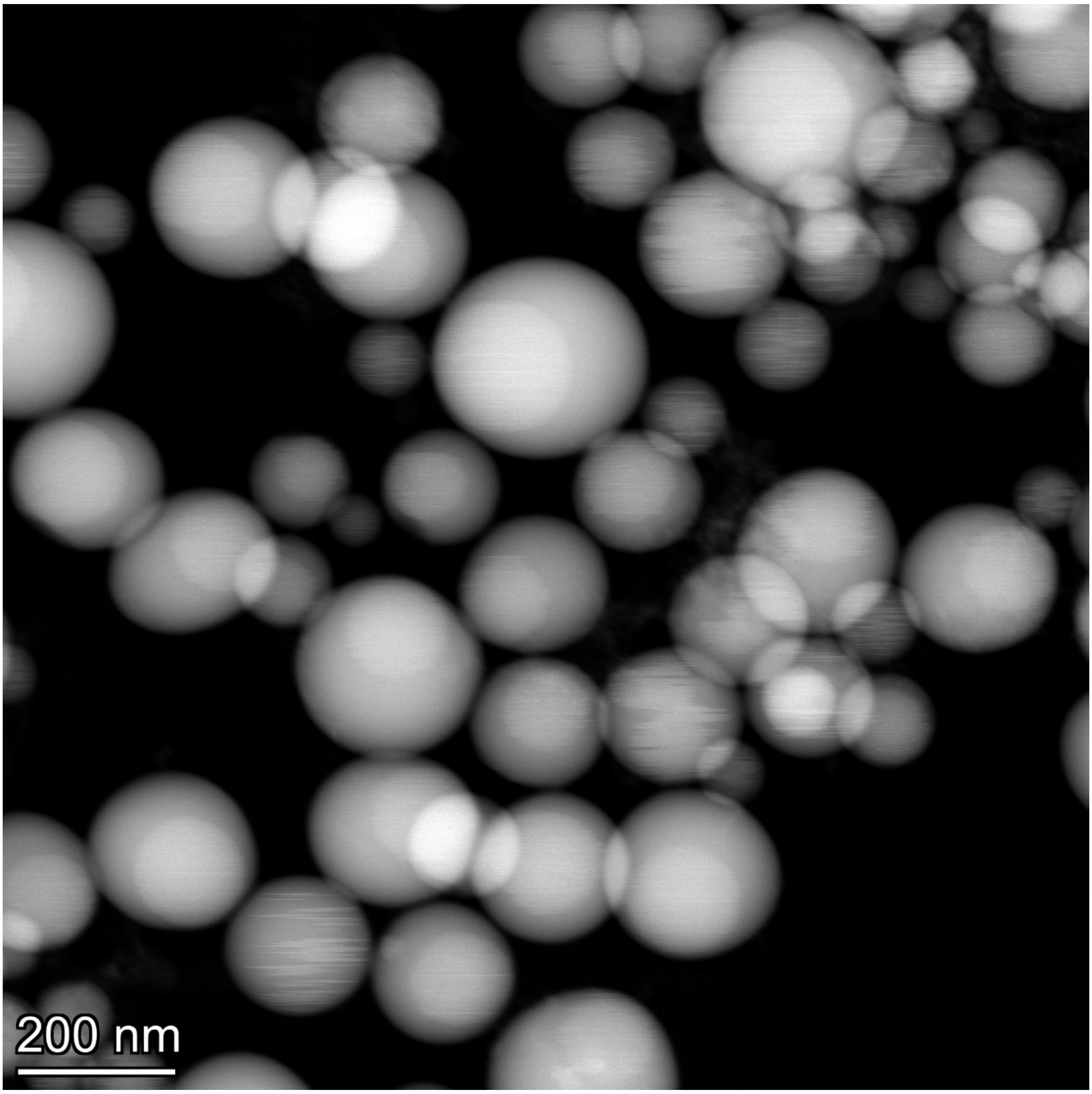

Figure S16 – HAADF-STEM images **M-InGa** at room temperature after immersion in liquid nitrogen. The moving cores appear as blurry with horizontal lines due to their movements during the scanning of the image.

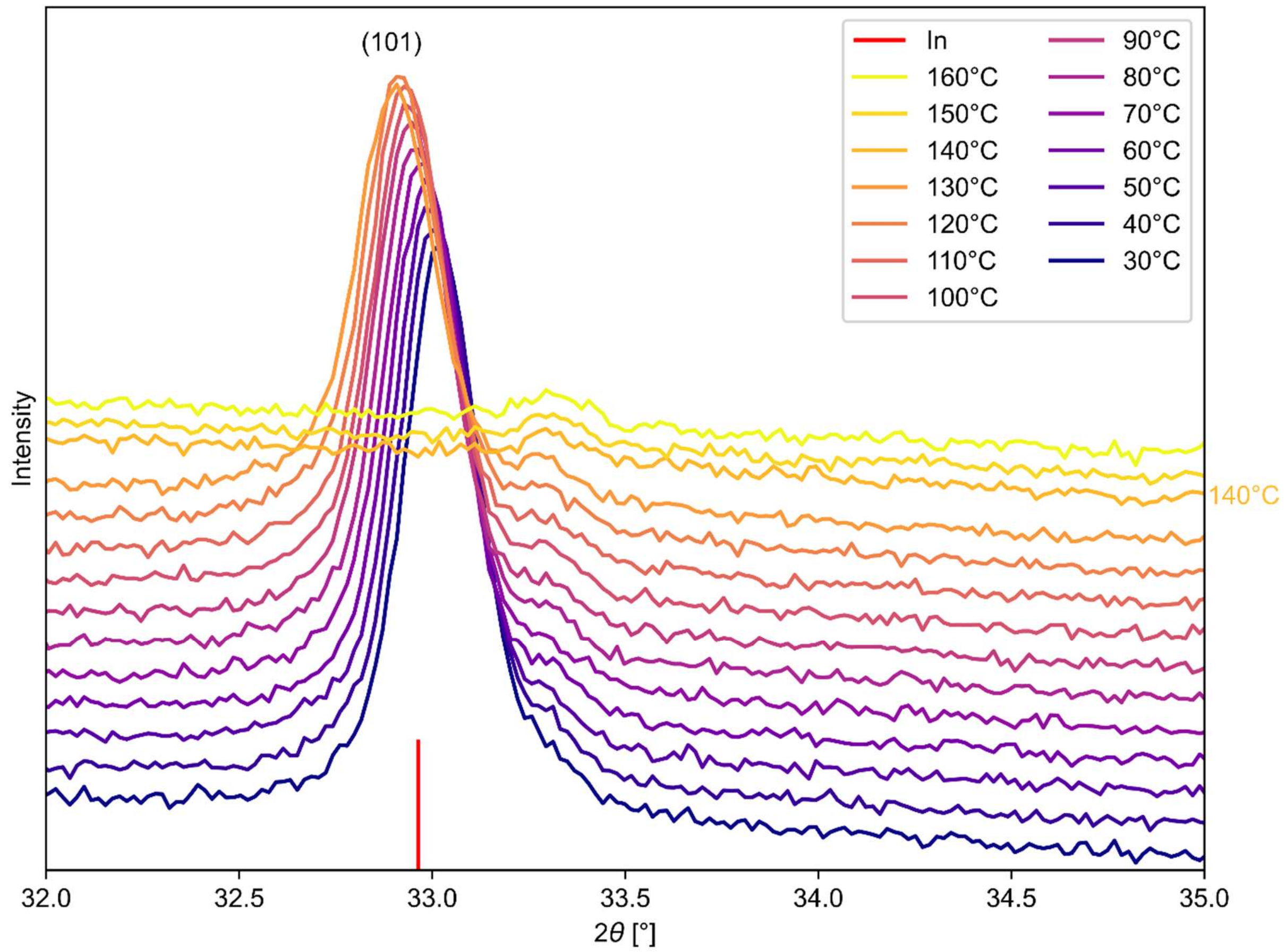

Figure S17– XRD patterns collected while heating S-InGa from room temperature to 160°C. The (101) diffraction peak disappears between 130°C and 140°C, at a lower temperature than the bulk melting temperature of pure indium (156.6°C). The slight shift of the peak maximum originates from the thermal expansion of the film.

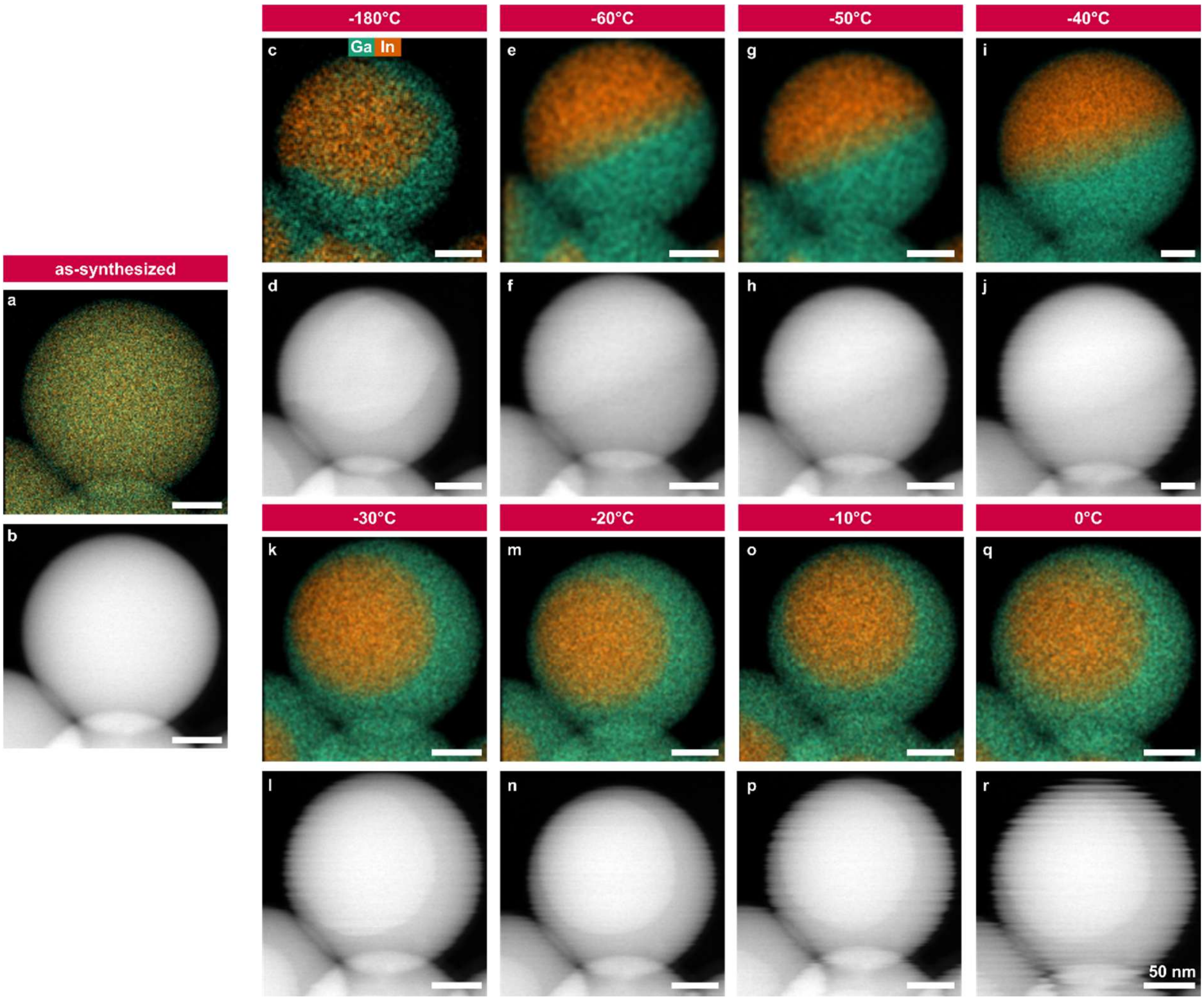

Figure S18 – Additional cryo-STEM imaging on a M-InGa NP. HAADF-STEM images and corresponding elemental maps (orange: indium, green: gallium) of one NP **(a,b)** as-synthesized, **(c,d)** at -180°C, **(e,f)** at -60°C, **(g,h)** at -50°C, **(i,j)** at -40°C, **(k,l)** at -30°C, **(m,n)** at -20°C, **(o,p)** at -10°C and **(q,r)** at 0°C.

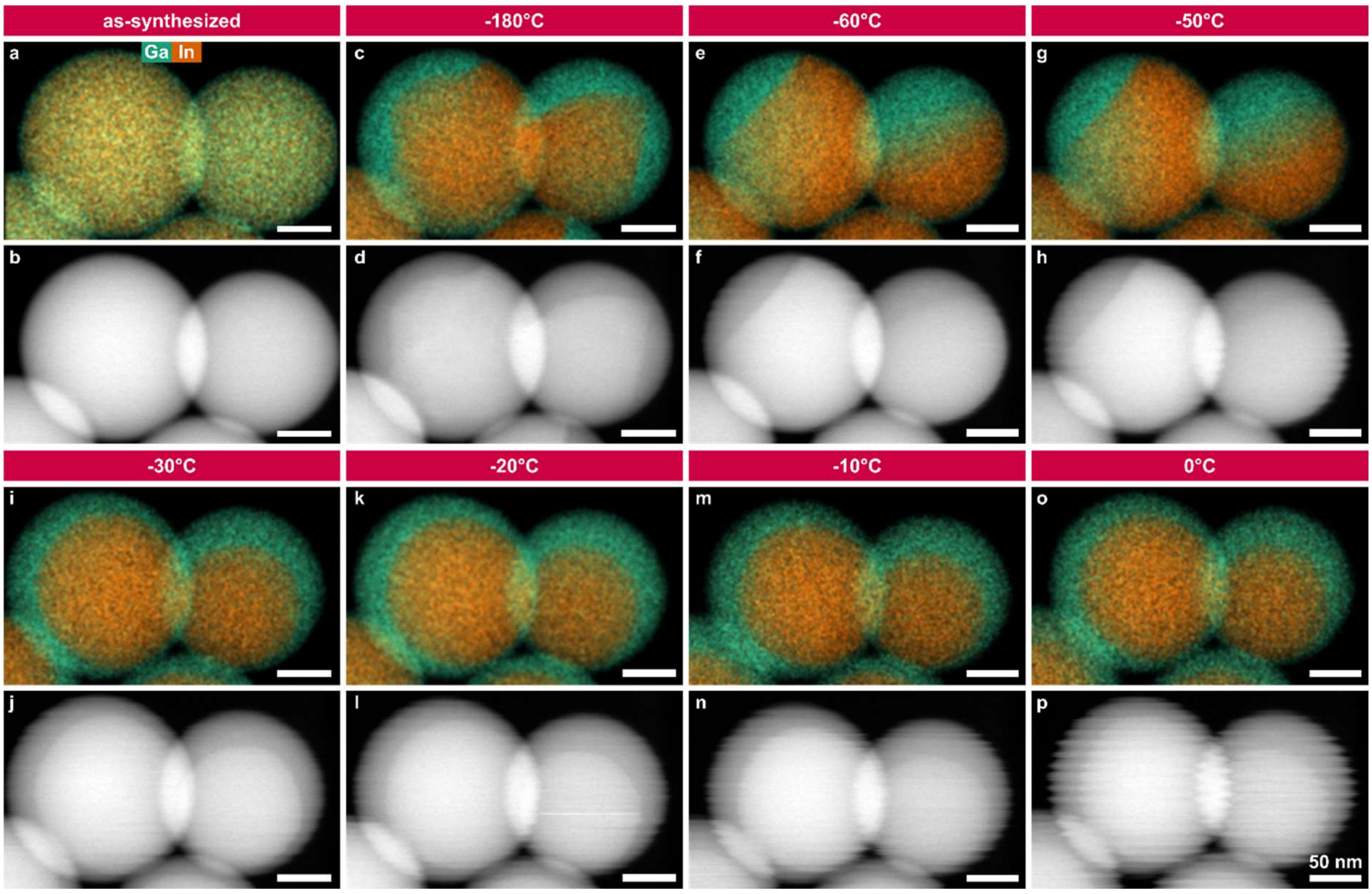

Figure S19 – Additional cryo-STEM imaging on two M-InGa NPs. HAADF-STEM images and corresponding elemental maps (orange: indium, green: gallium) of the two NPs **(a,b)** as-synthesized, **(c,d)** at -180°C, **(e,f)** at -60°C, **(g,h)** at -50°C, **(i,j)** at -30°C, **(k,l)** at -20°C, **(m,n)** at -10°C and **(o,p)** at -0°C.

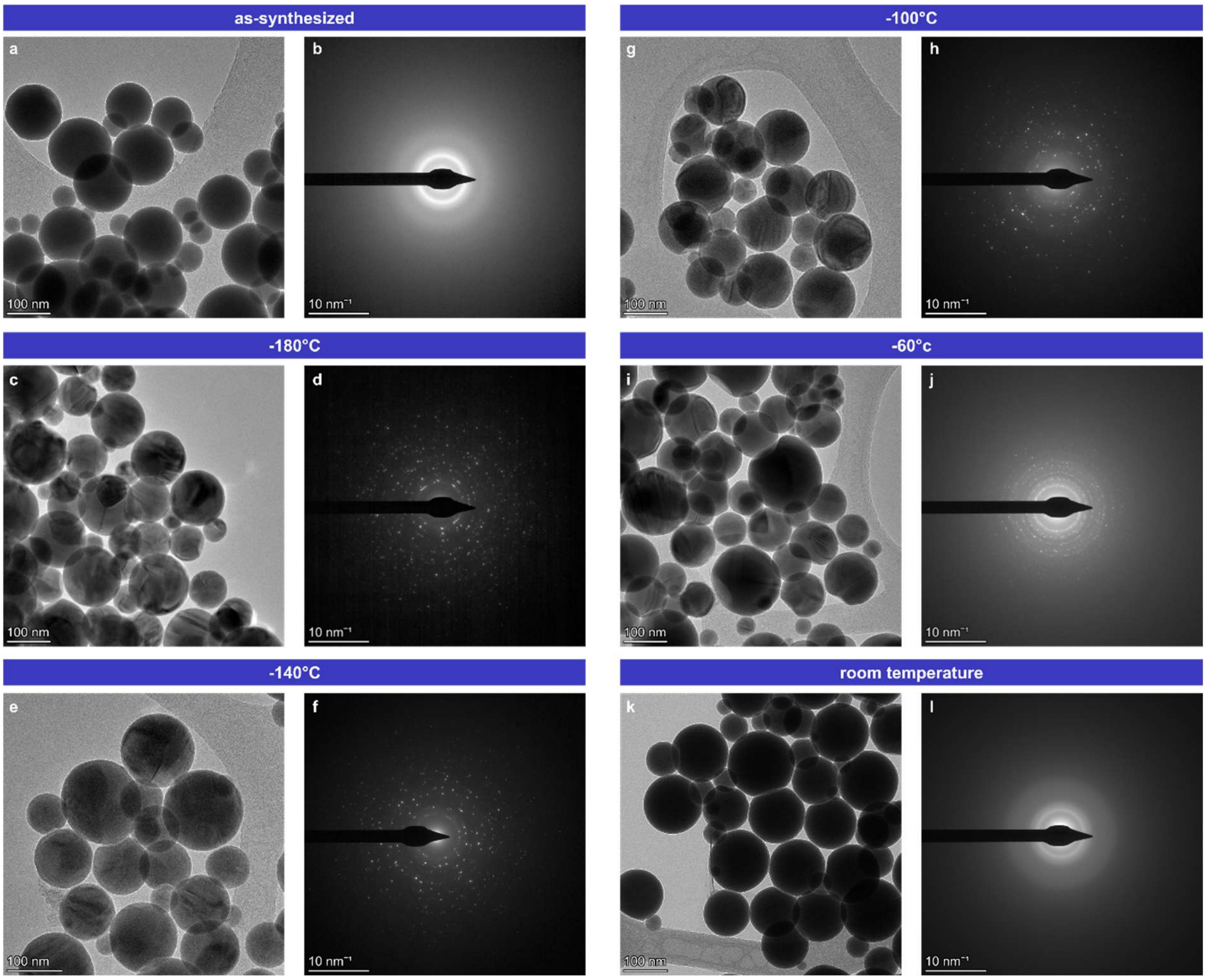

Figure S20 – Cryo-TEM imaging and diffraction patterns of L-InGa. (a,b) TEM image and corresponding diffraction pattern for the as-synthesized **L-InGa** NPs TEM image and corresponding diffraction pattern **(a,b)** as synthesized, **(c,d)** at -180°C, **(e,f)** -140°C, **(g,h)** -100°C, **(i,j)** -60°C and **(k,l)** back to room temperature. This experiment shows that the as-synthesized fully liquid **L-InGa** NPs are stable at room temperature and not in a supercooled state.

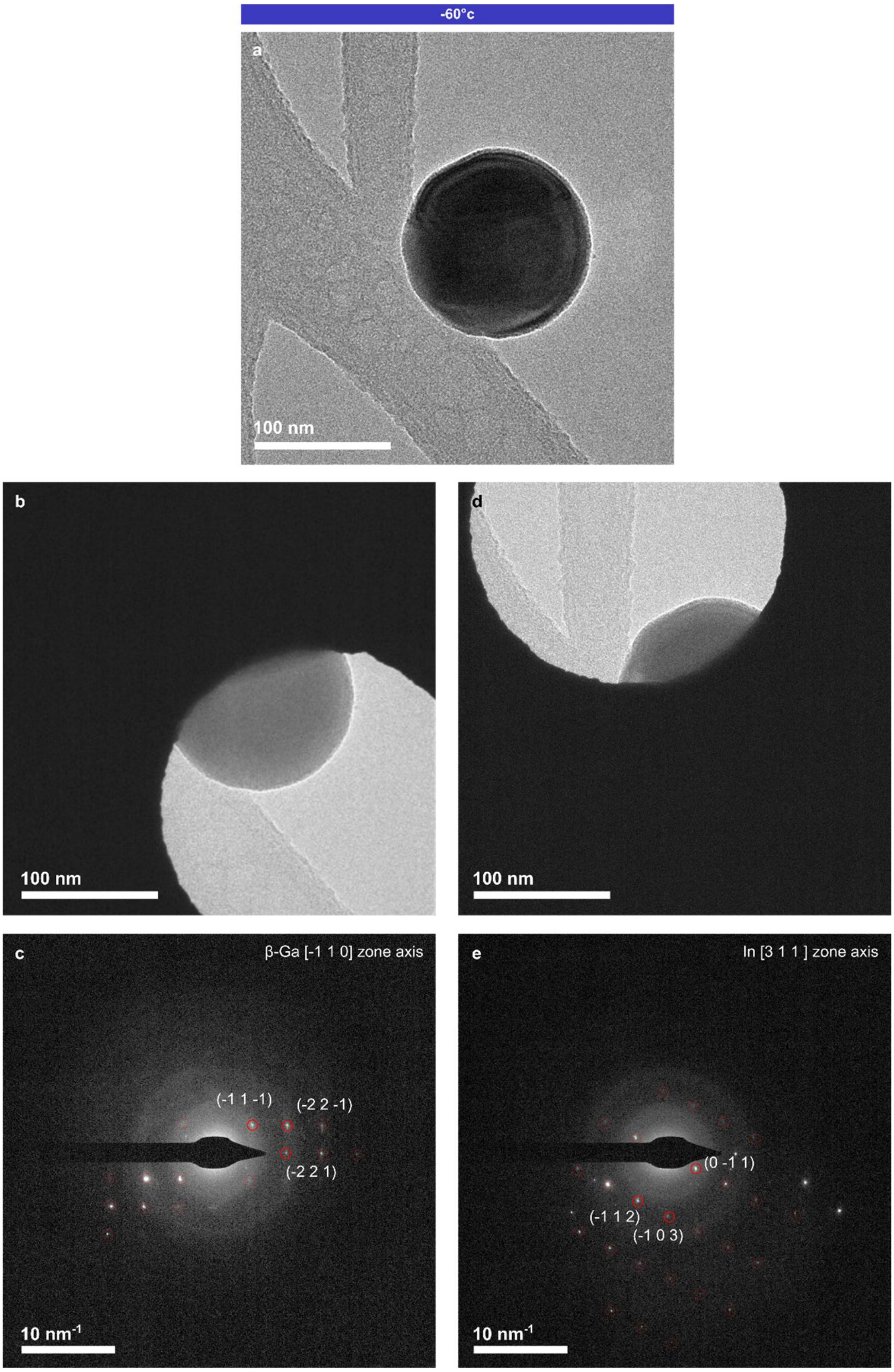

Figure S21 – Phase identification in L-InGa at -60°C. (a) TEM image of one NP. **(b)** TEM image corresponding to the less electron dense domain in the particle showing the position of the selected area aperture used to obtain the diffraction pattern in **(c). (c)** Diffraction pattern allowing to identify the β-Ga phase **(d)** TEM image corresponding to the more electron dense part of the particle showing the position of the selected area aperture used to obtain the diffraction pattern in **(e)** and **(e)** diffraction pattern allowing to identify the indium phase.

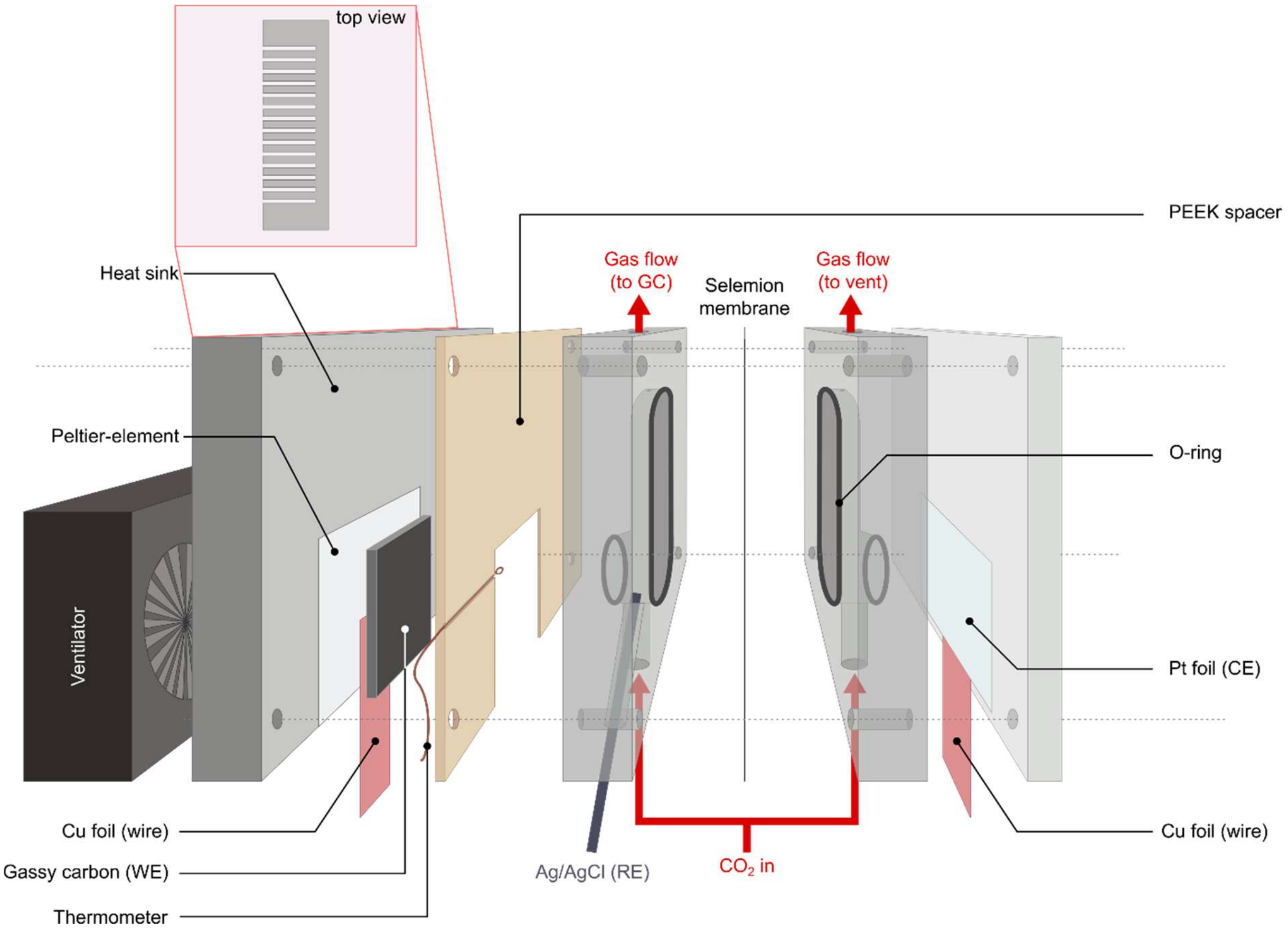

Figure S22 – Electrochemical cell with integrated Peltier-element heating the catalyst-coated working electrode.

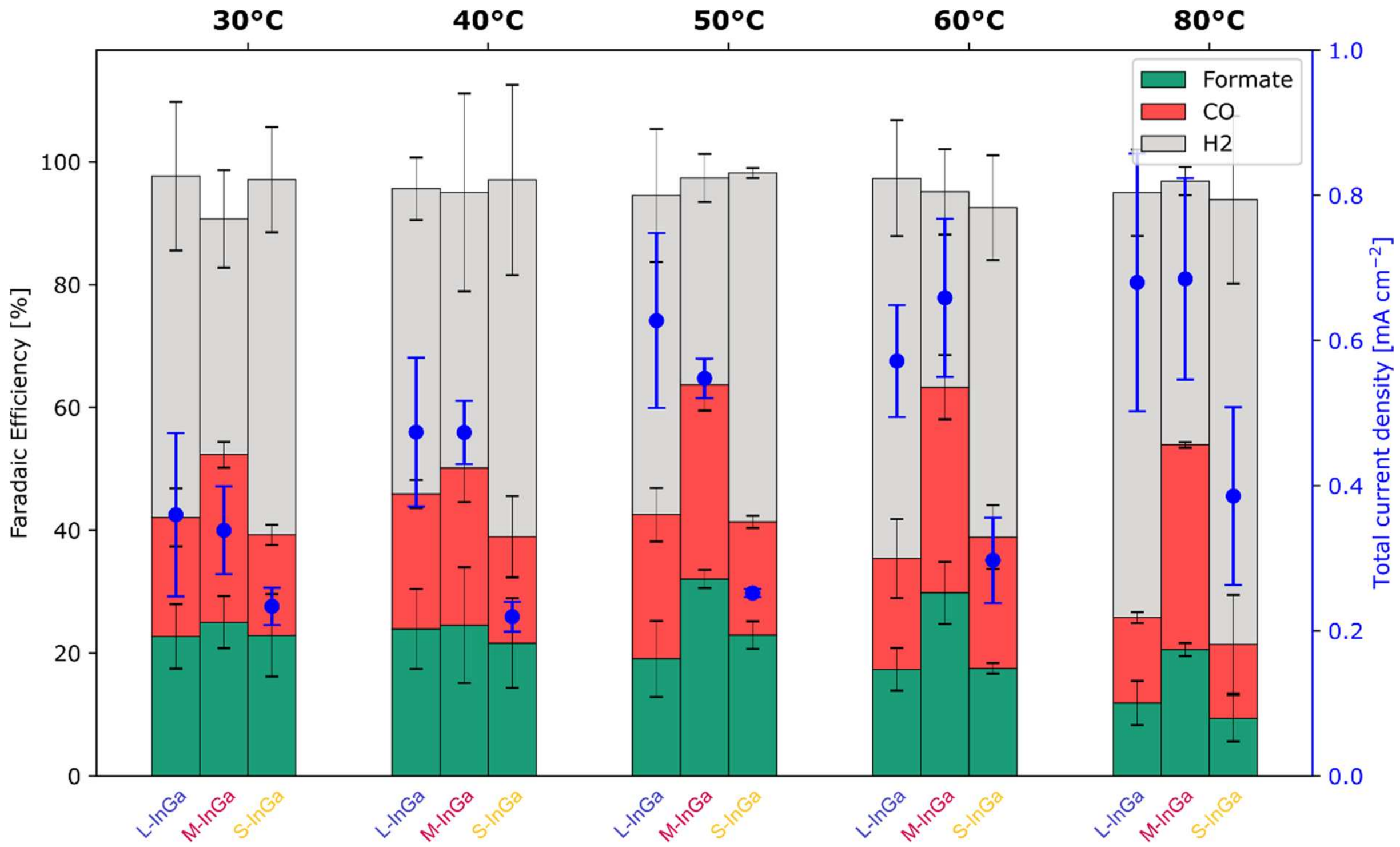

Figure S23 – Effect on temperature on CO_2RR Faradaic efficiency and total current density at -0.7 V_{RHE}. The experiments were performed in a H-cell with a $KHCO_3$ electrolyte at -0.7 V_{RHE}. The data are the average of three independent experiments and the error bars are the calculated standard deviations.

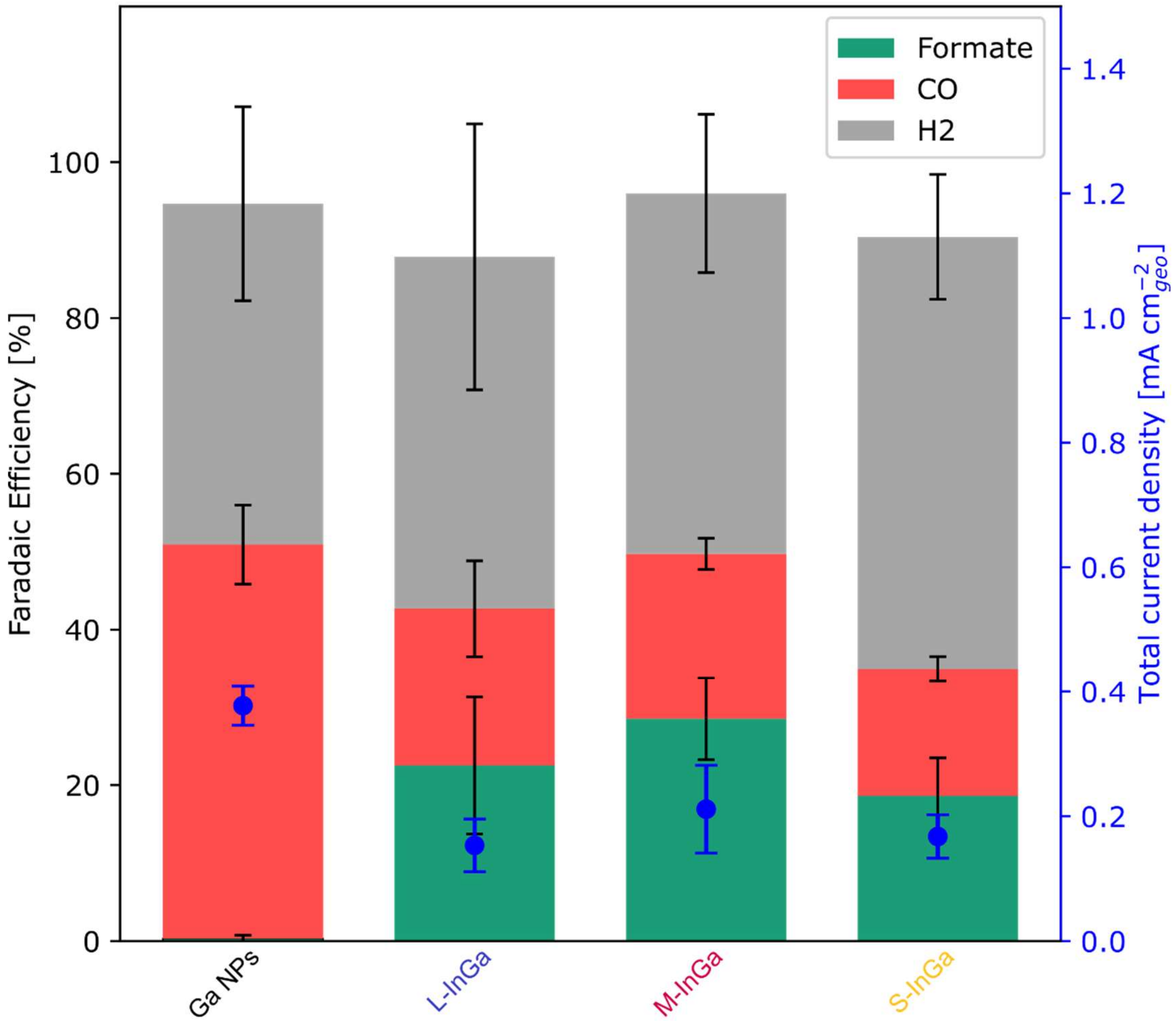

Figure S24 – CO_2RR Faradaic efficiency and total current density at -0.7 V_{RHE}. The experiments were performed in a H-cell with a $KHCO_3$ electrolyte at -0.7 V_{RHE}. The data are the average of three independent experiments and the error bars are the calculated standard deviations.

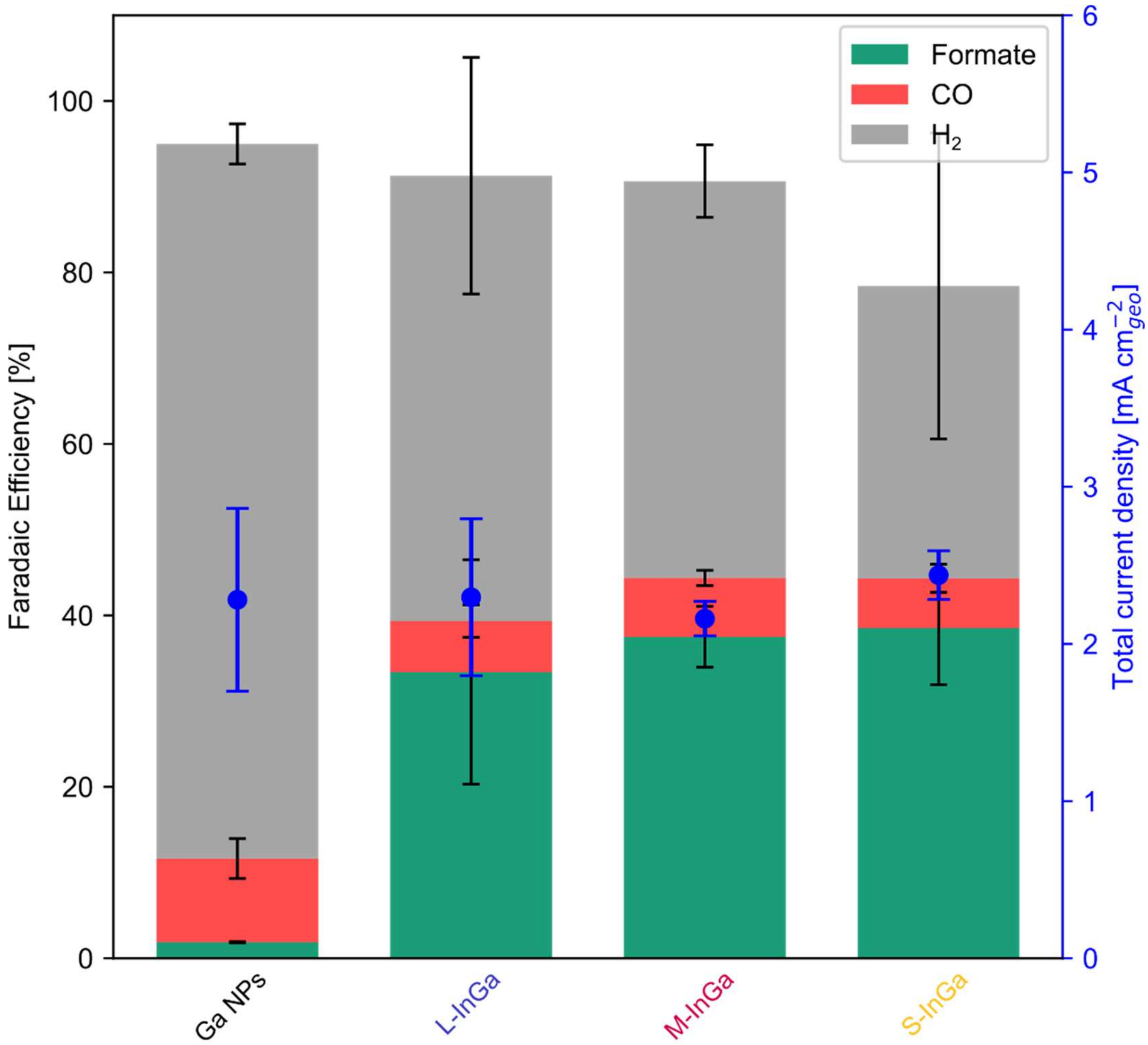

Figure S25 – CO_2RR Faradaic efficiency and total current density at -1.1 V_{RHE}. The experiments were performed in a H-cell with a $KHCO_3$ electrolyte at -1.1 V_{RHE}. The data are the average of three independent experiments and the error bars are the calculated standard deviations.

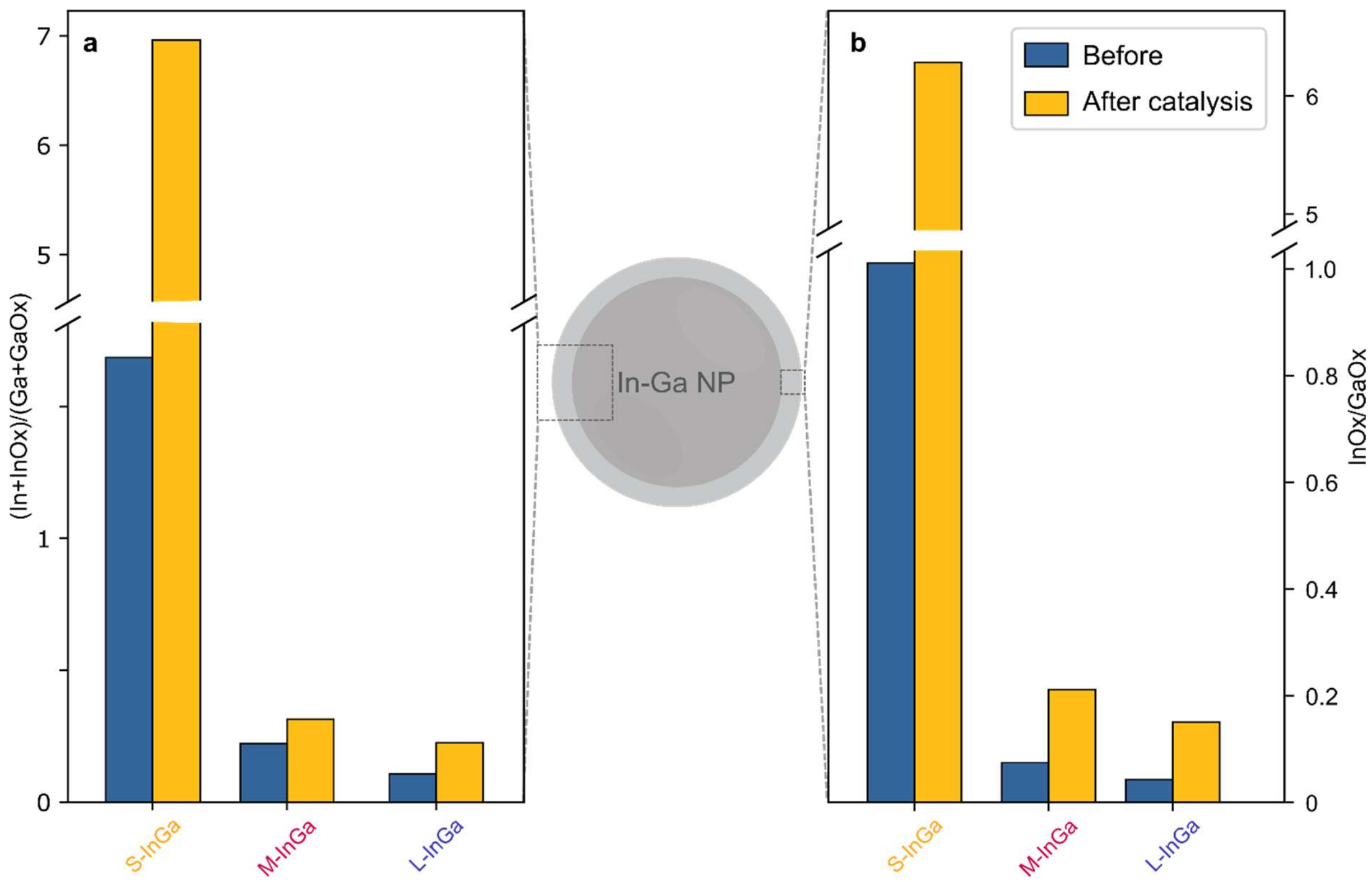

Figure S26 – Surface indium enrichment evidenced by XPS. (a) XPS ratio of the total In 3d signal (In + InOx) to the total Ga 2p signal (Ga + GaOx) before and after catalysis, indicating the relative content of In and Ga within the XPS probing depth. **(b)** XPS ratio of the InOx (In 3d) to GaOx (Ga 2p) signals before and after catalysis, reflecting changes in the oxide shell composition.

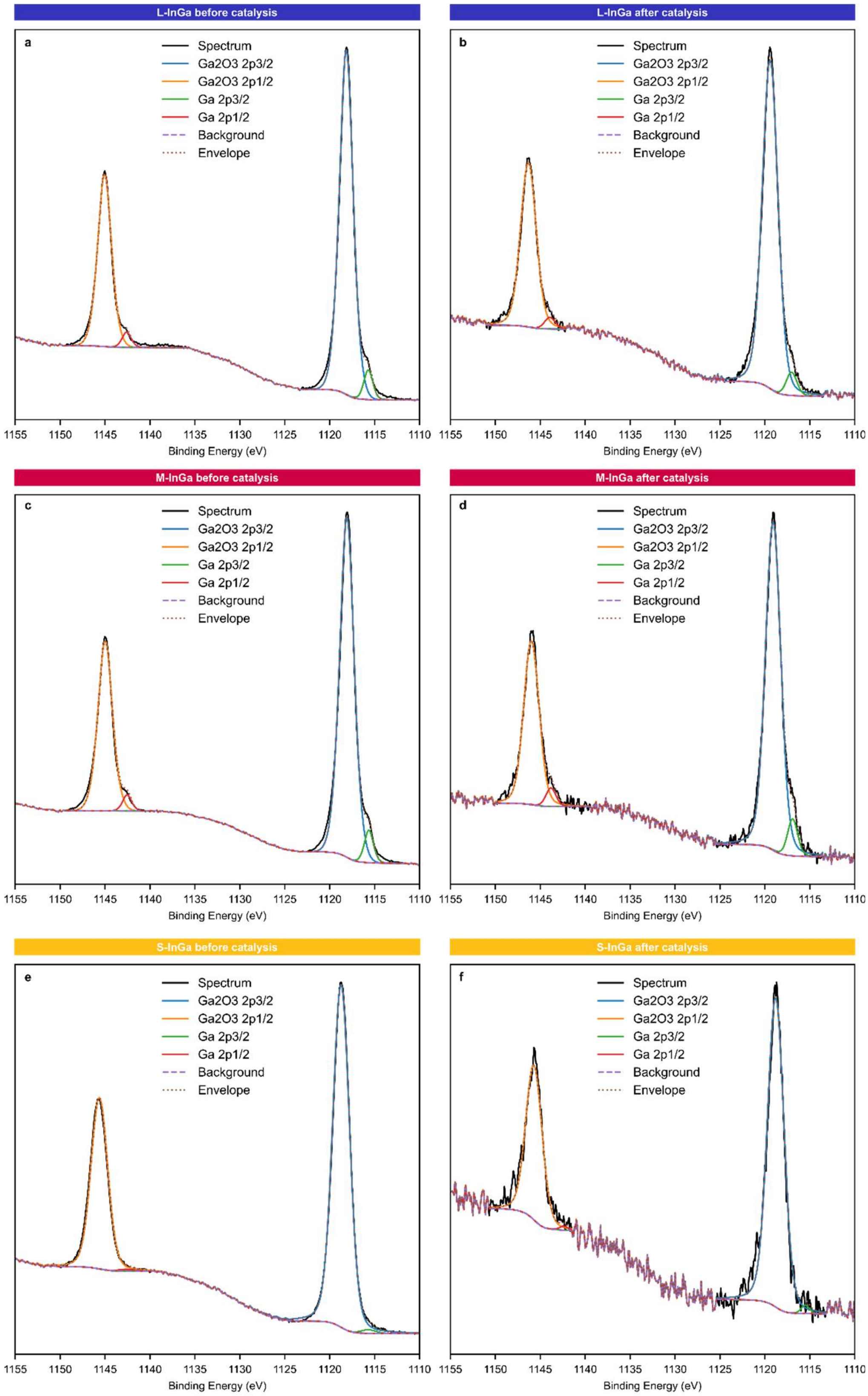

Figure S27 – XPS spectra of the Ga2p region of In-Ga NPs before and after CO2RR at room-temperature. (a,b) XPS spectra together with the fit for **L-InGa** before and after catalysis respectively. **(c,d)** XPS spectra together with the fit for **M-InGa** before and after catalysis respectively. **(e,f)** XPS spectra together with the fit for **S-InGa** before and after catalysis respectively.

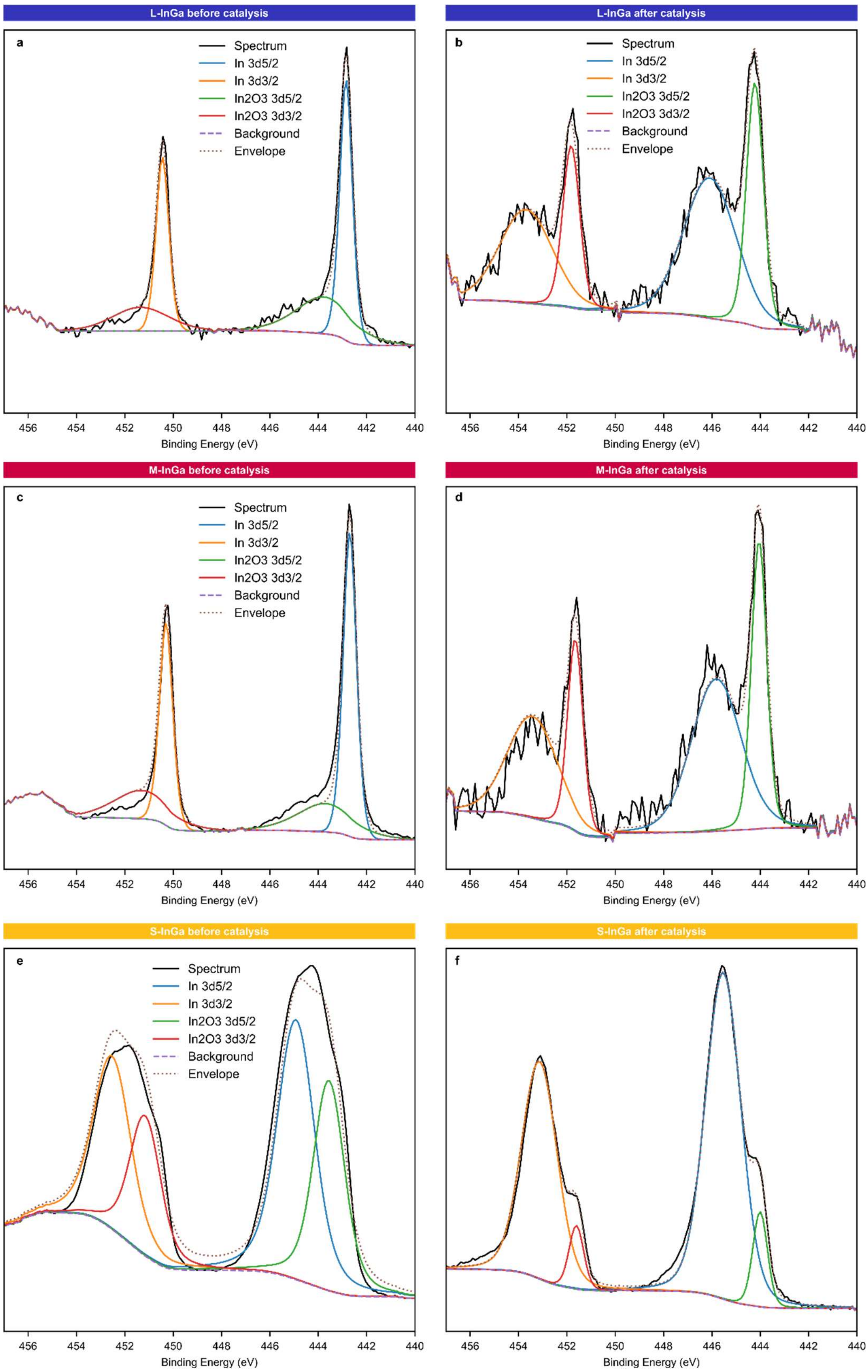

Figure S28 – XPS spectra of the In3d region of In-Ga NPs before and after CO2RR at room-temperature. (a,b) XPS spectra together with the fit for **L-InGa** before and after catalysis respectively. **(c,d)** XPS spectra together with the fit for **M-InGa** before and after catalysis respectively. **(e,f)** XPS spectra together with the fit for **S-InGa** before and after catalysis respectively.

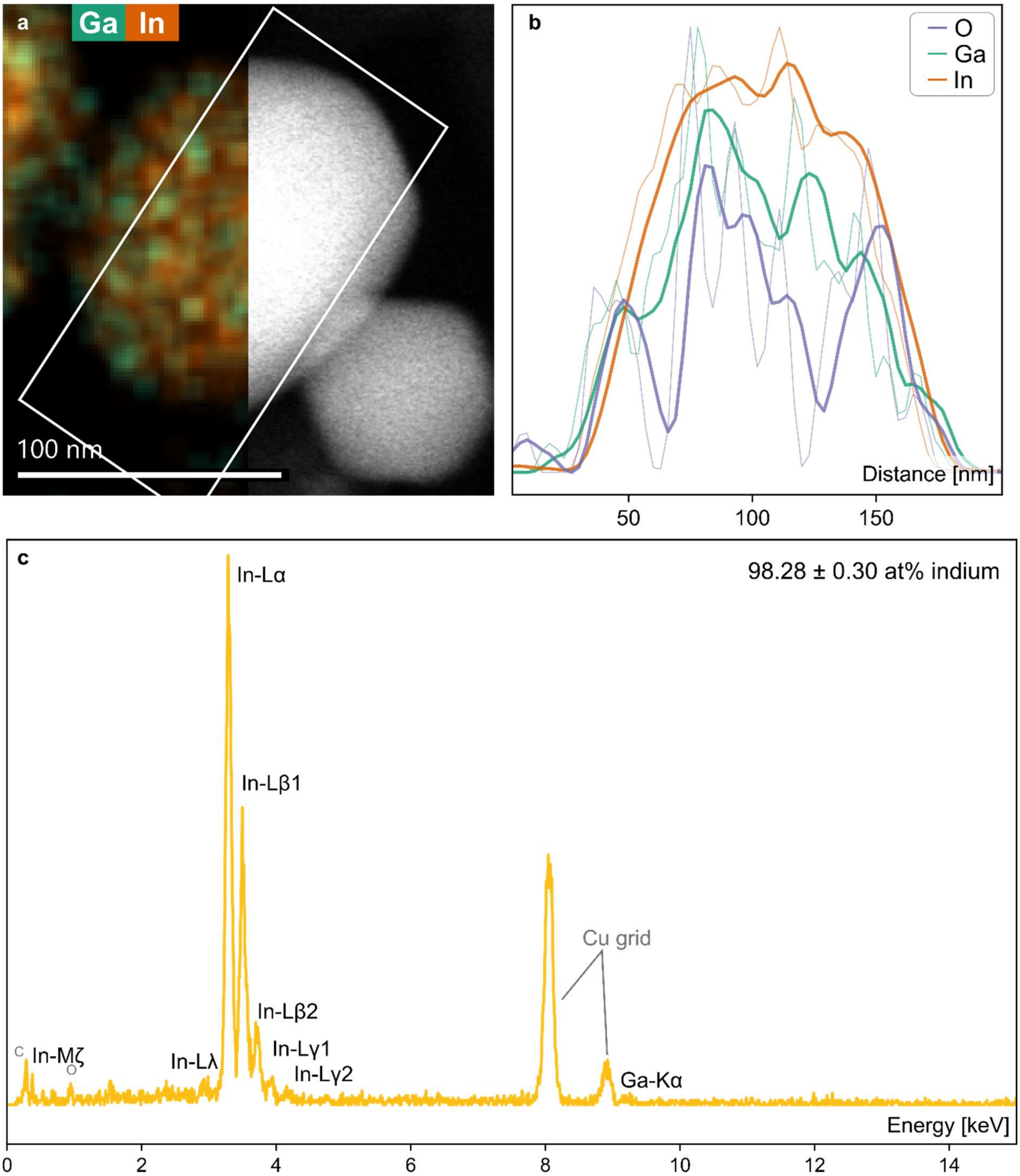

Figure S29 – HAADF STEM and EDXS of S-InGa after 1h at -0.7 V_{RHE}. (a) EDXS (green: gallium, orange: indium) and HAADF STEM of one NP. **(b)** Normalized line profile corresponding to the white rectangle area in **a**. **(c)** EDXS spectrum of the NP imaged in **a**.

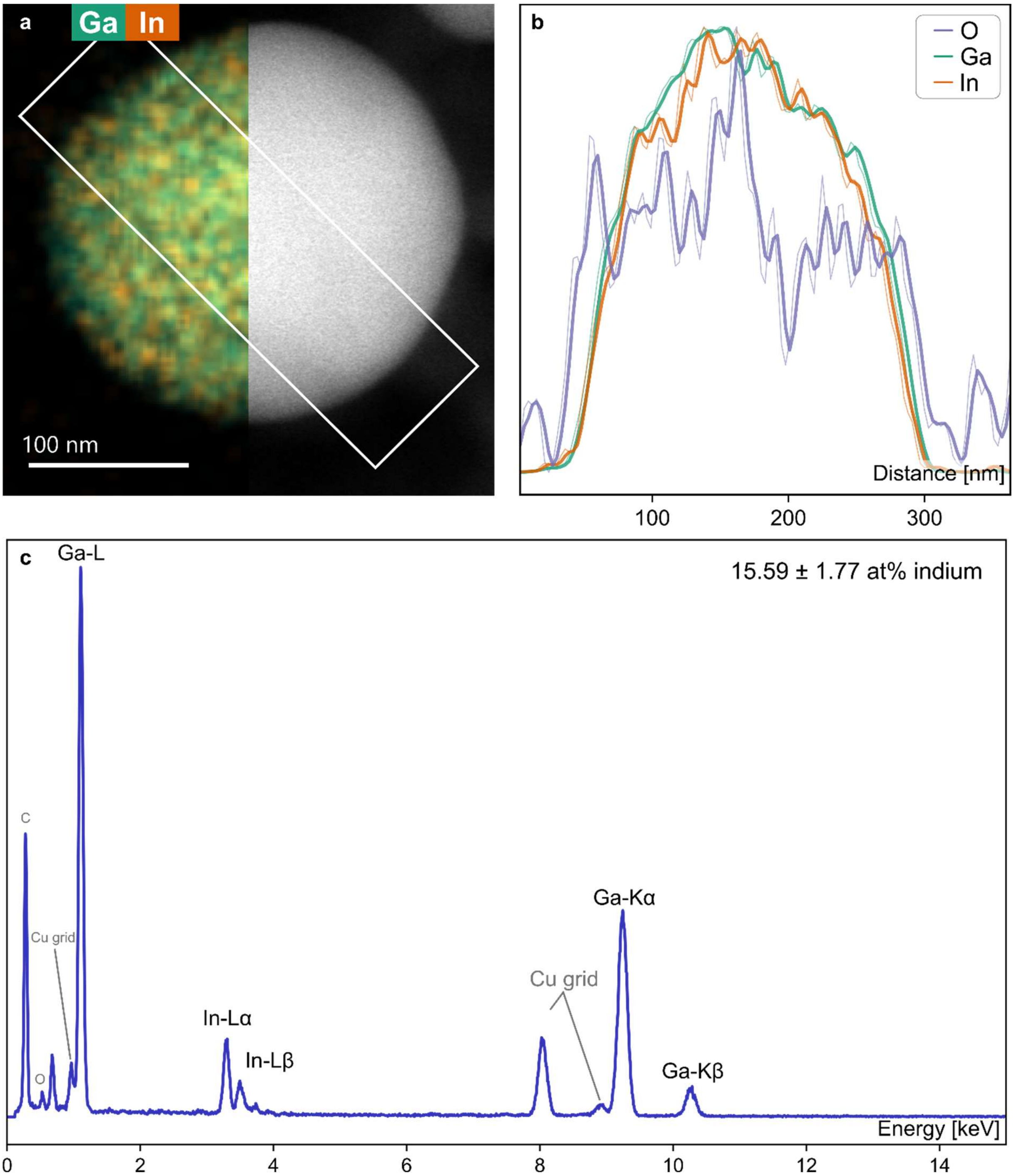

Figure S30 – HAADF STEM and EDXS of L-InGa after 1h at -0.7 V_{RHE}. (a) EDXS (green: gallium, orange: indium) and HAADF STEM of one NP. **(b) Normalized** line profile corresponding to the white rectangle area in **a**. **(c)** EDXS spectrum of the NP imaged in **a**.

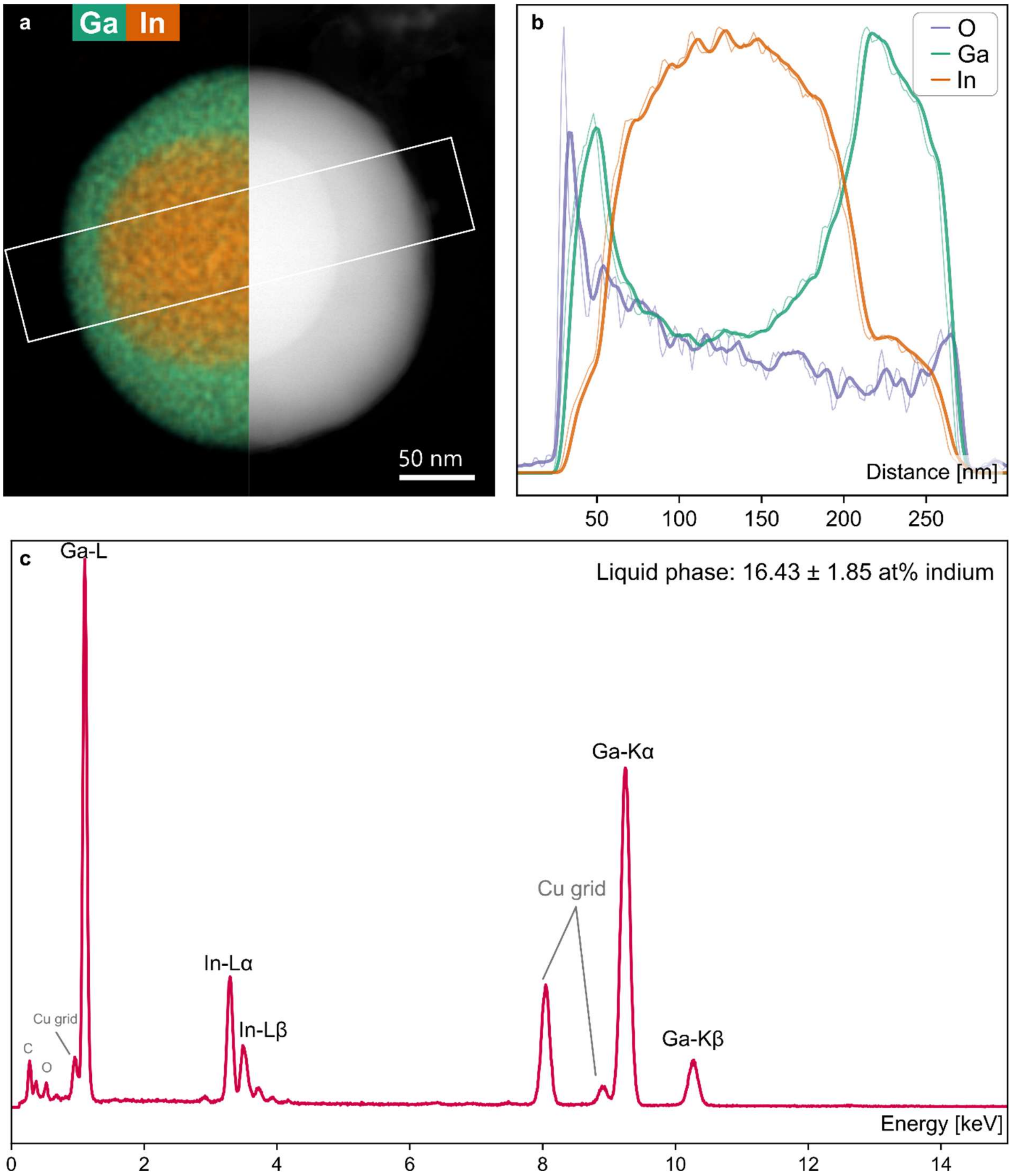

Figure S31 – HAADF STEM and EDXS of M-InGa after 1h at -0.7 V_{RHE}. (a) EDXS (green: gallium, orange: indium) and HAADF STEM of one NP. **(b) Normalized** line profile corresponding to the white rectangle area in **a**. **(c)** EDXS spectrum of the NP imaged in **a**.

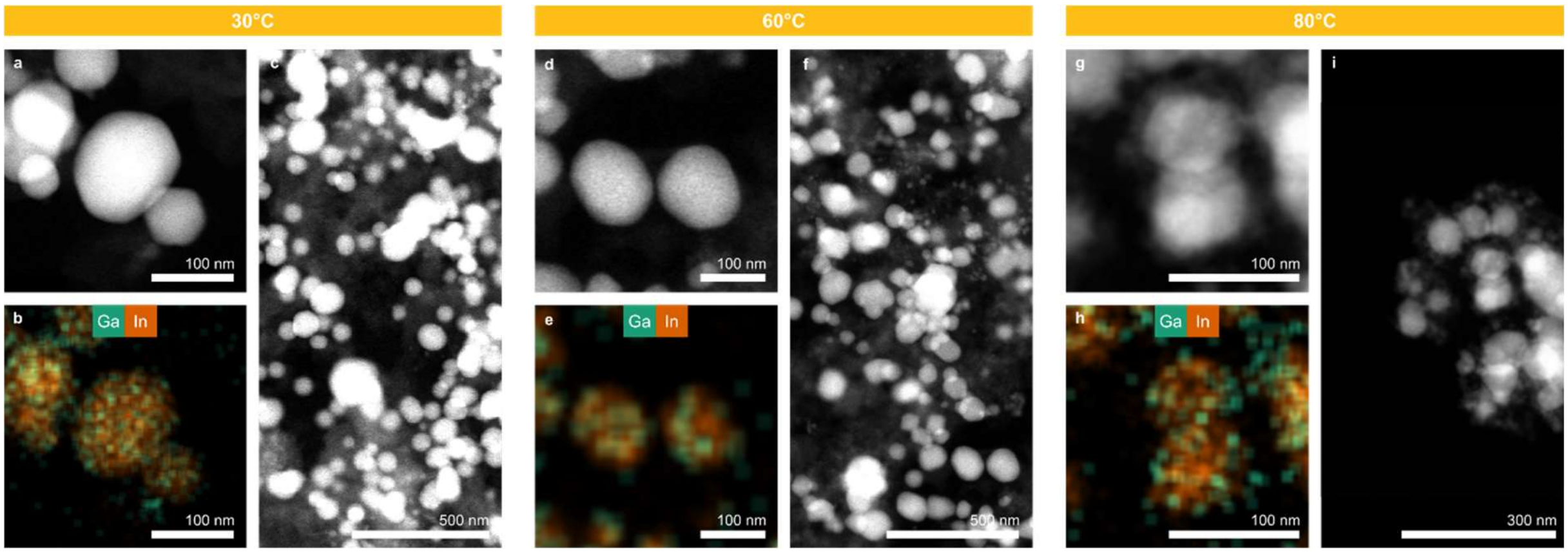

Figure S32 – HAADF-STEM images of S-InGa after 1h of catalysis at different temperature. (a-c) at 30°C. **(d-f)** at 60°C. **(g-i)** at 80°C. The experiment was run at -0.7V_{RHE}.

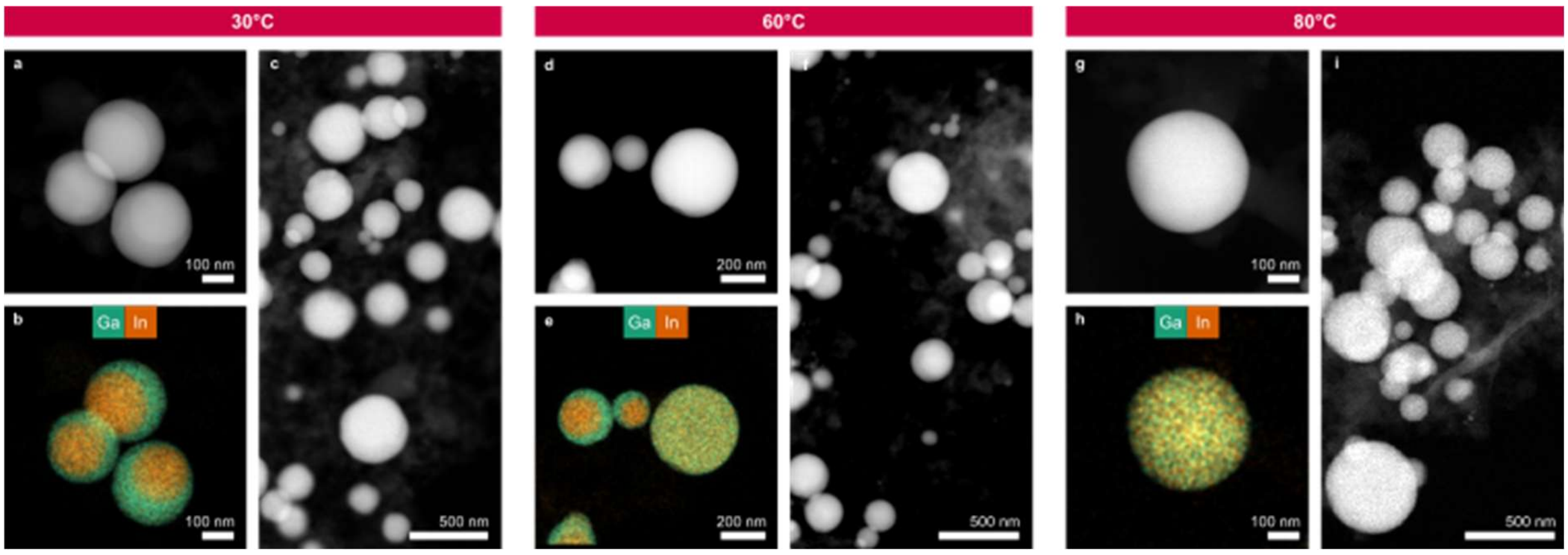

Figure S33 – HAADF-STEM images of M-InGa after 1h of catalysis at different temperature. (a-c) at 30°C. **(d-f)** at 60°C. **(g-i)** at 80°C. The experiment was run at -0.7V_{RHE}.

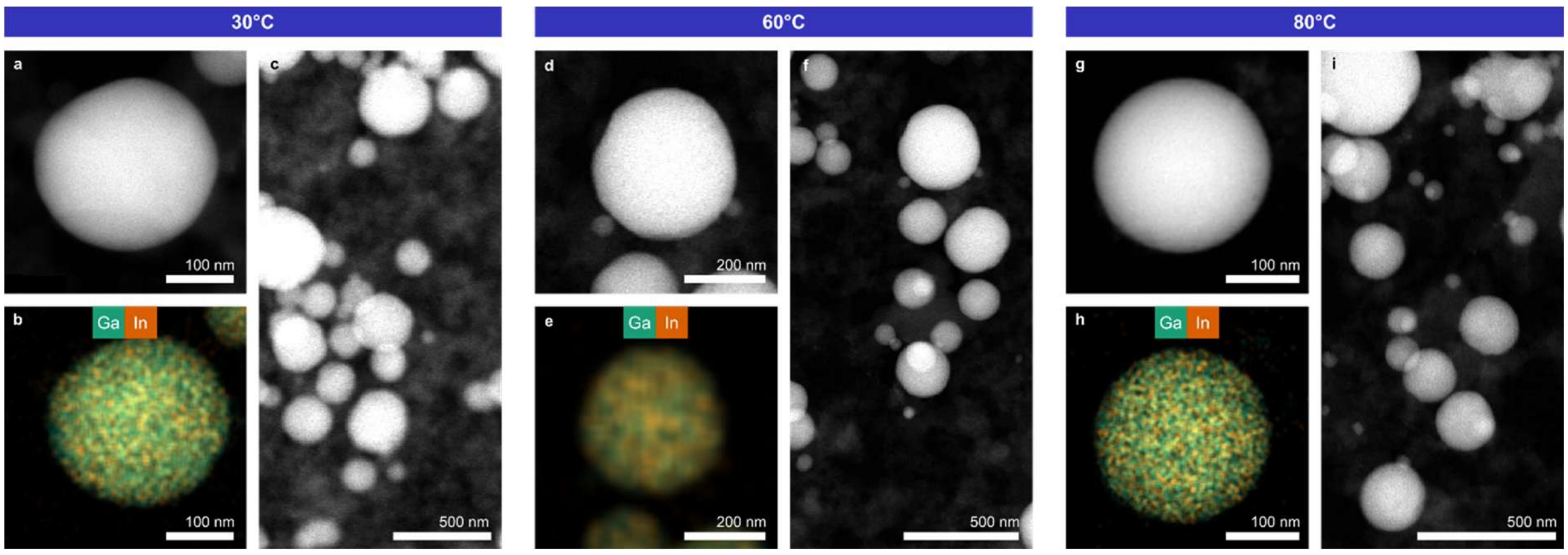

Figure S34 – HAADF-STEM images of L-InGa after 1h of catalysis at different temperature. (a-c) at 30°C. **(d-f)** at 60°C. **(g-i)** at 80°C. The experiment was run at -0.7V_{RHE}.

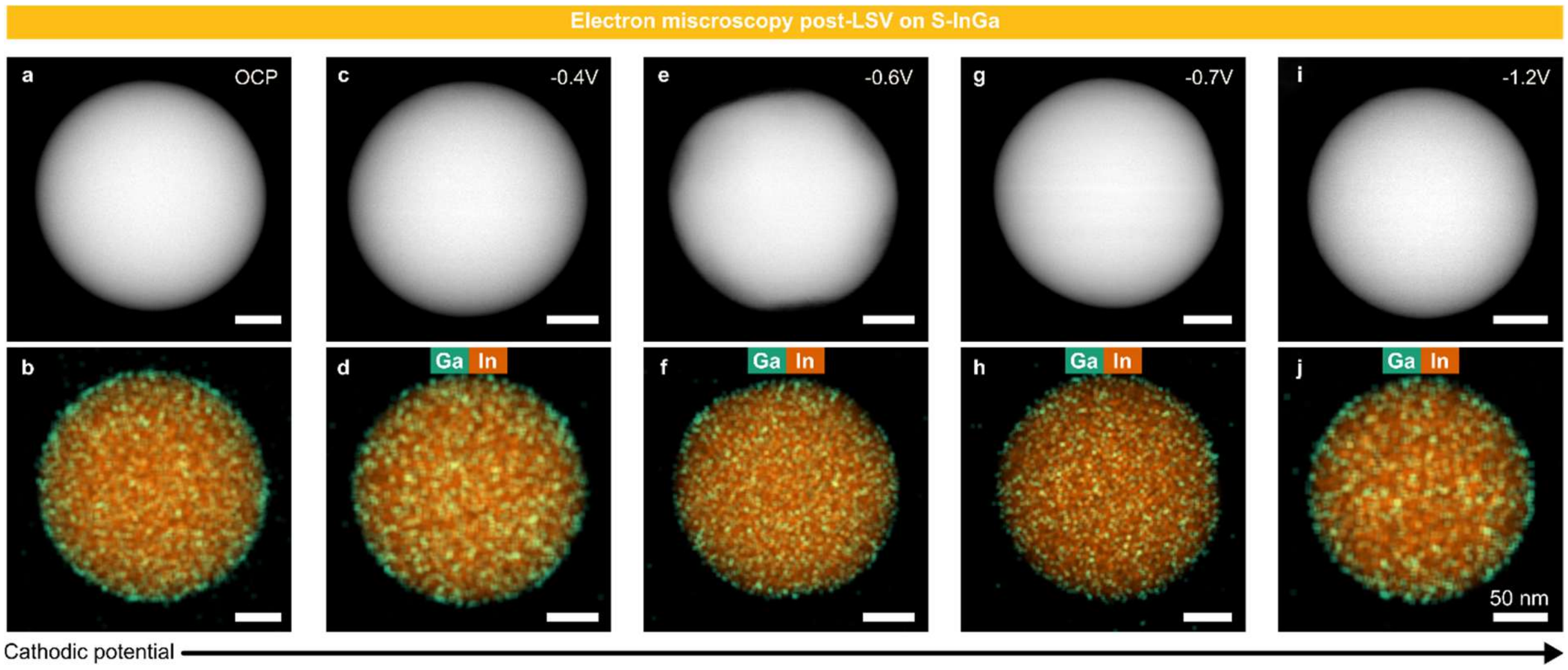

Figure S35 – HAADF-STEM images and EDXS of S-InGa post-LSV. Images and corresponding elemental maps of **S-InGa** after LSV from **(a,b)** E_{OC} to **(c,d)** -0.4 V_{RHE}, **(e,f)** -0.6 V_{RHE}, **(g,h)** -0.7 V_{RHE} and **(i,j)** -1.2 V_{RHE}. No structural change is observed.

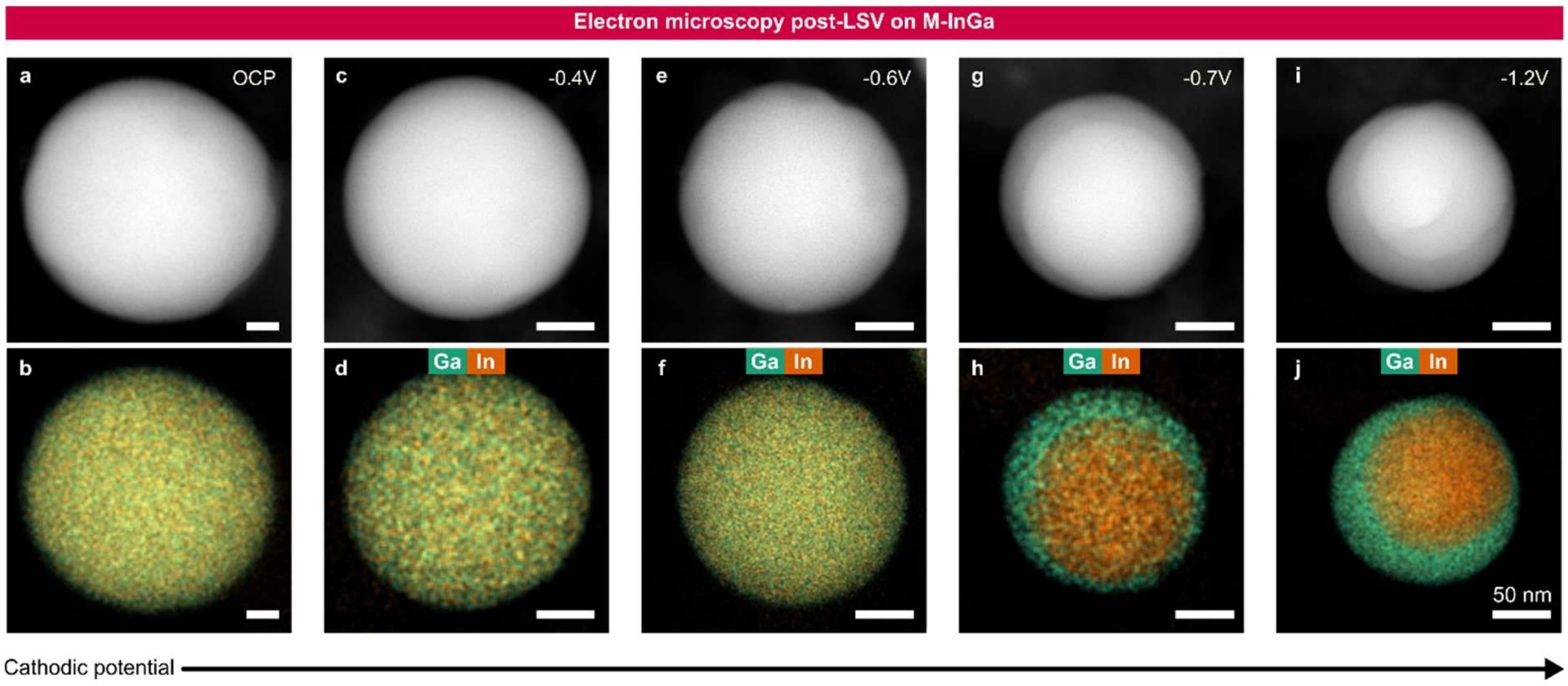

Figure S36 – HAADF-STEM images and EDXS of M-InGa post-LSV. Images and corresponding elemental maps of **S-InGa** after LSV from **(a,b)** E_{OC} to **(c,d)** -0.4 V_{RHE}, **(e,f)** -0.6 V_{RHE}, **(g,h)** -0.7 V_{RHE} and **(i,j)** -1.2 V_{RHE}. Between -0.6 V_{RHE} and -0.7 V_{RHE}, The NPs undergo a phase segregation to a solid-liquid core-shell structure.

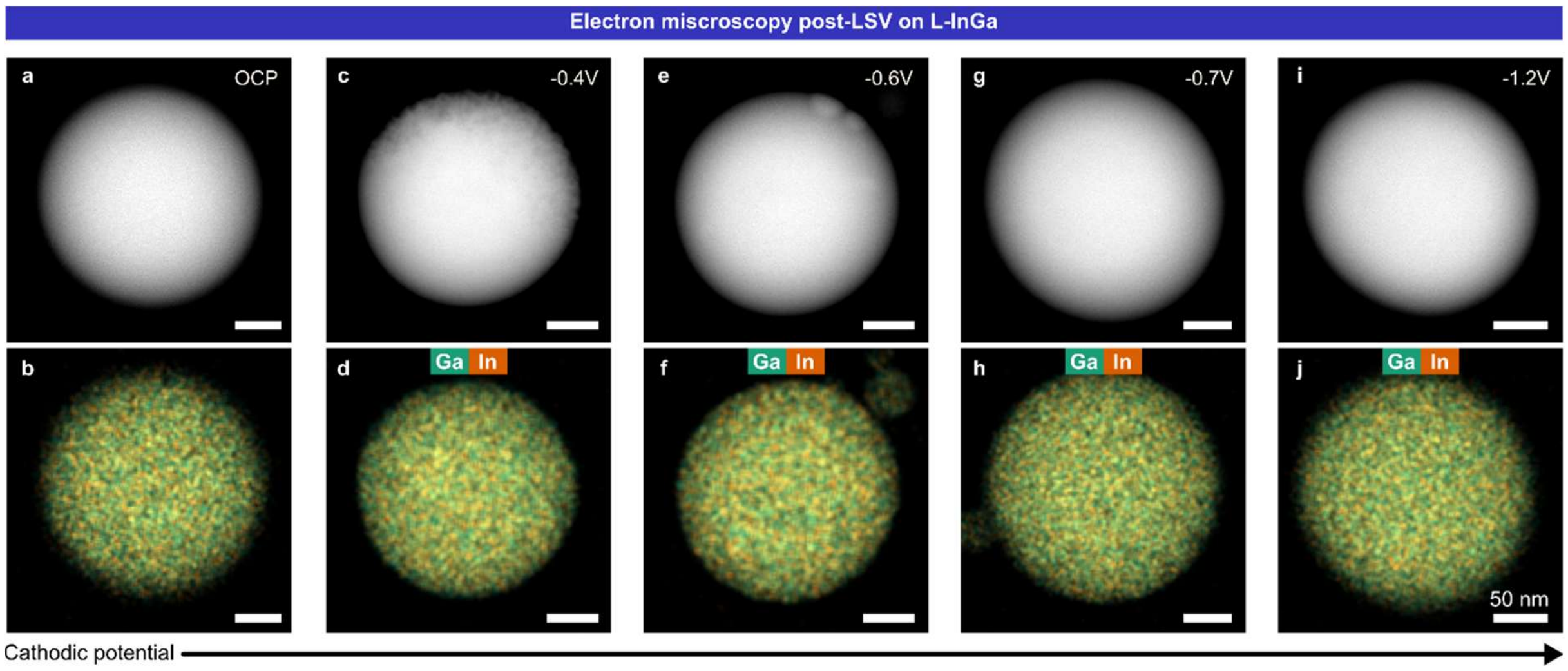

Figure S37 – HAADF-STEM images and EDXS of S-InGa post-LSV. Images and corresponding elemental maps of **S-InGa** after LSV from **(a,b)** E_{OC} to **(c,d)** -0.4 V_{RHE}, **(e,f)** -0.6 V_{RHE}, **(g,h)** -0.7 V_{RHE} and **(i,j)** -1.2 V_{RHE}. No structural change is observed.

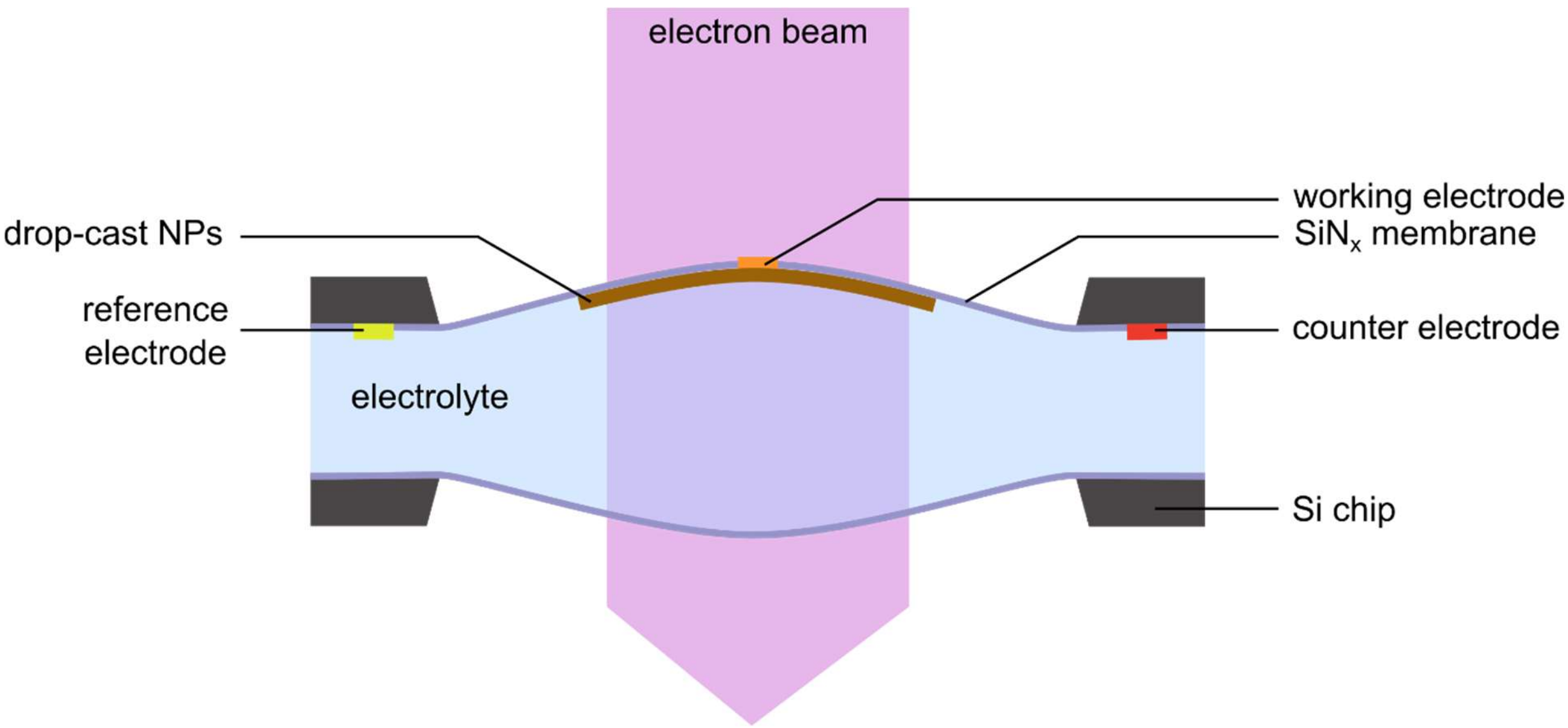

Figure S38 – Schematic of the components of the ecLPTEM set up.

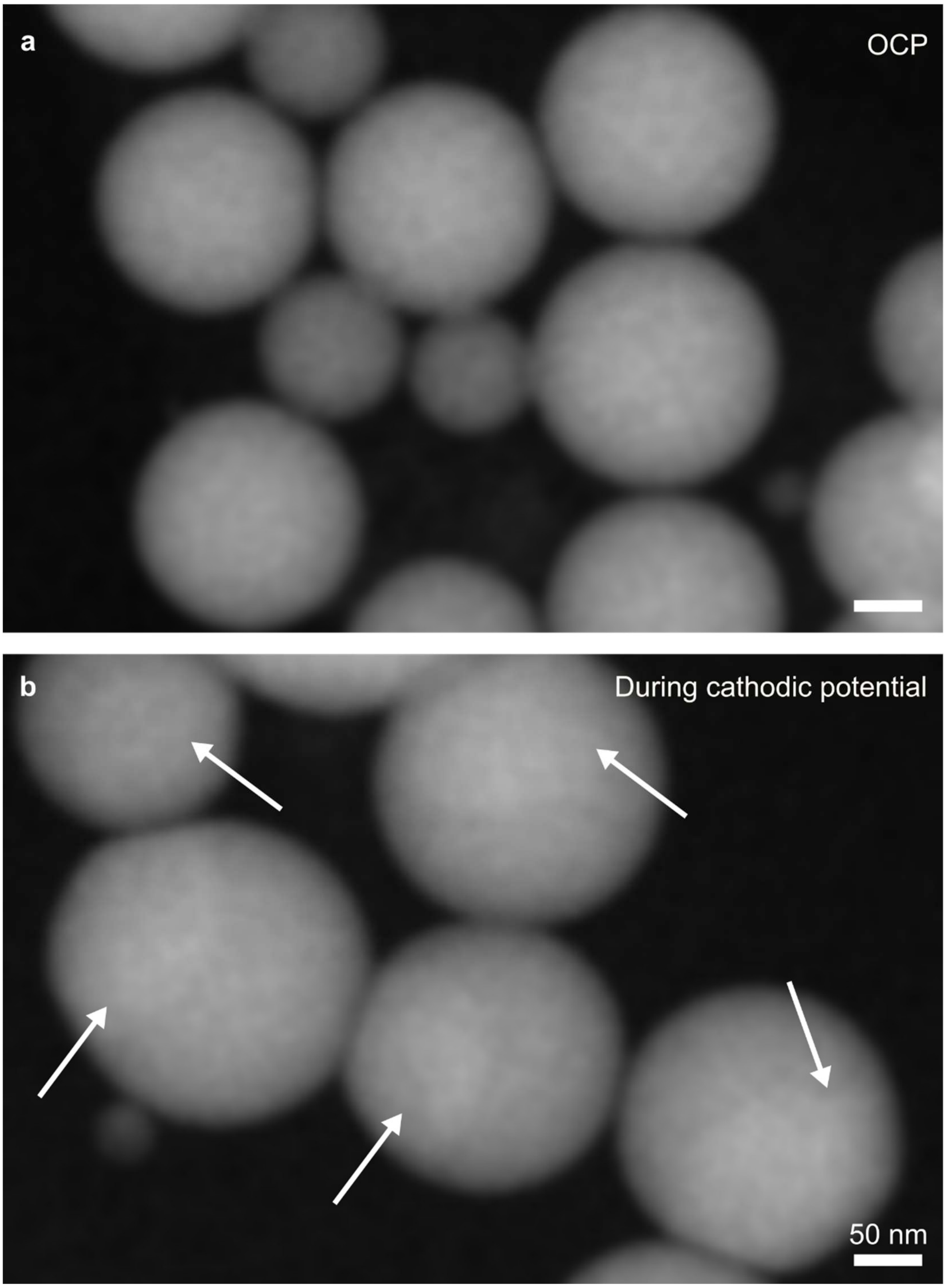

Figure S39 – HAADFT-STEM image of M-InGa during catalysis in the ecLPTEM cell. HAADF-STEM image of the NPs **(a)** at OCP and **(b)** 2 min after the beginning of the chronoamperometry experiment at -0.7 V_{RHE}. The white arrows indicate the formation of a bright core within each NP.

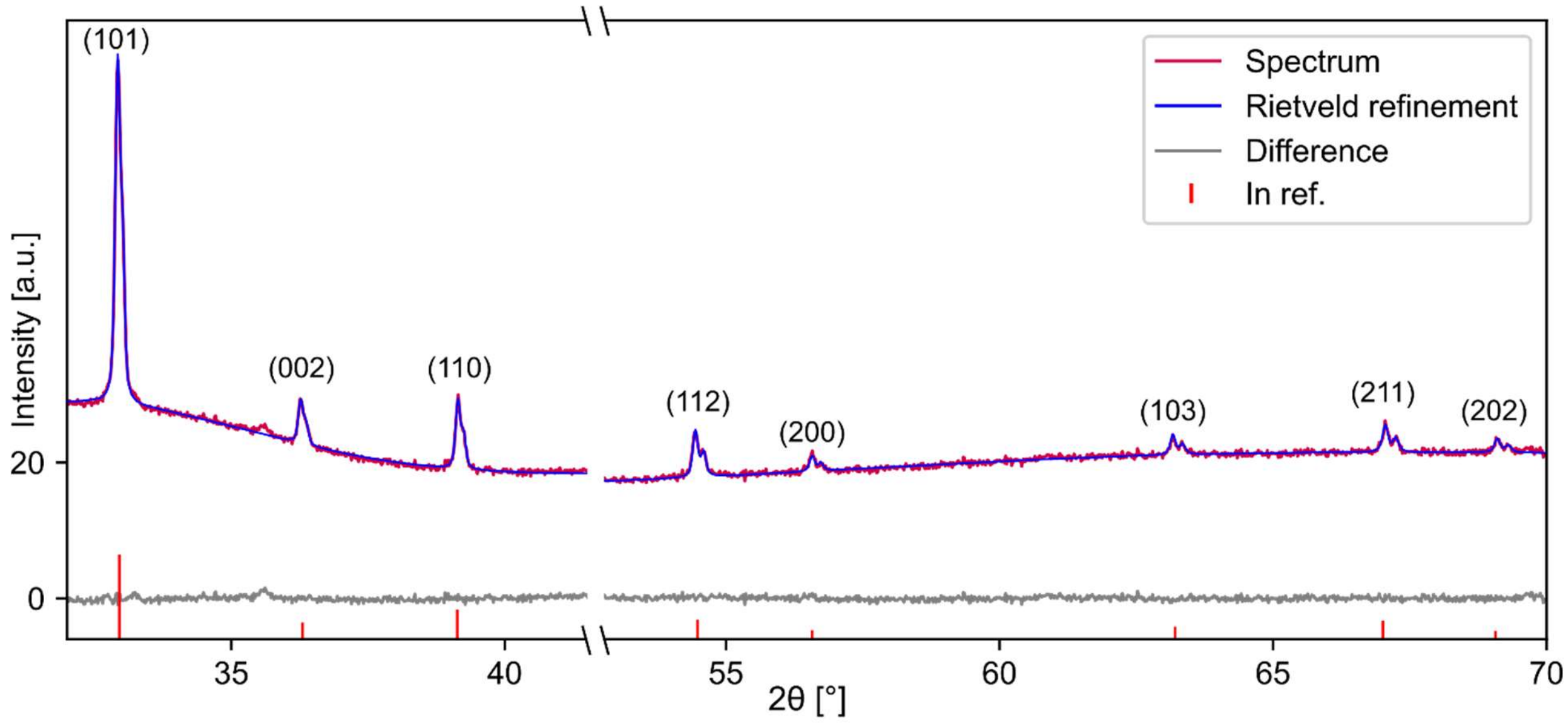

Figure S40 – XRD pattern of M-InGa at room temperature after immersion in liquid nitrogen and Rietveld refinement. Each diffraction peak appears as a doublet, corresponding to Cu-Kα_1/Kα_2 splitting of the X-ray anode's emission profile of the X-ray anode.

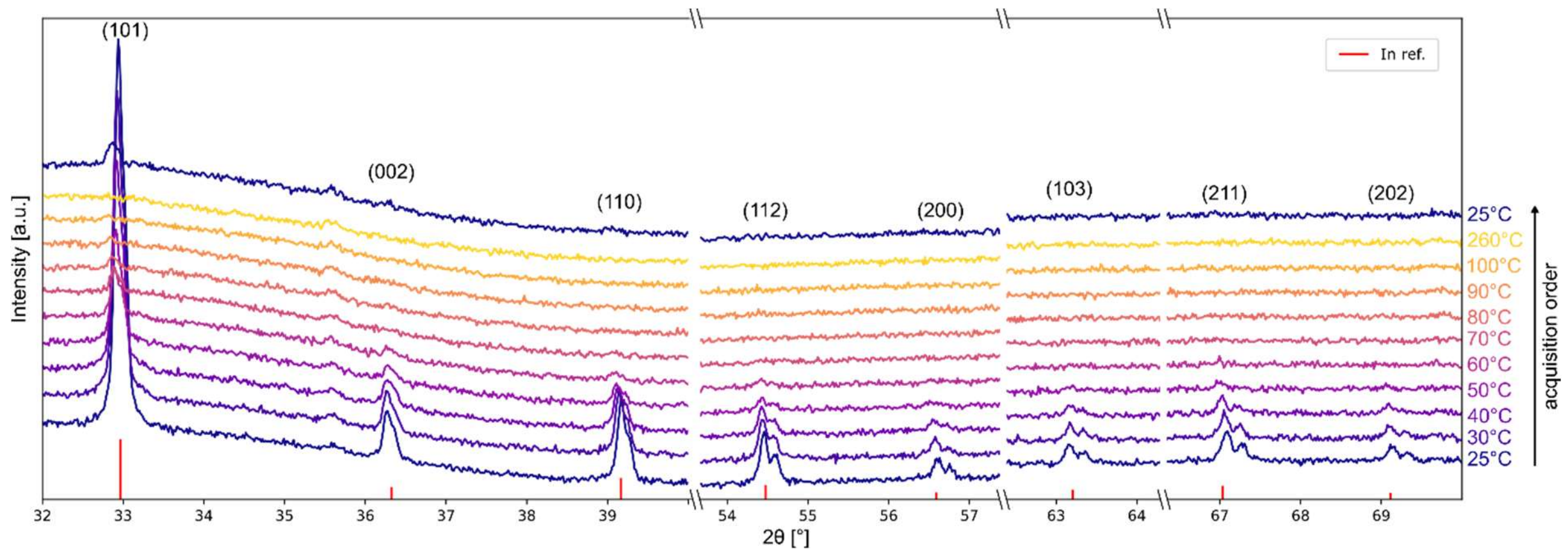

Figure S41 – XRD patterns collected while heating M-InGa from room temperature to 260°C. The NPs were immersed in liquid nitrogen prior to the experiment in order to start with the phase segregated structure. Each diffraction peak appears as a doublet, corresponding to Cu-Kα_1/Kα_2 splitting of the X-ray anode's emission profile of the X-ray anode.

Note S3 – Indium quantification in the liquid phase at increasing temperature through electron microscopy.

To observe the evolution of the core size and the composition of the liquid phase of **M-InGa** with temperature, an in-situ electron microscopy experiment was performed. The suspension of as-synthesized NPs was immersed in liquid nitrogen to trigger the phase segregation, before drop-casting it on the MEMS heating chip.

As the NP structure consists of a solid core floating in a liquid metal droplet, the core can move inside the NP, making the elemental mapping more challenging. Therefore, we selected three regions corresponding to four NPs whose cores were not moving at room temperature (**Figure S42-44**). We monitored these regions with EDXS at the following temperatures: room temperature, 40°C, 50°C, 60°C, 70°C, 80°C, 90°C, 100°C, 150°C (only for Particle 2) and back to room temperature (**Figure S45-50**).

As discussed on the corresponding method description, for the temperatures at which the core remained immobile or was fully dissolved, the quantification was done by first segmenting the region of interest (*i.e.* the liquid phase) with Scikit-image[3] before using a robust multiscale Bayesian (RMB) approach for quantification (**Figure S45, 46**).[2] For the temperatures at which the cores were moving throughout acquisition, likely due to thermal Brownian motion, the liquid phase was selected frame by frame, and the sum of the selected pixel was used to quantify the resulting spectrum (**Figure S47-50**).

At 60°C, we induced a temperature drop to see the effect on the core size, and it immediately led to an immediate increase in the core size of the NPs (**Figure S51**). When going back to 60°C, the core size decreased again, indicating the reversibility of the dissolution process and evidencing the equilibrium between the solubilized indium and the indium in the solid core.

Finally, an interesting observation was made at 90°C. At this temperature, a core was still visible for the four selected NPs (**Figure S48** and **Figure S50**). Yet, most of the NPs surrounding the regions scanned with EDXS were fully liquid at this temperature (**Figure S52**). Therefore, the temperature at which **M-InGa** is fully liquid might be overestimated based on electron microscopy, and the real temperature might be slightly lower. In any case, the overall trend is the same with XRD and EDXS, highlighting the rapid dissolution of the indium core within liquid gallium NPs.

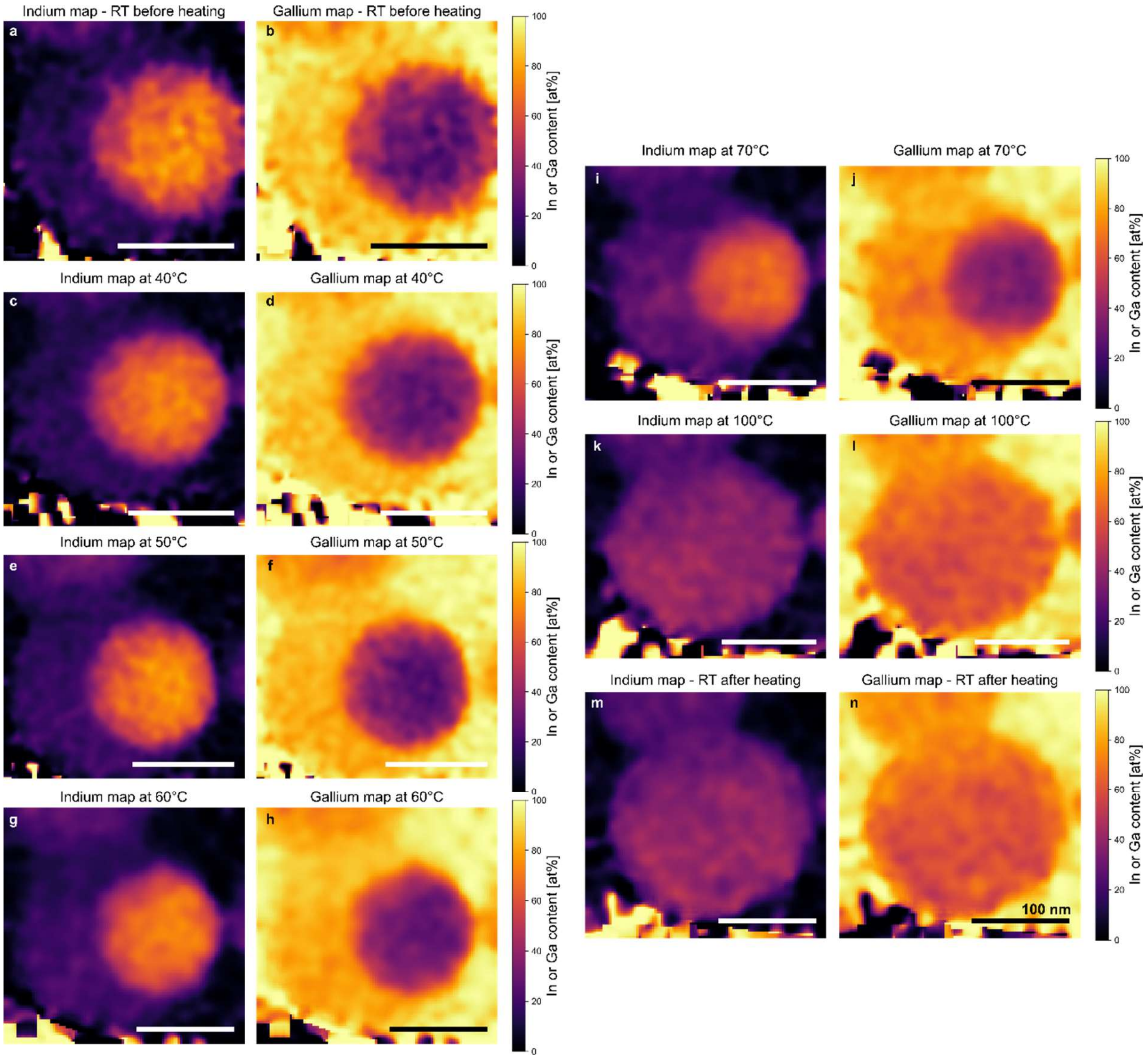

Figure S42 – Gallium and indium elemental EDXS maps at increasing temperatures for Particle 1. (a,b) Maps at room temperature, before the start of the heating experiment (left: indium map; right: gallium map). **(c,d)** Maps at 40°C (left: indium map; right: gallium map). **(e,f)** Maps at 50°C (left: indium map; right: gallium map). **(g,h)** Maps at 60°C (left: indium map; right: gallium map). **(i,j)** Maps at 70°C (left: indium map; right: gallium map). **(k,l)** Maps at 100°C (left: indium map; right: gallium map). **(m,n)** Maps at room temperature, after the heating experiment (left: indium map; right: gallium map). Scale bar is 100 nm.

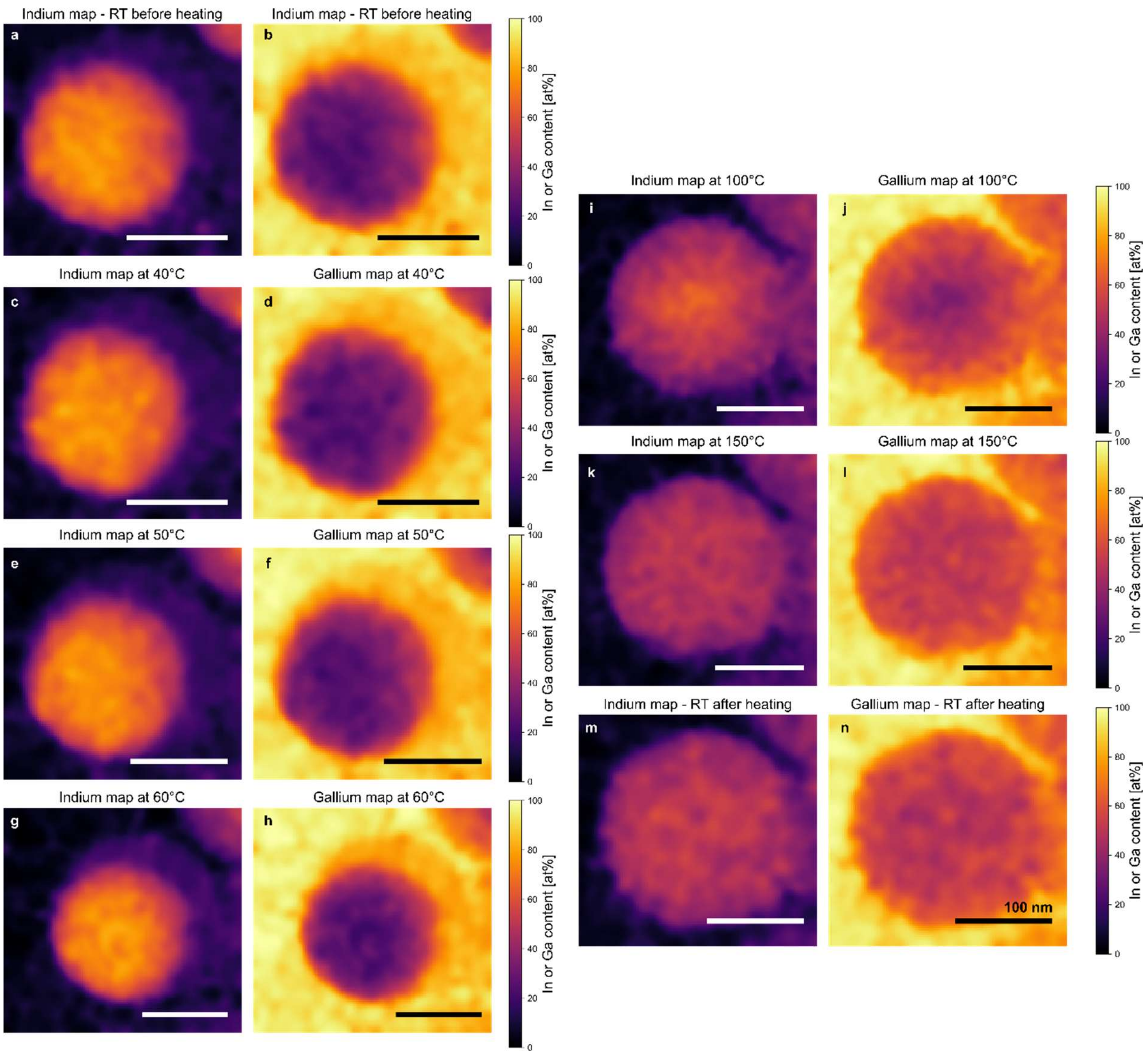

Figure S43 – Gallium and indium elemental EDXS maps at increasing temperatures for Particle 2. (a,b) Maps at room temperature, before the start of the heating experiment (left: indium map; right: gallium map). **(c,d)** Maps at 40°C (left: indium map; right: gallium map). **(e,f)** Maps at 50°C (left: indium map; right: gallium map). **(g,h)** Maps at 60°C (left: indium map; right: gallium map). **(i,j)** Maps at 100°C (left: indium map; right: gallium map). **(k,l)** Maps at 150°C (left: indium map; right: gallium map).). **(m,n)** Maps at room temperature, after the heating experiment (left: indium map; right: gallium map). Scale bar is 100 nm.

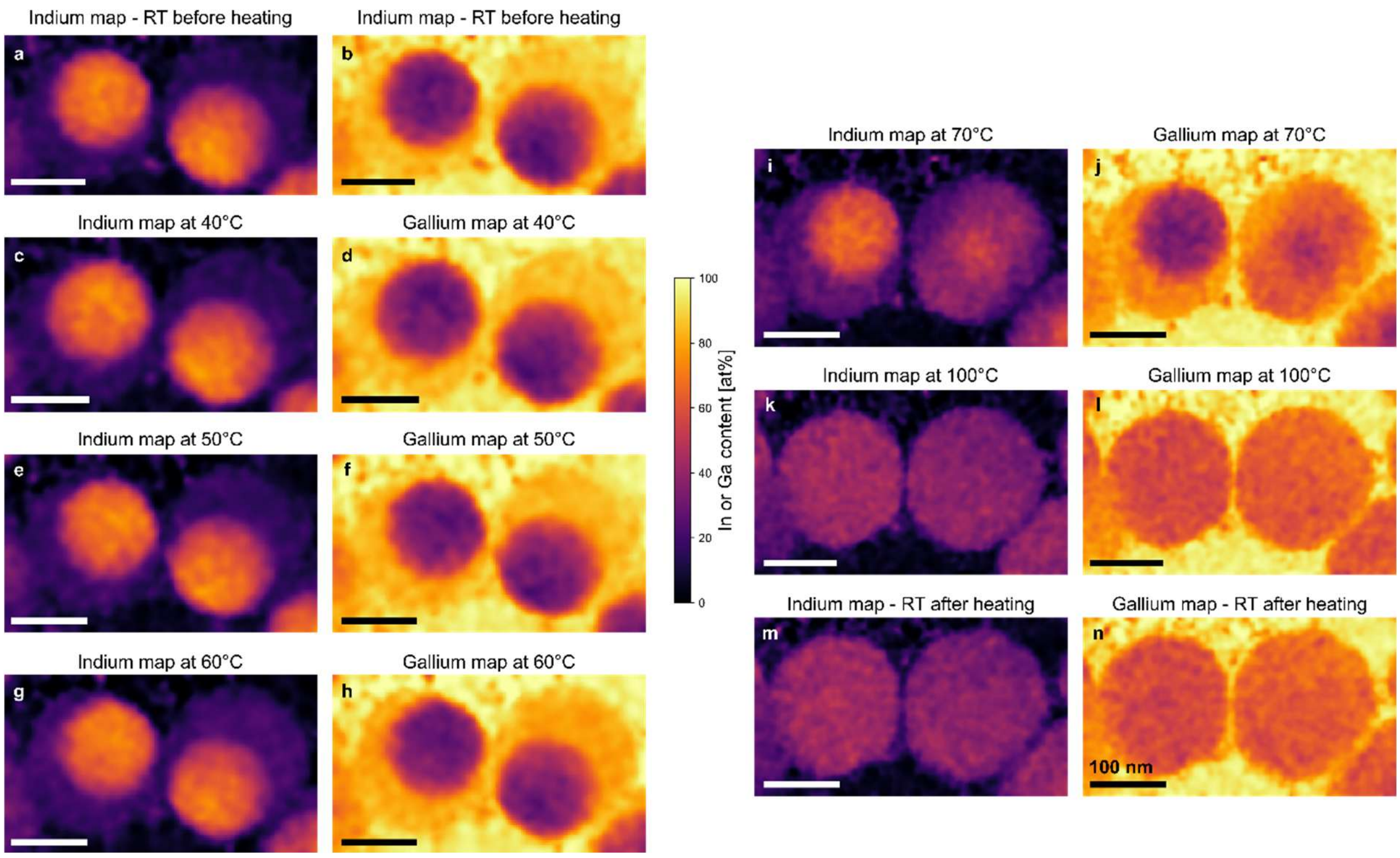

Figure S44 – Gallium and indium elemental EDXS maps at increasing temperatures for Particles 3. (a,b) Maps at room temperature, before the start of the heating experiment (left: indium map; right: gallium map). **(c,d)** Maps at 40°C (left: indium map; right: gallium map). **(e,f)** Maps at 50°C (left: indium map; right: gallium map). **(g,h)** Maps at 60°C (left: indium map; right: gallium map). **(i,j)** Maps at 70°C (left: indium map; right: gallium map). **(k,l)** Maps at 100°C (left: indium map; right: gallium map). **(m,n)** Maps at room temperature, after the heating experiment (left: indium map; right: gallium map). Scale bar is 100 nm. At 70°C, the particle on the right exhibits core motion, which results in attenuated contrast in the corresponding indium map.

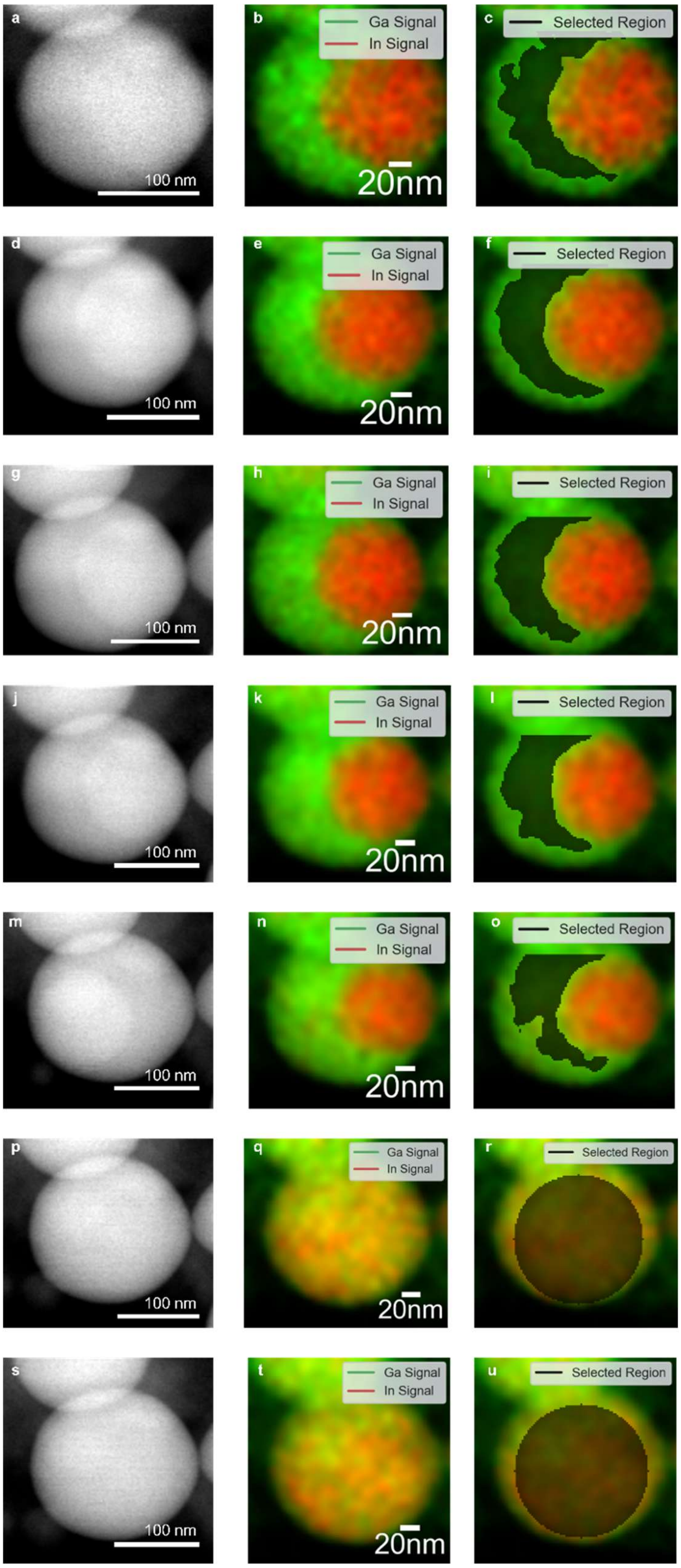

Figure S45 – HAADF-STEM images and elemental maps (green: Ga, red: In) showing the selected area for quantifying indium in the liquid phase of Particle 1 at temperatures where the core remains rather stationary. (a,c) At room temperature before the experiment. **(d,f)** At 40°C. **(g,i)** At 50°C. **(j,l)** At 60°C. **(m,o)** At 70°C. **(p,r)** At 100°C. **(s,u)** Back to RT.

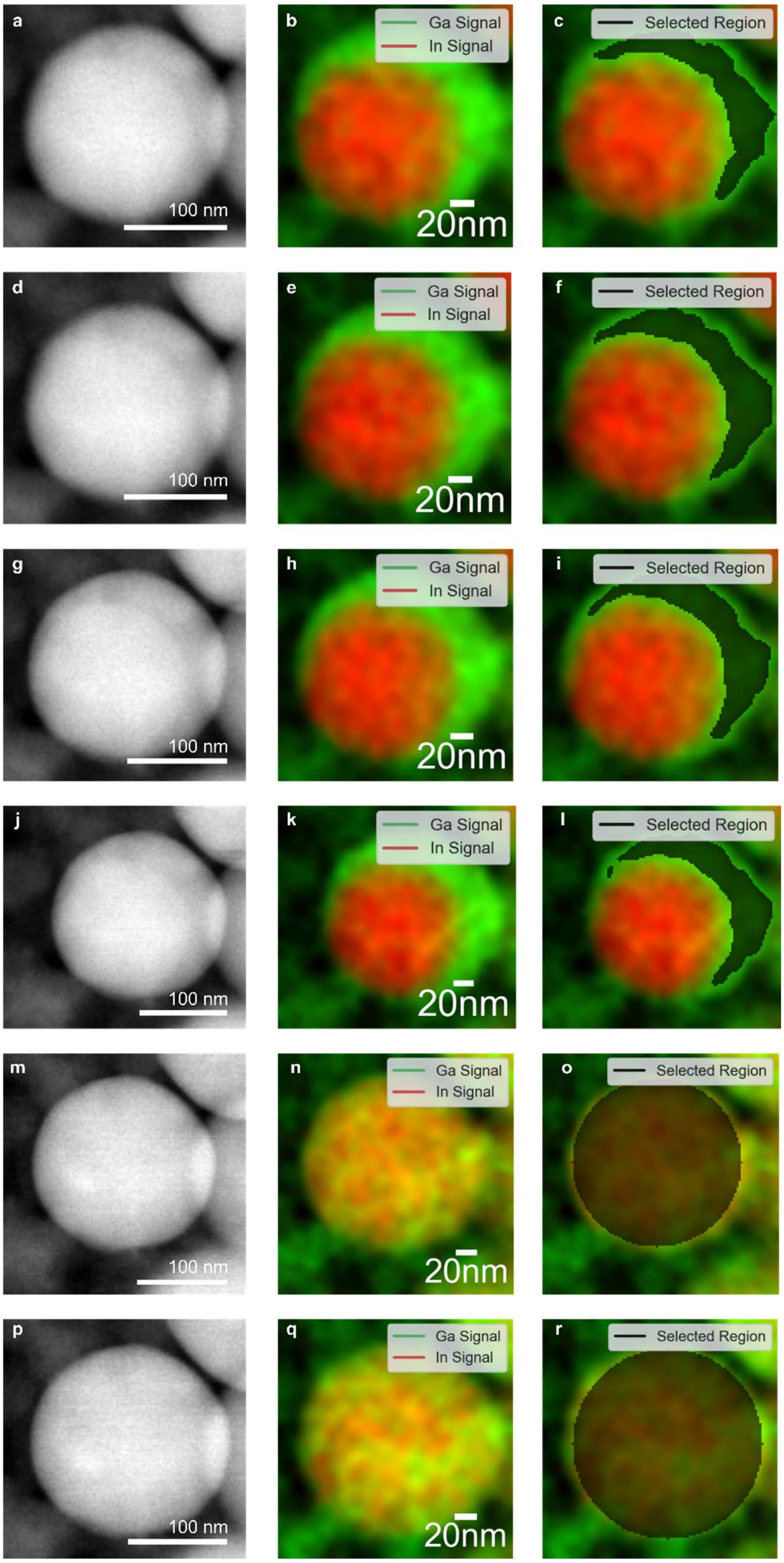

Figure S46 – HAADF-STEM images and elemental maps (green: Ga, red: In) showing the selected area for quantifying indium in the liquid phase of Particle 2 at temperatures where the core remains rather stationary. (a,c) At room temperature before the experiment. **(d,f)** At 40°C. **(g,i)** At 50°C. **(j,l)** At 60°C. **(m,o)** At 150°C. **(p,r)** Back to RT.

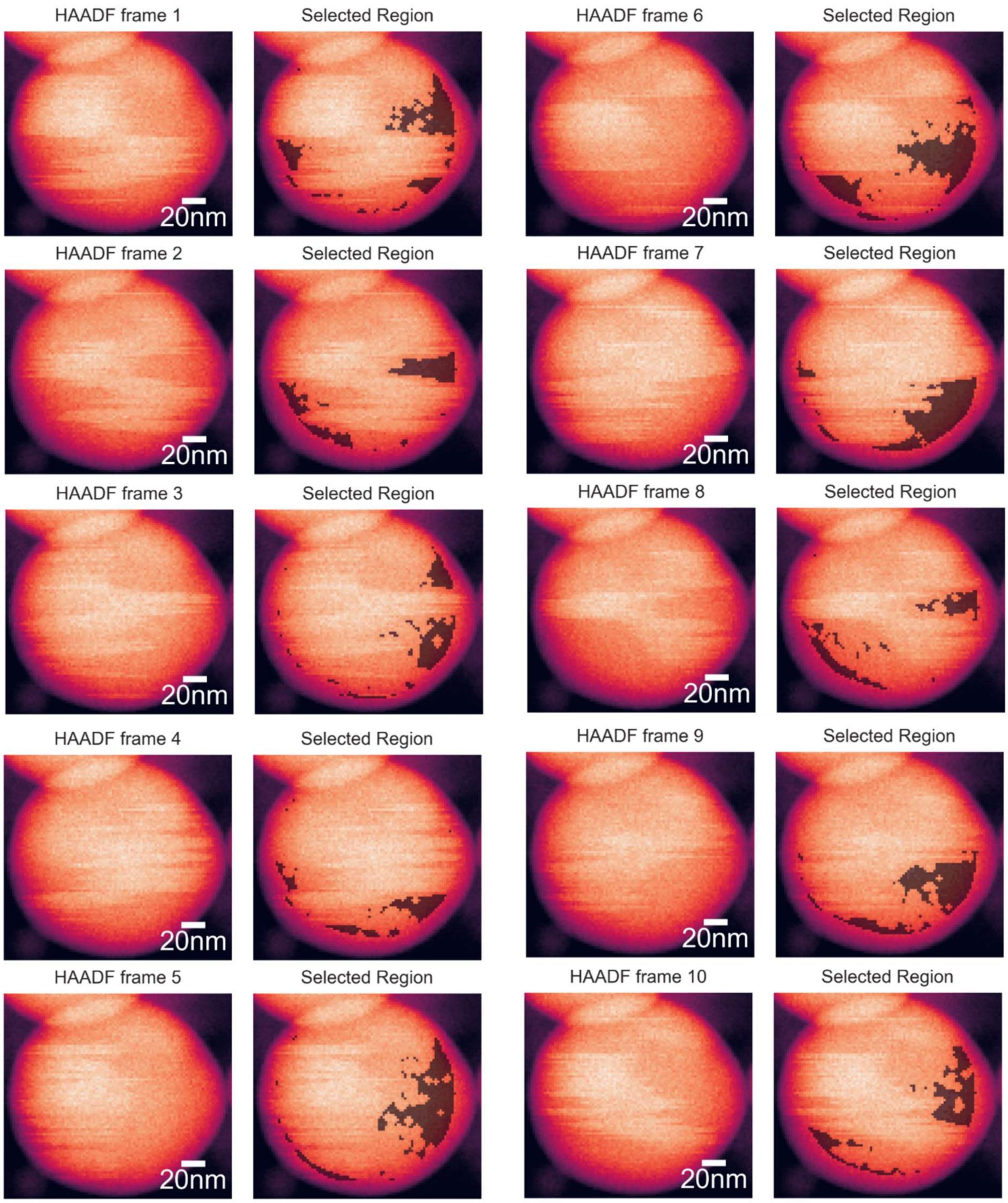

Figure S47 – Pixels selection for the indium quantification in the liquid phase at 80°C for Particle 1, where the core was continuously moving. The selection of the pixels for the first 10 frames is shown here. In total, 250 frames were acquired and analyzed.

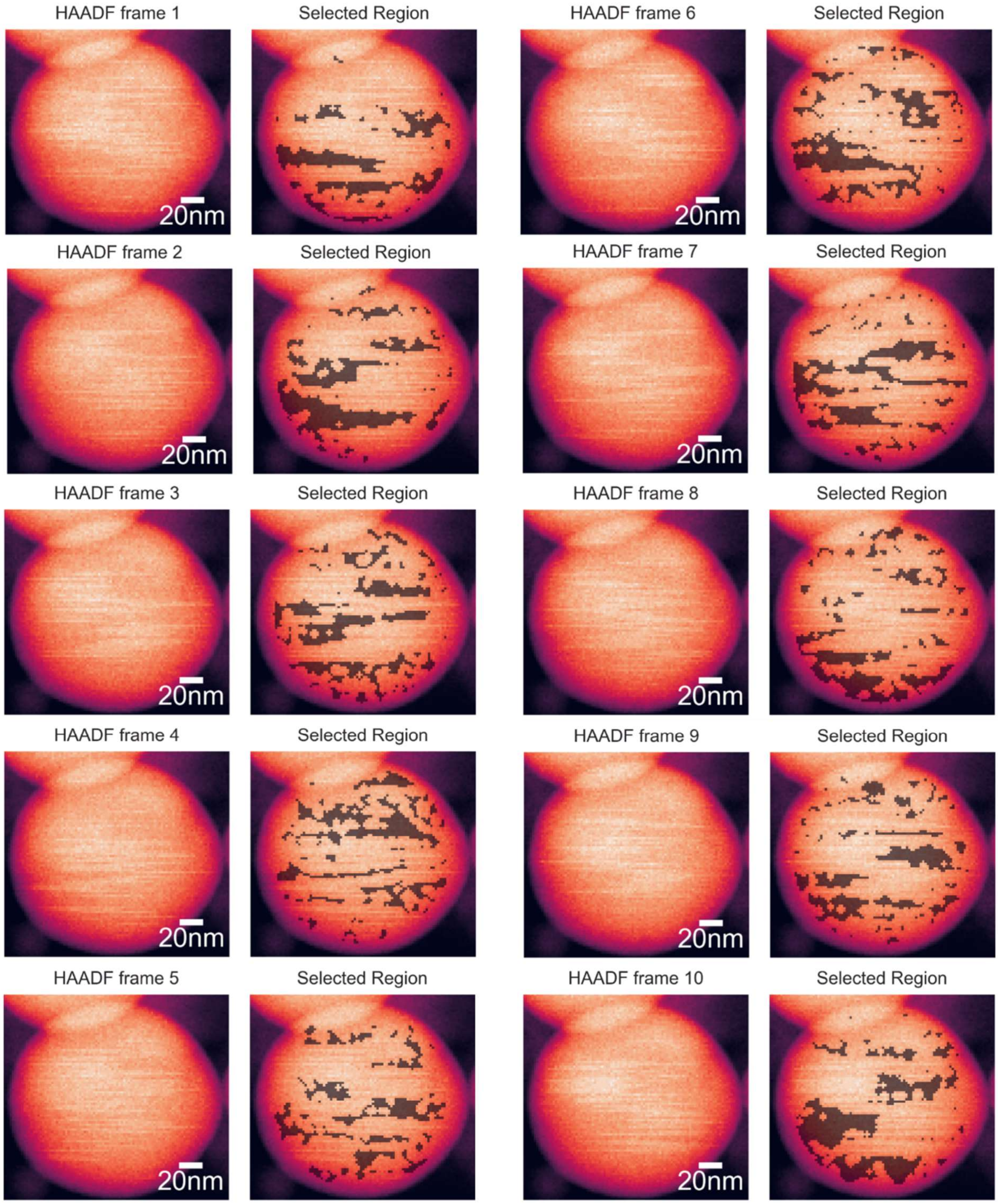

Figure S48 – Pixels selection for the indium quantification in the liquid phase at 90°C for Particle 1, where the core was continuously moving. The selection of the pixels for the first 10 frames is shown here. In total, 250 frames were acquired and analyzed.

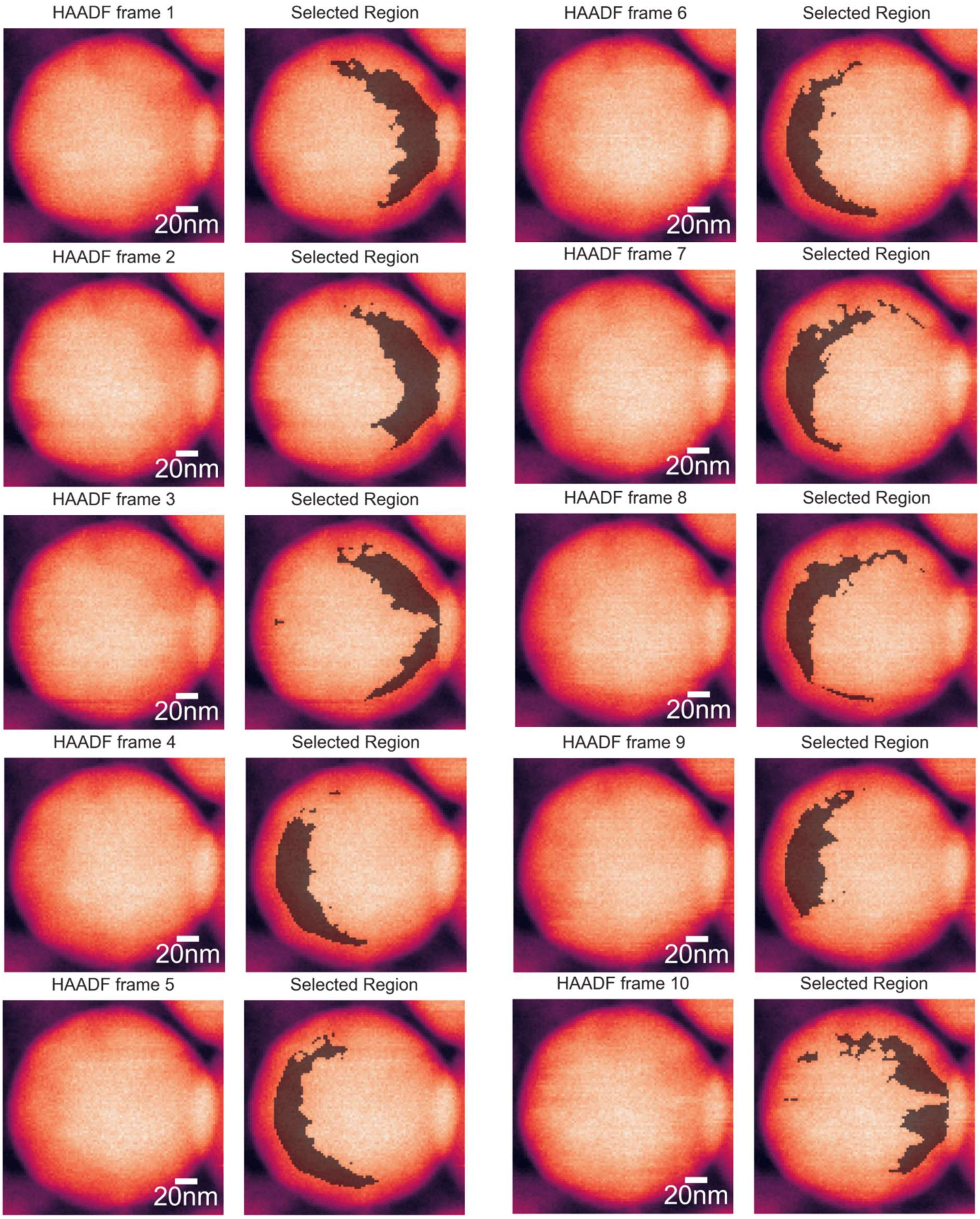

Figure S49 – Pixels selection for the indium quantification in the liquid phase at 80°C for Particle 2, where the core was continuously moving. The selection of the pixels for the first 10 frames is shown here. In total, 250 frames were acquired and analyzed.

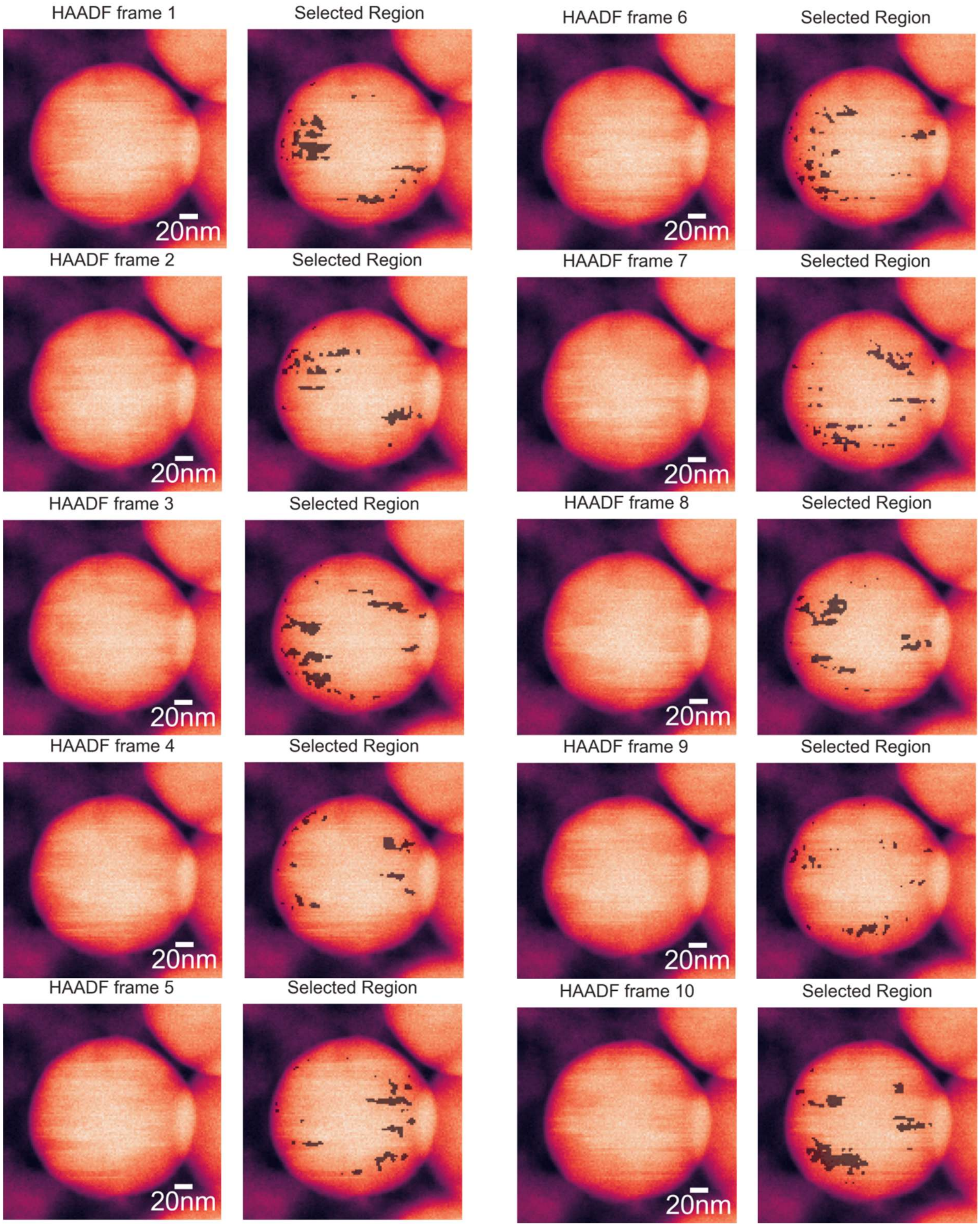

Figure S50 – Pixels selection for the indium quantification in the liquid phase at 90°C for Particle 2, where the core was continuously moving. The selection of the pixels for the first 10 frames is shown here. In total, 250 frames were acquired and analyzed.

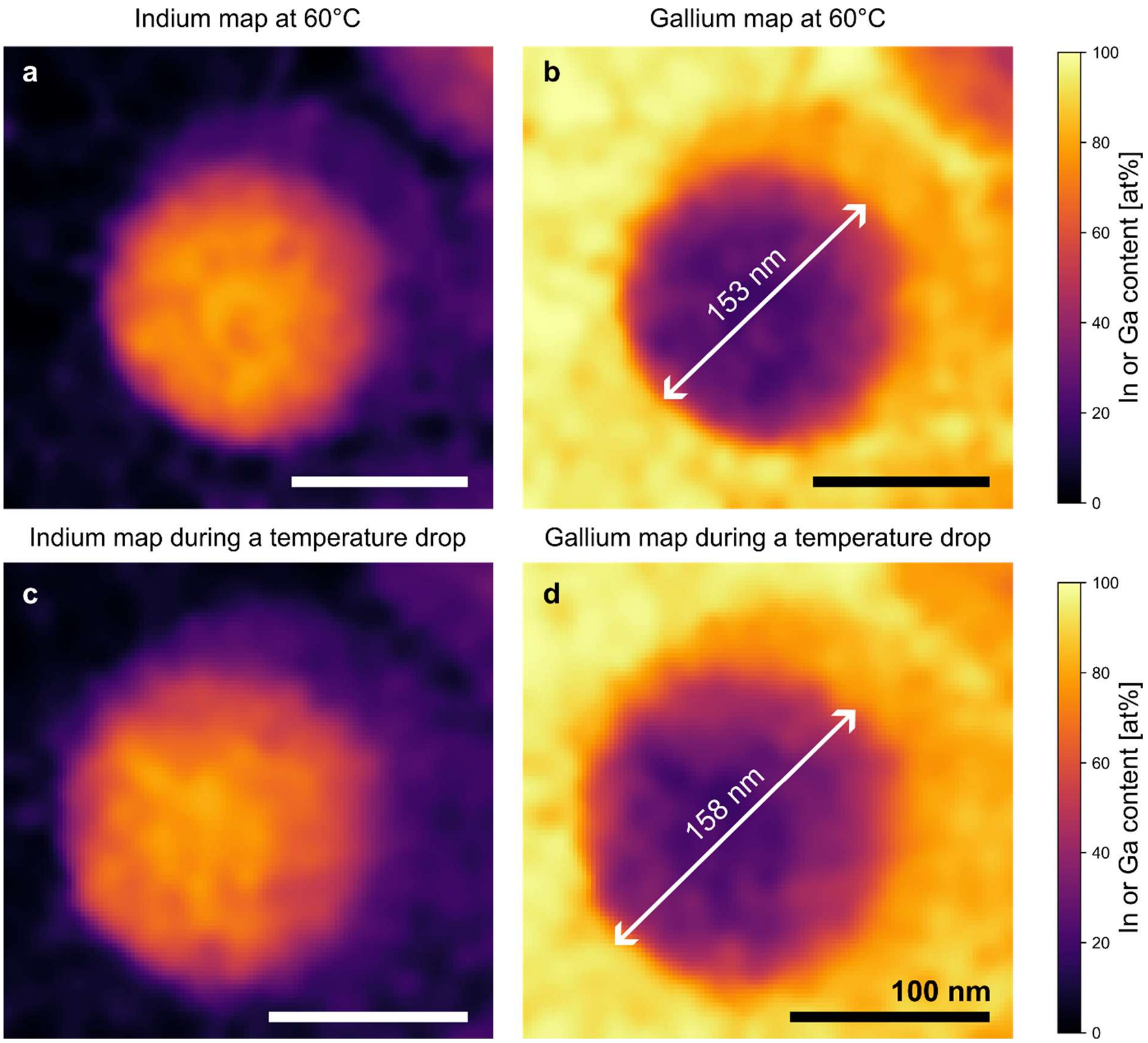

Figure S51 – Reversibility of the dissolution process. Elemental maps of Particle 2 **(a,b)** at 60°C and **(c,d)** during a short temperature drop, showing the immediate radius increase.

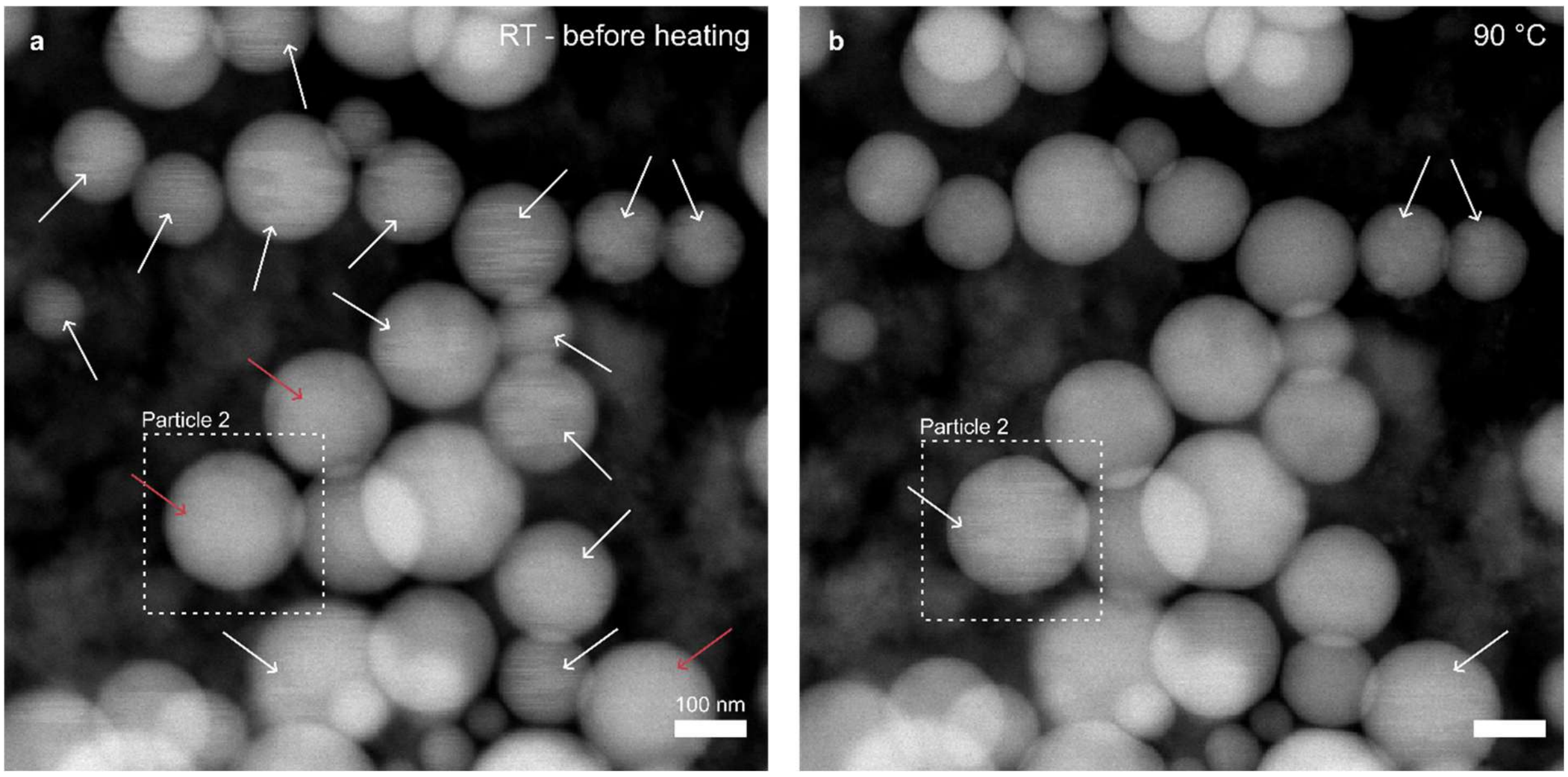

Figure S52 – Core dissolution. HAADF-STEM images **(a)** at room temperature before the beginning of the heating experiment **(b)** at 90°C. The white arrows represent to moving cores. The red arrows represent the immobile cores. At 90°C, almost all the cores were dissolved with gallium, except Particle 2 and a few others.

Note S4 – Simplified model to predict M-InGa structure and composition at different temperatures

To assess how a decrease in the solid core size affects the indium content of the liquid phase, a simplified model was developed based on the following assumptions:

- Assumption 1: both the solid core and the overall particle are spherical and we neglect the existence of the oxide shell.
- Assumption 2: the core consists solely of indium.
- Assumption 3: the density of solid indium is assumed to be identical in the solid and liquid phases.

A schematic of the model can be found below:

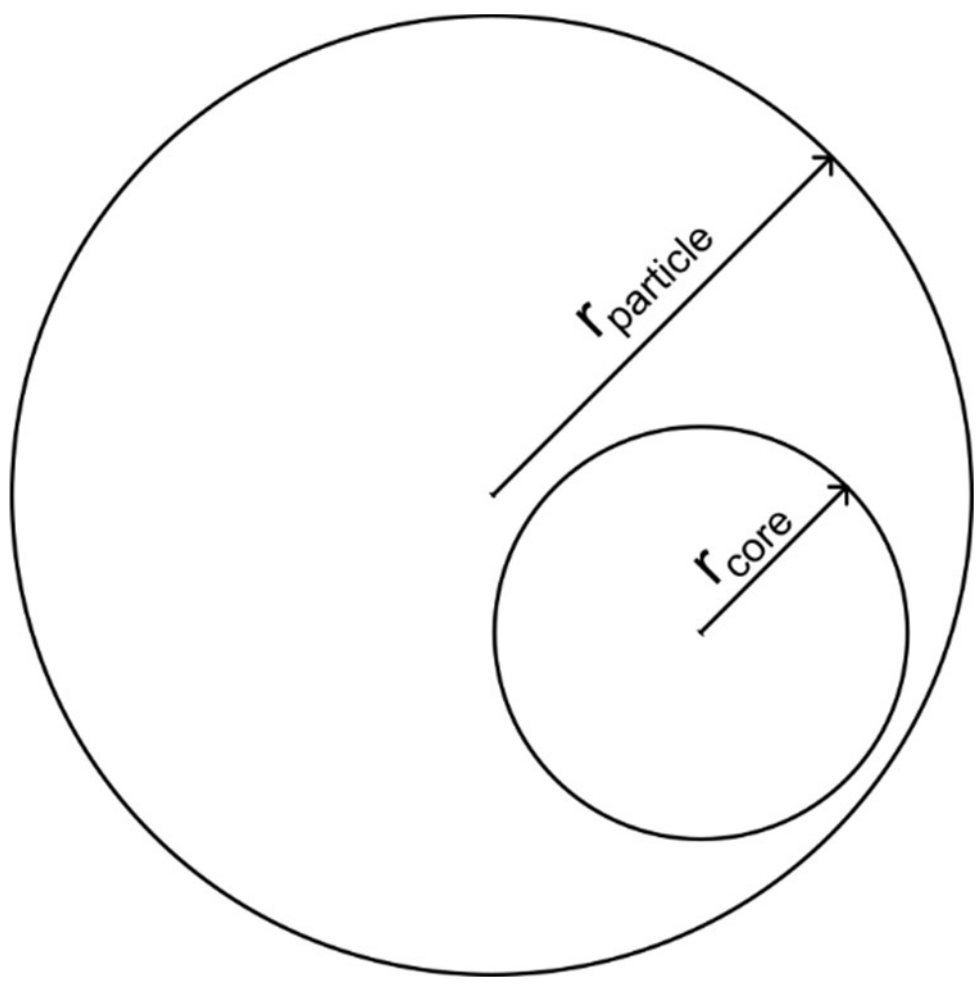

In a first step, we established a relationship between the radius of the overall particle $r_{particle}$ *and its overall composition* $x_{In,particle}$ *and the expected radius of the core* r_{core}.

On one hand, the total amount of indium in the particle n_{In} can be expressed as the sum of the amount in the solid core $n_{In,core}$ and in the liquid phase $n_{In,liq}$,

$$n_{In} = n_{In,core} + n_{In,liq} \tag{1}$$

and the molar fraction of indium in the particle as follows, with n_{Ga} being the total amount of gallium:

$$x_{In,particle} = \frac{n_{In}}{n_{In} + n_{Ga}} \tag{2}$$

With the assumptions 1 and 2, we can write the molar fractions of indium in the liquid phase $x_{In,liq}$ a s follows:

$$x_{In,liq} = \frac{n_{In,liq}}{n_{In,liq} + n_{Ga}} \tag{3}$$

On the other hand, the total volume of the particle $V_{particle}$ and the volume of the core V_{core} can be written as follows, with $n_{In,core}$ being the mole of indium in the core and $\rho_{m,In}$ the molar density of indium.

$$V_{particle} = \frac{4}{3}\pi r_{particle}^3 = \frac{n_{Ga}}{\rho_{m,Ga}} + \frac{n_{In}}{\rho_{m,In}} \tag{4}$$

$$V_{core} = \frac{4}{3}\pi r_{core}^3 = \frac{n_{In,core}}{\rho_{m,In}} \tag{5}$$

With Equation (5), we get:

$$r_{core} = \sqrt[3]{\frac{3}{4\pi} \times \frac{n_{In,core}}{\rho_{In}}} \tag{6}$$

With Equations (1), (2) and (3), we get:

$$n_{In,core} = n_{In} - n_{In,liq} = n_{Ga}\left(\frac{x_{In}}{1 - x_{In}} - \frac{x_{In,liq}}{1 - x_{In,liq}}\right) \tag{7}$$

With Equation (4), we get:

$$n_{Ga} = \frac{V_{particle}}{\frac{1}{\rho_{Ga}} + \frac{1}{\rho_{In}} \times \frac{x_{In}}{1 - x_{In}}} \tag{8}$$

Which gives, overall:

$$r_{core} = \sqrt[3]{\frac{3}{4\pi} \times \frac{n_{Ga}\left(\frac{x_{In}}{1-x_{In}} - \frac{x_{In,liq}}{1-x_{In,liq}}\right)}{\rho_{In}}} = \sqrt[3]{\frac{3}{4\pi} \times \frac{\frac{V_{particle}}{\frac{1}{\rho_{Ga}} + \frac{1}{\rho_{In}} \times \frac{x_{In}}{1-x_{In}}}\left(\frac{x_{In}}{1-x_{In}} - \frac{x_{In,liq}}{1-x_{In,liq}}\right)}{\rho_{In}}} \tag{6}$$

To test the model, we tried to predict the core radius of particles whose overall concentration was known (**Figure S53**). Four NPs of different size and composition were tested. Overall, the predicted core size is rather close to the measured values, with an error increasing with size (from 2% to 15%), maybe due to the fact that bigger NPs are less spherical and more prone to flatten.

In a second step, and in order to assess the effect of a decrease in size of the core on the indium content of the liquid phase, we established a relationship the molar fraction of indium in the liquid phase $x_{In,liq}$ *and a decay in the core radius* r_{decay}.

Using Equations (3) and (4), we get:

$$x_{In,liq} = \frac{n_{In} - V_{core}\rho_{In}}{n_{In} - V_{core}\rho_{In} + n_{Ga}} \tag{7}$$

We can calculate n_{Ga} using Equation (8) and using Equation (2), n_{In} can be expressed as:

$$n_{In} = n_{Ga}\frac{x_{In,particle}}{1 - x_{In,particle}} \tag{8}$$

Which gives the overall equation:

$$x_{In,liq} = \frac{n_{Ga}\frac{x_{In,particle}}{1 - x_{In,particle}} - V_{core}\rho_{In}}{n_{Ga}\frac{x_{In,particle}}{1 - x_{In,particle}} - V_{core}\rho_{In} + n_{Ga}} \tag{9}$$

With Equations (6) and (9) we can now assess the size of the core for a specific composition and particle size, and estimate the effect of a core size decrease on the indium content of the liquid phase. For a particle containing 40 at% of indium (*i.e.* the average composition of **M-InGa**), we can see on the **Figure S54** that initially, a small decrease in the core size leads to a big increase of the indium concentration in the liquid phase, as it corresponds to a big volume decrease. Because the crystalline volume scales with the cube of the radius ($\propto r^3$), whereas the projected area scales with the square of the radius (∝r2) a reduction in radius leads to a much larger relative decrease in volume than in projected area. Consequently, XRD patterns (which depend on the crystalline volume) are expected to be more strongly affected at early stages of core dissolution than HAADFT-STEM imaging, which observe the projected area.

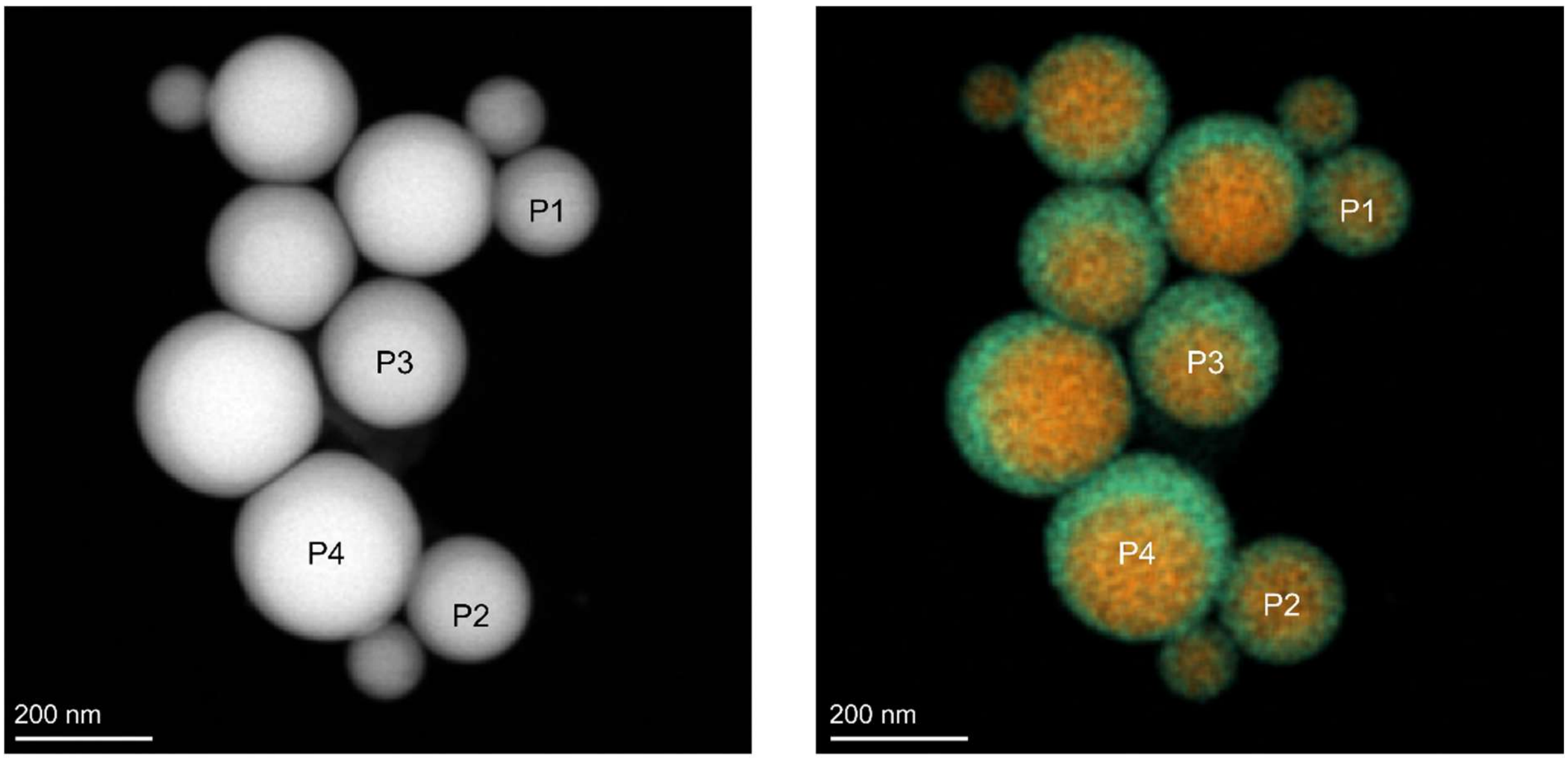

P1:
Indium content: 43.51 at%
Particle size: 155 nm

Predicted core size: 111 nm
Measured core size: 109 nm
Error: 2%

P2:
Indium content: 51.41 at%
Particle size: 182 nm

Predicted core size: 142 nm
Measured core size: 148 nm
Error: 4%

P3:
Indium content: 42.83 at%
Particle size: 270 nm

Predicted core size: 193 nm
Measured core size: 209 nm
Error: 8%

P4:
Indium content: 47.73 at%
Particle size: 217 nm

Predicted core size: 162 nm
Measured core size: 191 nm
Error: 15%

Figure S53 – Testing of the two-spheres model for predicting the core size of In-Ga NPs. The core size was predicted based on the size and composition of each NP. The indium content of the liquid phase was assumed to be 17 at% based on the values found in **Figure S14** and **Figure S31.**

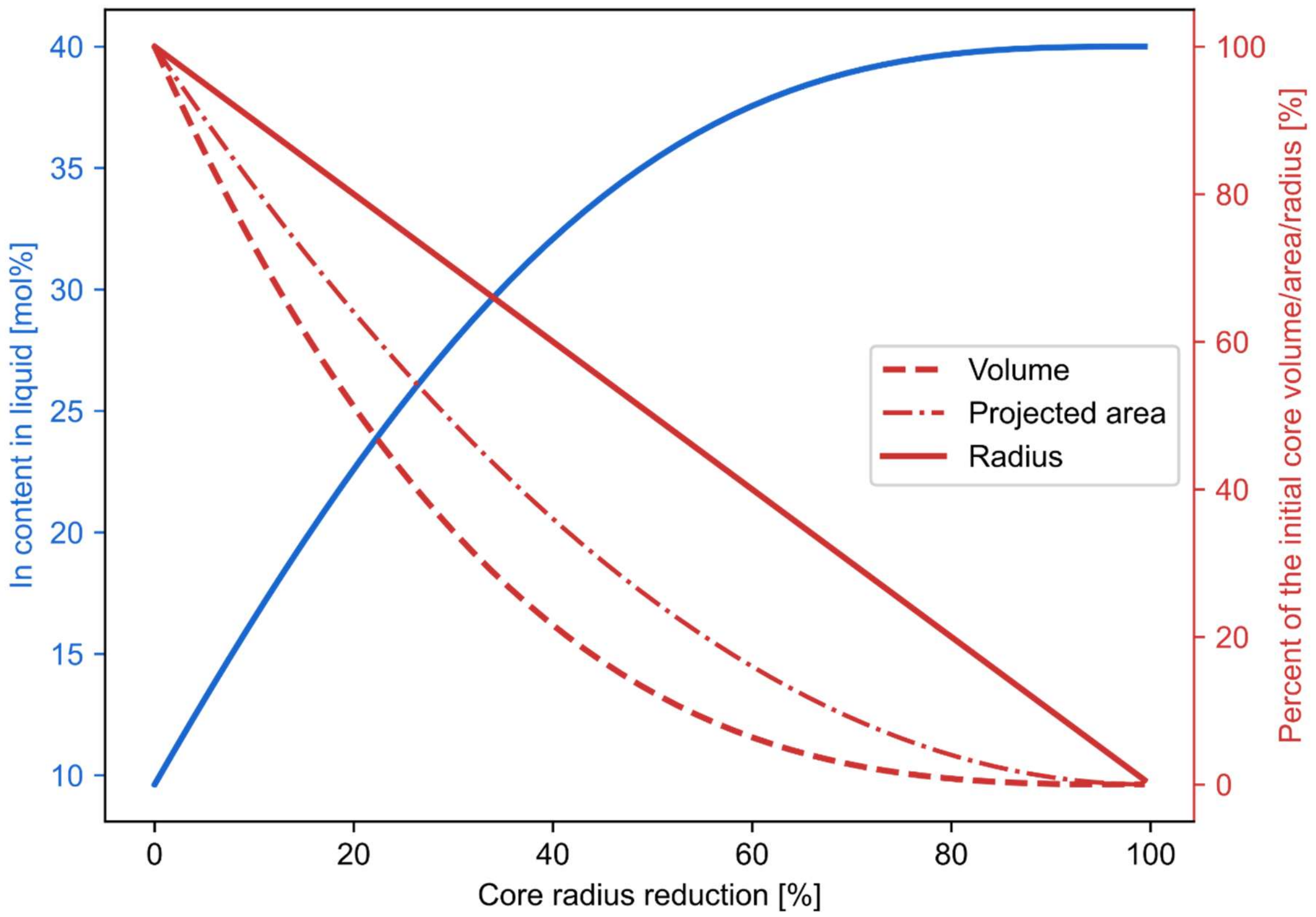

Figure S54 – Effect of a core size reduction on the indium content of the liquid phase for M-InGa. This plot is based on the model developed in **Note S4**. The projected area corresponds to what is observed with STEM-HAADF.

Note S5 – Discussion on the intrinsic activity of the InGa NPs.

Reliable normalization of electrocatalytic rates is crucial for enabling meaningful comparisons of catalytic performance and for separating true improvements in intrinsic activity from variations caused by extrinsic factors, such as catalyst loading, particle size, or morphology.[12] In the main manuscript, we used a constant (gallium + indium) mass loading and opted for a normalization by the electrode geometric area (**Figure S55a**). In this note, we discuss the effect of various normalization on the CO_2RR current.

Working with gallium-based NPs supported on carbon black poses challenges in measuring the electrochemically active surface area (ECSA), which is the preferred normalization when interested in intrinsic electrocatalyst activity.[12] Gallium possesses an oxide shell in all the voltage regions where no Faradaic processes occur. The poor electrical conductivity of the gallium oxide renders unreliable the measurement of the double-layer capacitance for the determination of the ECSA.[13,14] In the case of oxide electrocatalysts, impedance measurements with the implementation of equivalent electric circuit have been developed, yet this circuit remains to be established reliably for our system.[15–17] The presence of carbon black adds an additional level of complexity when using electrochemical methods for surface area determination.

When well-defined NPs are used as catalysts, the sum of the surfaces of all the individual NPs surface area of the catalyst is often used as a good approximation of the active area.[6,14,18] The average size can be determined using electron microscopy. In the case of our In-Ga NPs, the surface *S* would be defined as follows:

$$S = N_{NP}S_{NP} = \frac{V_{tot}}{V_{NP}}4\pi r^2 = \left(\frac{m_{In}}{\rho_{In}} + \frac{m_{Ga}}{\rho_{Ga}}\right)\frac{6}{d}$$

With N_{NP}being the number of NPs on the electrode, S_{NP} the average surface of each NP [cm^2], V_{tot} the total volume of material [cm^3], *r* the average radius [cm], m_i the mass of the element *i* [g], ρ_i the volumetric mass density [g/cm^3] and *d* the average NP size [cm].

The CO_2RR current density normalized by the surface *S* is plotted in **Figure S55b.** Overall, the trends are very similar to the **Figure S55a**, which is expected because the average NP sizes are rather similar for all catalysts. Linking the normalized current density to the intrinsic catalytic activity, these data point towards a higher intrinsic activity for **M-InGa**, which increases with

temperature and peaks at 70 °C, compared to **L-InGa** and to **S-InGa**, which exhibits the lowest activity based on this normalization.

Going a step further towards normalizing the current density by the actual number of active sites, we discuss now two additional methods, which are reported in **Figure S55c** and **d**.

We focus on the indium active sites generating formate and make the assumption that the contribution of gallium to formate production is negligible (**Figure S24**), which is reasonable considering indium alone is selective for formate and gallium for CO.

Thus, we use the mass of indium on the electrode to normalize the current (**Figure S55c**). The results are interesting. We can again assign to **S-InGa** the lowest intrinsic activity. **L-InGa** appears to be most intrinsically active catalyst at low temperature up to 60 °C, **M-InGa** becomes slightly better and/or similar at higher temperatures.

Normalizing the activity by the total indium mass implicitly assumes a homogeneous catalyst composition and, thus, that all indium species (solid indium or solubilized indium in **M-InGa**) contribute equally to the catalytic activity. Yet, we know that **M-InGa** possesses a core-shell structure with most of the indium concentrated in the solid inner core at lower temperature which progressively dissolves in the gallium as temperature increases. Therefore, normalizing the activity by the total indium mass effectively overestimates the number of active sites available in **M-InGa**, resulting in underestimating the intrinsic activity.

Thus, we decided to use the concentration of solubilized indium at different temperatures determined by STEM-EDXS to normalize the CO_2RR current for **M-InGa**. The resulting normalized currents are presented in **Figure S55d**. This normalization results in **L-** and **M-InGa** overlapping at low temperatures up to 40 °C. As temperature increases, **M-InGa** deviates with an apparent higher intrinsic activity. However, we retain ourselves from making a strong conclusion here. Indeed, we note that normalizing the activity by the mass of dissolved indium assumes that the indium is homogeneously distributed throughout the liquid phase. XPS analysis in **Figure S27** and **Figure S28** indicates that the surface becomes enriched in indium after CO_2RR, meaning that the surface concentration of catalytically active indium may differ from the bulk composition. Thus, we envision future in-situ XPS measurement at increased temperatures to precisely determine the amount of surface indium atoms to eventually conclude about the enhanced intrinsic activity of the solubilized indium active sites in **M-InGa**.

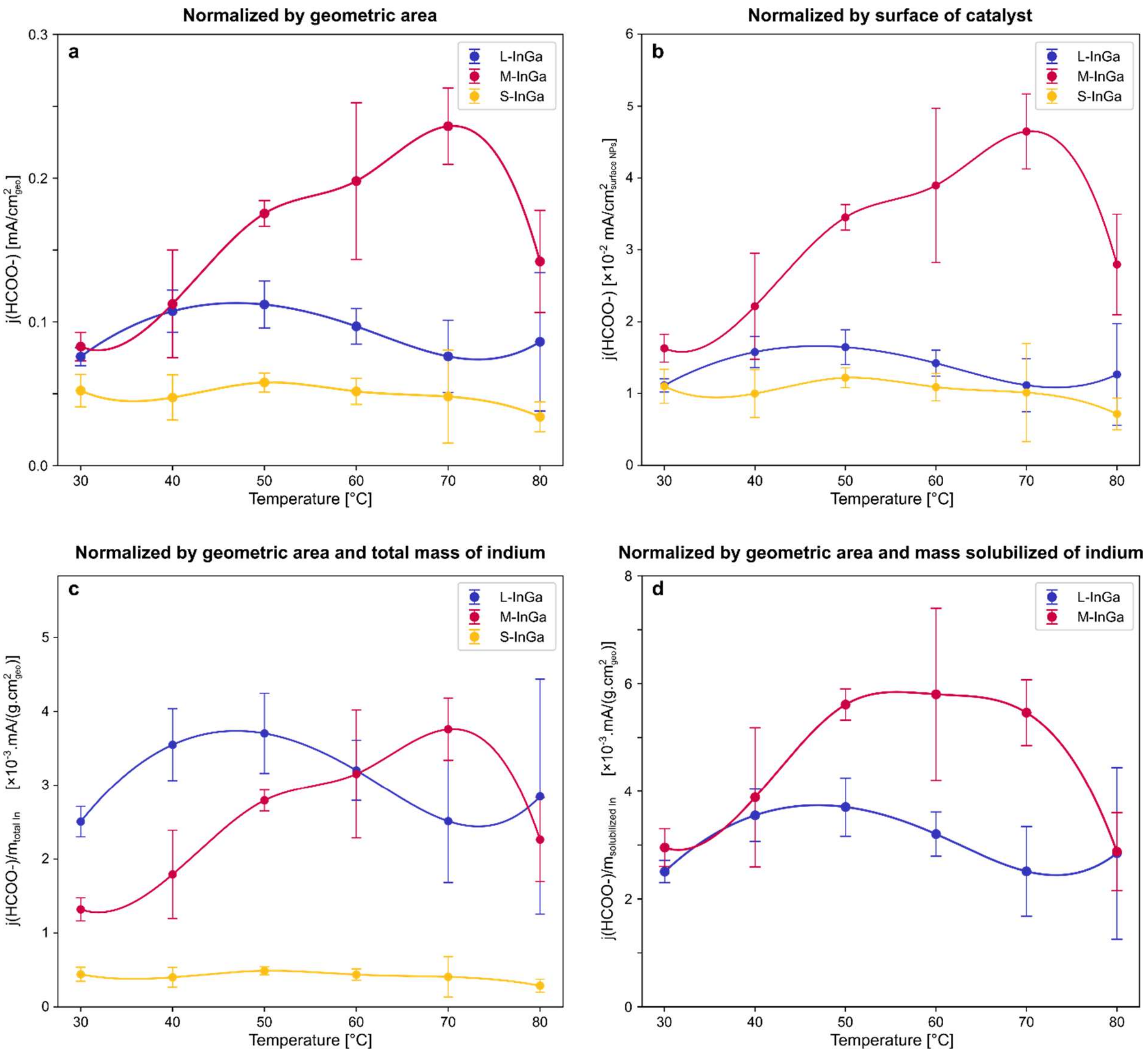

Figure S55 – Normalized formate partial current densities. Formate partial currents normalized by the geometric area (**a**), by the surface of catalyst (**b**), by the total mass of indium (**c**) and by mass of solubilized indium (**d**).

References

(1) Yarema, M.; Wörle, M.; Rossell, M. D.; Erni, R.; Caputo, R.; Protesescu, L.; Kravchyk, K. V.; Dirin, D. N.; Lienau, K.; von Rohr, F.; Schilling, A.; Nachtegaal, M.; Kovalenko, M. V. Monodisperse Colloidal Gallium Nanoparticles: Synthesis, Low Temperature Crystallization, Surface Plasmon Resonance and Li-Ion Storage. *J. Am. Chem. Soc.* **2014**, *136* (35), 12422–12430. https://doi.org/10.1021/ja506712d.

(2) Torruella, P.; Halimi, A.; Tovaglieri, L.; Lichtensteiger, C.; Alexander, D. T. L.; Hébert, C. A Multiscale Bayesian Approach to Quantification and Denoising of Energy-Dispersive x-Ray Data. *Mach. Learn. Sci. Technol.* **2025**, *6* (2), 025043. https://doi.org/10.1088/2632-2153/add8e1.

(3) Walt, S. van der; Schönberger, J. L.; Nunez-Iglesias, J.; Boulogne, F.; Warner, J. D.; Yager, N.; Gouillart, E.; Yu, T. Scikit-Image: Image Processing in Python. *PeerJ* **2014**, *2*, e453. https://doi.org/10.7717/peerj.453.

(4) Kumar, K.; Loiudice, A.; Boulanger, C.; Chabok, S.; Buonsanti, R. Real-Time Monitoring Reveals Stability of Gallium Nanoparticles beyond the Thermodynamic Reduction Potential of Their Oxide Skin under CO2 Reduction Conditions. *Chem. Mater.* **2025**, *37* (19), 7952–7961. https://doi.org/10.1021/acs.chemmater.5c01734.

(5) Pankhurst, J. R.; Iyengar, P.; Loiudice, A.; Mensi, M.; Buonsanti, R. Metal–Ligand Bond Strength Determines the Fate of Organic Ligands on the Catalyst Surface during the Electrochemical CO2 Reduction Reaction. *Chem. Sci.* **2020**, *11* (34), 9296–9302. https://doi.org/10.1039/D0SC03061A.

(6) Boulanger, C.; Arenas Esteban, D.; Kumar, K.; Leemans, J.; Albertini, P. P.; You, H.; Assendjee, E.; Stoian, D. C.; Bals, S.; Buonsanti, R. A Colloidal Approach for the Synthesis of Catalytically Active Solid–Liquid–Solid Nanoparticles. *Nat. Synth.* **2025**, 1–9. https://doi.org/10.1038/s44160-025-00871-y.

(7) Anderson, T. J.; Ansara, I. The Ga-In (Gallium-Indium) System. *J. Phase Equilibria* **1991**, *12* (1), 64–72. https://doi.org/10.1007/BF02663677.

(8) Clarysse, J.; Moser, A.; Yarema, O.; Wood, V.; Yarema, M. Size- and Composition-Controlled Intermetallic Nanocrystals via Amalgamation Seeded Growth. *Sci. Adv.* **2021**, *7* (31), eabg1934. https://doi.org/10.1126/sciadv.abg1934.

(9) Castilla-Amorós, L.; Stoian, D.; Pankhurst, J. R.; Varandili, S. B.; Buonsanti, R. Exploring the Chemical Reactivity of Gallium Liquid Metal Nanoparticles in Galvanic Replacement. *J. Am. Chem. Soc.* **2020**, *142* (45), 19283–19290. https://doi.org/10.1021/jacs.0c09458.

(10) Anderson, B. D.; Tracy, J. B. Nanoparticle Conversion Chemistry: Kirkendall Effect, Galvanic Exchange, and Anion Exchange. *Nanoscale* **2014**, *6* (21), 12195–12216. https://doi.org/10.1039/C4NR02025A.

(11) He, M.; Protesescu, L.; Caputo, R.; Krumeich, F.; Kovalenko, M. V. A General Synthesis Strategy for Monodisperse Metallic and Metalloid Nanoparticles (In, Ga, Bi, Sb, Zn, Cu, Sn, and Their Alloys) via in Situ Formed Metal Long-Chain Amides. *Chem. Mater.* **2015**, *27* (2), 635–647. https://doi.org/10.1021/cm5045144.

(12) Bates, J. S.; Silva Alves Carneiro, J.; Clark, E. L.; Harlow, G. S.; Stoerzinger, K. A.; Strasser, P.; Stuve, E. M.; Seitz, L. C.; Resasco, J. Rigor & Reproducibility in Electrocatalysis: Approaches and Considerations for Surface Area Normalization. *ACS Catal.* **2026**. https://doi.org/10.1021/acscatal.6c01834.

(13) Wei, C.; Sun, S.; Mandler, D.; Wang, X.; Qiao, S. Z.; Xu, Z. J. Approaches for Measuring the Surface Areas of Metal Oxide Electrocatalysts for Determining Their Intrinsic Electrocatalytic Activity. *Chem. Soc. Rev.* **2019**, *48* (9), 2518–2534. https://doi.org/10.1039/C8CS00848E.

(14) Okatenko, V.; Castilla-Amorós, L.; Stoian, D. C.; Vávra, J.; Loiudice, A.; Buonsanti, R. The Native Oxide Skin of Liquid Metal Ga Nanoparticles Prevents Their Rapid Coalescence during Electrocatalysis. *J. Am. Chem. Soc.* **2022**, *144* (22), 10053–10063. https://doi.org/10.1021/jacs.2c03698.
(15) Doyle, R. L.; Lyons, M. E. G. An Electrochemical Impedance Study of the Oxygen Evolution Reaction at Hydrous Iron Oxide in Base. *Phys. Chem. Chem. Phys.* **2013**, *15* (14), 5224–5237. https://doi.org/10.1039/c3cp43464h.
(16) Lyons, M. E. G.; Brandon, M. P. The Significance of Electrochemical Impedance Spectra Recorded during Active Oxygen Evolution for Oxide Covered Ni, Co and Fe Electrodes in Alkaline Solution. *J. Electroanal. Chem.* **2009**, *631* (1), 62–70. https://doi.org/10.1016/j.jelechem.2009.03.019.
(17) Moysiadou, A.; Lee, S.; Hsu, C.-S.; Chen, H. M.; Hu, X. Mechanism of Oxygen Evolution Catalyzed by Cobalt Oxyhydroxide: Cobalt Superoxide Species as a Key Intermediate and Dioxygen Release as a Rate-Determining Step. *J. Am. Chem. Soc.* **2020**, *142* (27), 11901–11914. https://doi.org/10.1021/jacs.0c04867.
(18) Reske, R.; Mistry, H.; Behafarid, F.; Roldan Cuenya, B.; Strasser, P. Particle Size Effects in the Catalytic Electroreduction of CO2 on Cu Nanoparticles. *J. Am. Chem. Soc.* **2014**, *136* (19), 6978–6986. https://doi.org/10.1021/ja500328k.
